# Unraveling the bioactive constituents of *Typha elephantina*: A comprehensive phytochemical analysis by tandem mass spectrometry

**DOI:** 10.1371/journal.pone.0311549

**Published:** 2024-12-05

**Authors:** Bibi Rohida, Muhammad Farman, Alina George

**Affiliations:** Department of Chemistry, Quaid-i-Azam University Islamabad, Islamabad, Pakistan; University of Brescia: Universita degli Studi di Brescia, ITALY

## Abstract

Phytochemicals derived from plants have gained significant attention in recent years due to their diverse therapeutic properties. *Typha elephantina* is an aquatic plant having ameliorative characteristics like antioxidant, anti-inflammatory and analgesic etc. This research aims to conduct a comprehensive phytochemical investigation by Tandem mass spectrometry on the aerial parts and roots of *Typha elephantina* with a focus on identifying and characterizing the bioactive compounds present in it. Maceration in methanol, preliminary, MS/MS analyses and DPPH antioxidant assay were carried out on this plant. This study led to the elucidation of 62 chemical constituents for the first time in *Typha elephantina*. 36 phytochemical compounds from aerial parts and 26 from roots *i*.*e*.,*p*-coumaric acid, caffeic acid, dihydrocaffeic acid, ferulic acid derivative, dehydroascorbic acid derivative, 1-*O*-coumaroyl glycerol, glucaroyl-4-hydroxy benzoate, apigenin derivative, 3-*O*-glucopyranosyl isorhamnetin, isovitexin derivative, rutin, isorhamnetin diglycosides, verbascoside, forsythoside A, pinocembrin, dihydro quercetin, prunetin, ampelopsin, daidzein, genistein, catechin and procyanidin B1 were detected within this plant specimen. The DPPH assay results showed that aerial parts TE(1), TE(2) showed more antioxidant activity than roots TER/MeOH. These might be responsible for the understanding of the therapeutic potential of *Typha elephantina* and provide a foundation for future pharmacological studies.

## 1. Introduction

Researchers have studied natural plant-based products that are used in the food, cosmetic, and pharmaceutical industries. People have been consuming medicinal and aromatic herbs for centuries as antioxidants, dietary supplements, and defense against diseases like cancer. Due to the presence of vital nutrients, aromatic, and bioactive phytochemical components, medicinally, aromatically, and nutritionally significant plants have piqued the interest of researchers. Because of the widespread use of so-called plant species and growing public interest in the existence of particular compound groups with advantageous health effects, functionalized food products are being produced. In this regard, thousands of phytochemical substances from various chemical classes to be investigated to detect the target compounds for their beneficial uses in food industry, medicine and cosmetics [1, 2].

Because of the high calibre, reliability, and efficacy of herbal therapy, ethnomedical plants have been used in phytotherapy treatments. *Priva cordifolia* (L.f) Druce plant antioxidant characteristics are to be examined, along with the evaluation of the various extracts (16 extracts) obtained from leaf, stem bark, root, and seeds by enhanced polarity solvent-based extraction. The gallic acid equivalent (GAE) of total polyphenols and flavonoids ranges from 20.60 ± 0.19–148.20 ± 1.52 mg/g and 7.31 ± 0.09–350.46 ± 12.80 mg/g, respectively. The DPPH, ABTS scavenging radical, and FRAP activity of each extract have been evaluated. The leaf extracts in acetone and methanol had the best activity among them. Antibacterial and antioxidant properties of the leaf extract were investigated after further fractionation. HRLC-MS was used to identify a total of 27 metabolites. Every metabolite that was discovered underwent testing for the Blood-Brain Barrier, ADME, pharmacophore model, and molecular docking analysis. With this insilico approach, every molecule has a decent docking score and binding energy to the specific protein target, which is the DNA gyrase protein (3G7B), ranging from − 6 to– 10.When the fraction was tested further for cytotoxicity against L6 normal cells, the results showed negligible toxicity. The methanolic extract of the leaf of *P*. *cordifolia* revealed a wide range of phytochemicals [3] Genus Typha is pharmacologically potent due to the presence of phytoconstituents like stigma sterols [4] carboxylic acids, phenolic acids, [5] sterol glycosides, flavonoids, flavonoid glycosides, [6–9] coumarates, ferulates [10], glycerolipids, alkaloids and cerebrosides [9, 11] which paves the way to work on it. Biological activities like anti-atherogenic effect [12], keratinocytes proliferation [13], hepatoprotective, antidiabetic [14], anti-collegenase [15] and thrombolytic [16] highlight the therapeutic potential of Typha.

*Typha elephantina* Roxb. (Cattails) is aperennial aquatic plant [17] having long roots [18] and is widely distributed in Iran, Pakistan, Nepal, India and North Africa [19]. It has various curative activities including membrane stabilizing potential, thrombolytic, anthelmintic, antioxidant, anxiolytic, wound healing, anti-inflammatory, analgesic, cytotoxic, hypoglycemic, hepatoprotective and hematopoietic potential [20, 21].

A sufficient gap in literature was exploredthe structural characterization of bioactive molecules in this species by spectrometric analysis. Therefore, this study was aimed to qualify and quantify complete profile of phytochemicals from *Typha elephantina* byMS/MS (Tandem mass spectrometry) technique.

## 2. Materials and methodology

### 2.1. Plan of work

**Fig 1:**
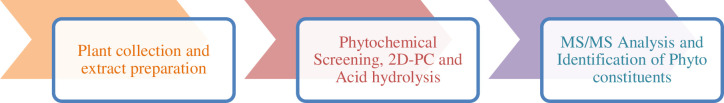
The pictorial representation for plan of work. Fig 1 showed the plan of work for Typha elephantina complete profiling.

The plan of work was explained by **Fig 1**. In which the strategy was mentioned which were followed for the *Typha elephantina* complete profiling.

### 2.2. Collection and identification of plant

Fresh *Typha elephantina* (Kondr, Lukhy) was collected from Zhob (daeragi pull, bhatyie) Balochistan to ensure the availability of diverse phytochemicals.It’s authentication was done from Department of Botany SBK women’s university Quetta and also from Plant Sciences Department Quaid-i-Azam University, Islamabad.

### 2.3. Extraction

The *Typha elephantina* aerial partsextract were prepared by maceration of 2100g of plant material in 15L MeOH for two months. The infusions were filtered and filtrate was evaporated to dryness using Heidolph 4000-efficient rotary evaporator. As a result, crude extract in the form of syrupy liquid was obtained and were divided in to two parts TE(1)/MeOH (brown colour, *Typha elephantina* aerial parts extract), TE(2)/MeOH(green colour *Typha elephantina* aerial parts extract)

The *Typha elephantina* roots extract was prepared by maceration of 5650g of roots material in 21L MeOH for two months. By using Heidolph 4000-efficient rotary evaporator filtrate was evaporated to dryness. As a result, TER/MeOH (*Typha elephantina* Root extract)was obtained as syrupy liquid.

### 2.4. Preliminary analyses

Initial assessment encompassing phytochemical screening, 2D-PC analysis and acid hydrolysis were performed on the plant extract.

#### 2.4.1. Phytochemical screening

Phytochemical screening was employed for identification of the main classes of compounds (steroids, terpenoids, tannins, saponins, phenolic acids, alkaloids and flavonoids in *Typha elephantina*.The protocol used for phytochemical tests was similar to the one mentioned by [22].

#### 2.4.2. Two-dimensional paper chromatography

2D-PC was carried out to identify the phenolic compounds present in the plant extract. The stationary phase used was Whatman filter paper No. 1 (20×20cm). The sample loaded and dried chromatographic paper was developed in 1st dimension i.e., BAW (n-Butanol: Acetic acid: Water 4:1:5, upper layer). For second dimension, the chromatogram was irrigated in 15% Acetic acid. After the development to three-fourth of the paper chromatogram, it was removed, air-dried and visualized under UV lamp. Consequently, the separated bands were marked and their R_*f*_ values were calculated.

#### 2.4.3. Acid hydrolysis and Co-TLC

Acid hydrolysis was performed on 2g of TE(1), 1g of TE(2)/MeOH and 2g of TER/MeOH of *Typha elephantina* extract. It was added to 2N HCl and methanol. The resulting mixture was refluxed on a boiling water bath for 7 hours at 110°C. The aqueous layer containing the glycan moieties was extracted with ethyl acetate in a separating funnel to separate the sugars from aglycones.The Co-TLC was done using silica gel TLC plate 60F_254_Merck (20×20 cm) impregnated with 0.2 M sodium dihydrogen phosphate. The hydrolysate containing the sugars was loaded on the base line of impregnated TLC plate alongside the reference sugars. The TLC plate was developed in Acetone: Water (9:1) as mobile phase. After drying, the TLC plate was sprayed with Aniline Hydrogen Phthalate and heated in the oven at 120°C until brownish spots appeared on it. The R_*f*_values were calculated and compared with the R_*f*_ of standard sugars to identify the sugars present in the hydrolysate.

### 2.5. Tandem mass spectrometry analysis

MS/MS analysis, a comprehensive CID based technique was used for the analysis of samples TE (1)/MeOH, TE(2)/MeOH, TER/MeOH to break down it in the form of precursor ions (MS^1^) followed by Collision Induced Dissociation to produce product ions (MS^2^).

#### 2.5.1. Instrumental parameters

The fragmentation experiments by Linear Ion Trap Mass spectrometer were performed using LTQXL (Thermo Electron Scientific, USA) equipped with electro spray ionization (ESI) source. Methanol was used as a solvent, mode of injection was direct insertion method flow rate for sample was 10μL/min. The negative mode of ionization was employed for scanning with 4.2kV capillary voltage at 280°C. Nitrogen was used as both thesheath gas and auxiliary gas at 20 and 5 arbitrary units respectively. Mass spectral data were acquired over 50–2000 *m/z* scanning mass range. Helium was used as a damping and collision gas at a partial pressure of 50 psi. Fragmentation was accomplished using Collision Induced Dissociation (CID). The relative collision energies employed was from 20 to 30 eV. The software used for data acquisition was Xcalibur 2.0.7.

### 2.6. Sample preparation

A clear sample solution of TE(1)/MeOH, TE(2)/MeOH, TER/MeOH were sent for MS/MS analysis in order to inquire the cluster of phenolic compounds in it.

### 2.7. DPPH radical scavenging activity

The DPPH activity was performed by using 1,1-Diphenyl-2-picrylhydrazine solution (9.6mg/100ml MeOH). Ascorbic acid was used as positive control. The 10μl samples TE(1), TE(2), TER/MeOH were loaded in 90well plate and 190 μl DPPH reagent was added. The absorption was observed by UV-vis microplate reader.

## 3. Results, discussion and conclusion

### 3.1. The preliminary analyses of TE(1)/MeOH, TE(2)/MeOH and TER/MeOH

The extraction process yielded 103.26 grams, 23.26 grams and 622.14 grams dried weight mass of the plant material of TE(1)/MeOH, TE(2)/MeOH and TER/MeOH. The phytochemical tests performed and the chemical constituents identified in TE(1)/MeOH, TE(2)/MeOH and TER/MeOH extract are enlisted in **Table 1**. The phytochemical screening of *Typha elephantina* showed that this species is enriched with a wide variety of bioactive compounds like carbohydrates, proteins, phenolic acids, saponins, alkaloids, glycosides, steroids and flavonoids. Moreover, triterpenoids were absent in TE(1)/MeOH, TE(2)/MeOH and TER/MeOH.

**Table 1:** Phytochemical screening of methanolic extract TE(1)/MeOH, TE(2)/MeOH, TER/MeOH.

S.No	Phytochemicals	Phytochemical Test	*Typha elephantina* TE(1)/MeOH	*Typha elephantina* TE(2)/MeOH	*Typha elephantina* TER/MeOH
1.	Carbohydrates	Molisch Test	+	+	+
2.	Triterpenoids	Horizon Test	-	-	-
3.	Protein	Biuret	+	+	+
4.	Tannins and phenols	a. FeCl_3_ b. Lead acetate	+ +	+ +	+ +
5.	Saponin	Frothing	+	+	+
6.	Alkaloids	a) Dragendorff b) Mayers c) Hager	+ + +	+ + +	+ + +
7.	Glycosides	Keller-killani Test	+	+	+
8.	Terpenes and Steroids	Salkowski Test	+	+	+
9.	Starch	Iodine test	+	+	+
10.	Flavonoids	a) Alkaline Reagent b) Shinoda Test	+ +	+ +	+ +

**Fig 2:**
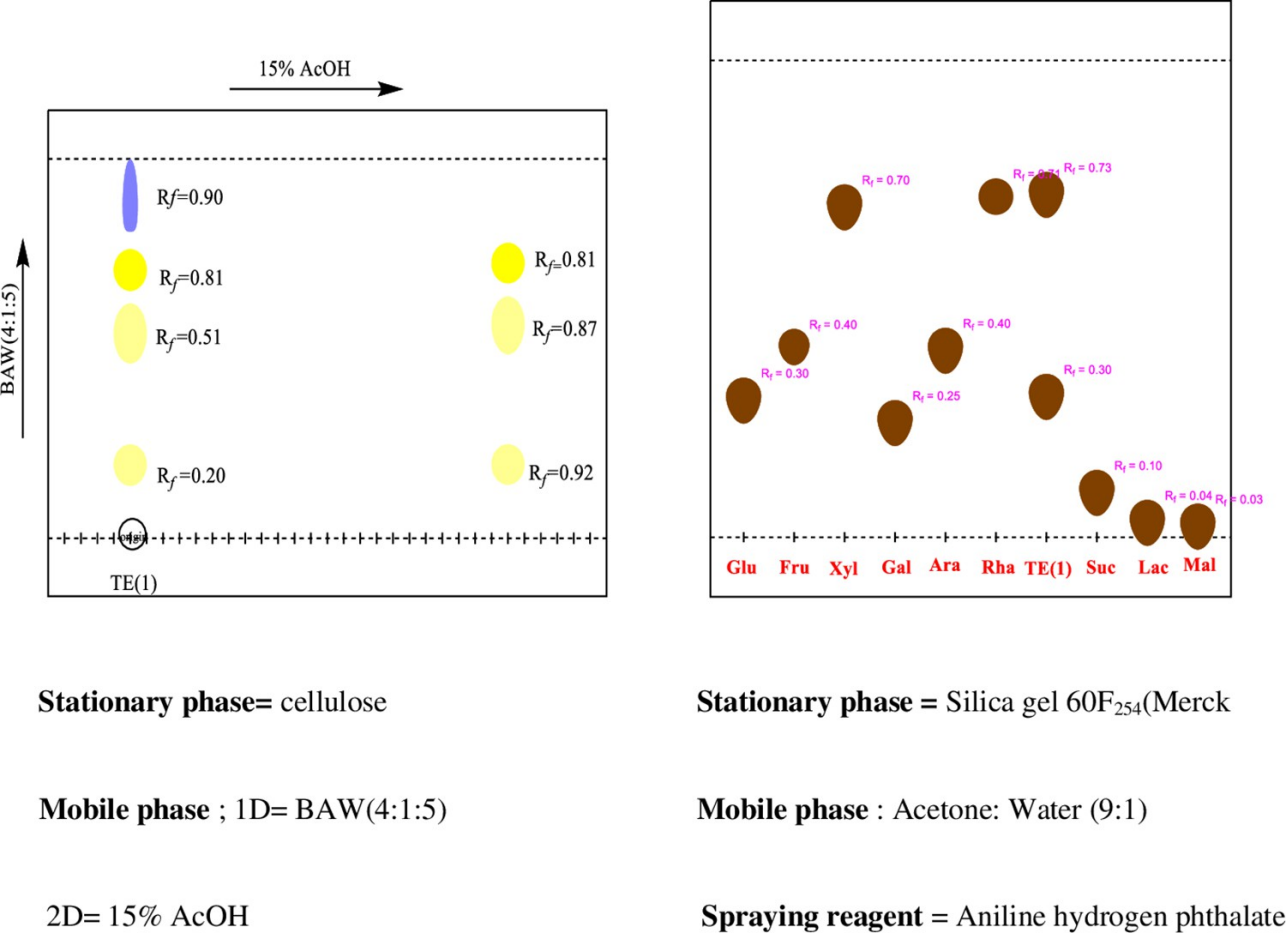
The 2D-PC and TLC chromatogram of TE(1)/MeOH.

**Fig 3:**
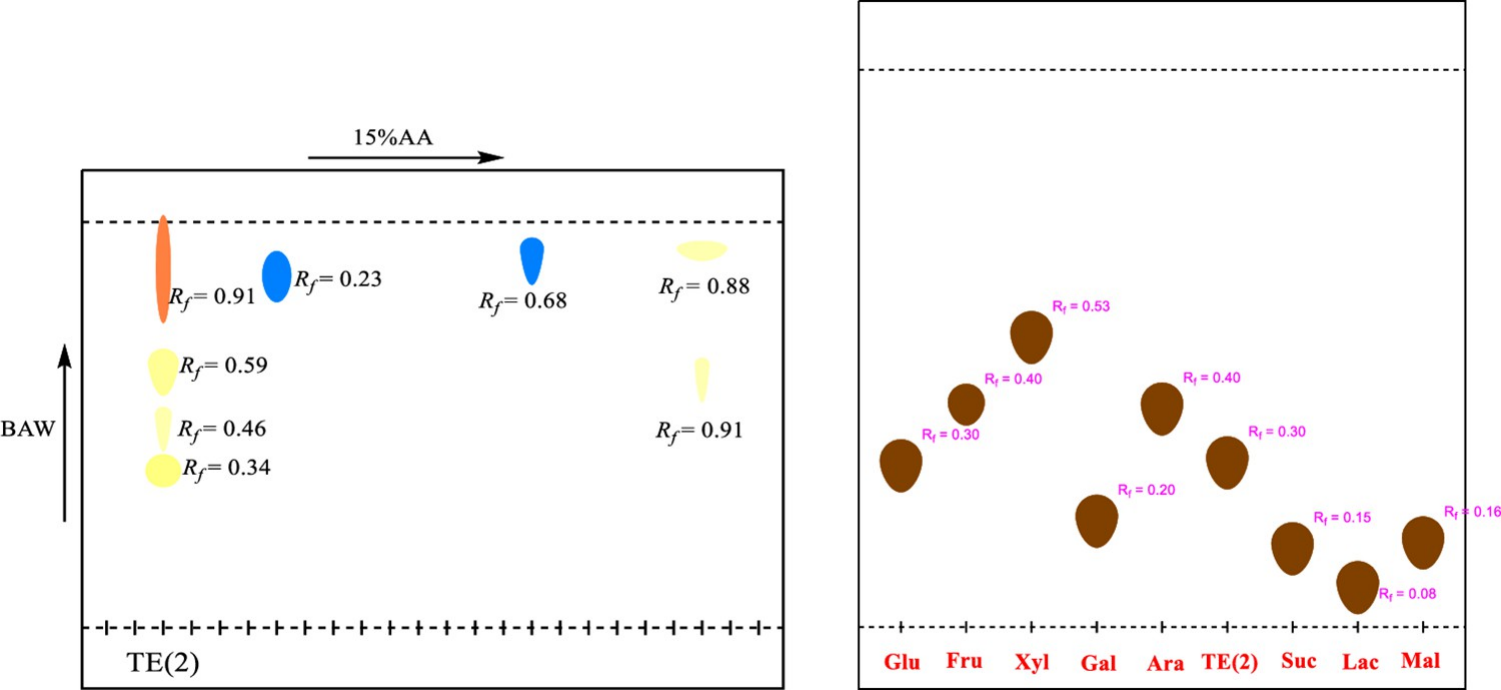
The 2D-PC and TLC chromatogram of TE(2)/MeOH.

**Fig 4:**
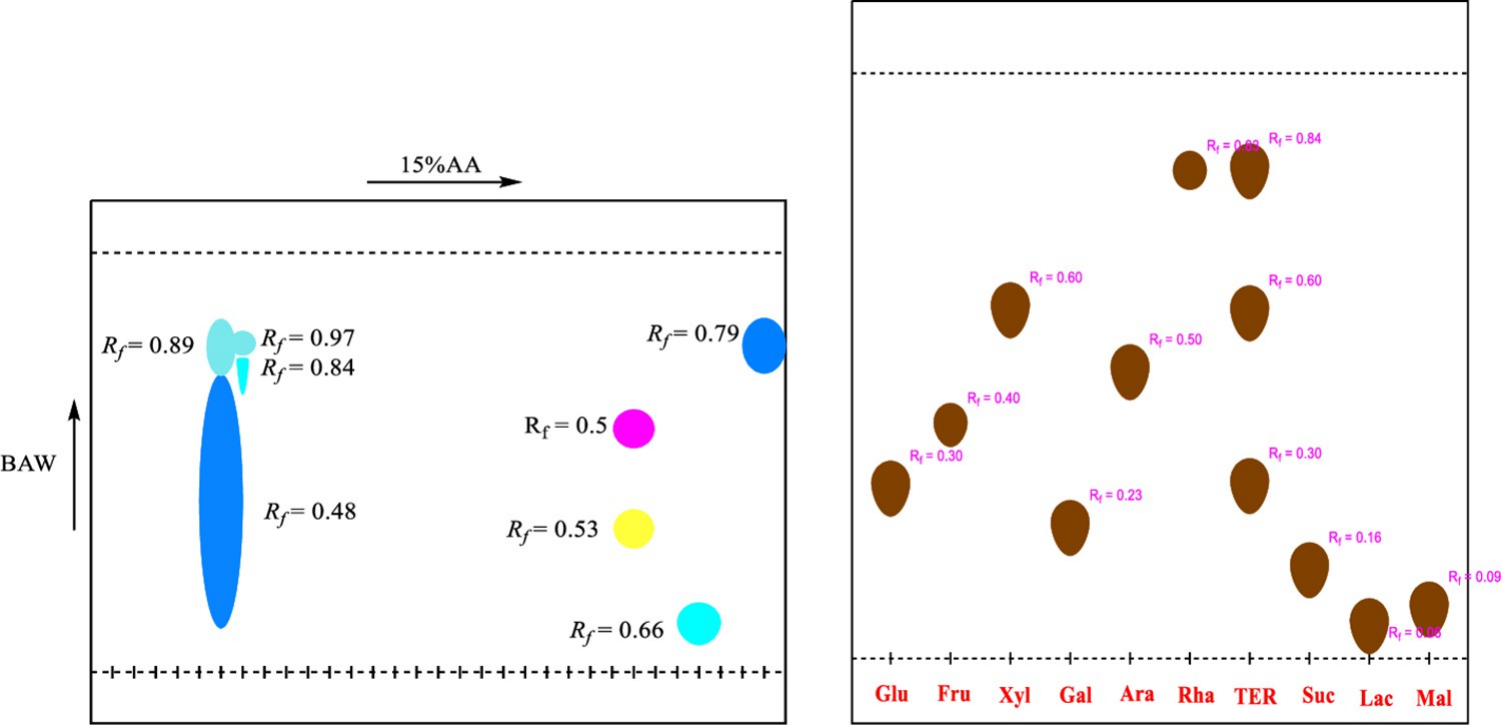
The 2D-PC and TLC chromatogram of TER/MeOH. Figs 2–4 indicated the chromatogram of 2D-PC and TLC for TE(1), TE(2) and TER/MeOH.

2D-PC analyses provided information regarding phytoconstituents in the plant extract. In 1^st^ dimension, two spots of light yellow color, a dark yellow and a blue colored spot with R_*f*_ = 0.20, 0.51, 0.81 and 0.90 respectively were observed. In the 2^nd^ dimension, two light yellow spots and a dark yellow spot with R_*f*_ = 0.92, 0.87 and 0.81 were detected. TE(2)/MeOH showed three spots of yellow color, a dark yellow, two light yellow colored spots and a light red spot with R_*f*_ = 0.34, 0.46, 0.59 and 0.91 respectively in 1^st^ dimension. In the 2nd dimension, two blue spots and two yellow spots with R_*f*_ = 0.23, 0.68 and 0.88, 0.91 were visualized. In 1^st^ dimension, TER/MeOH showed a fluorescent blue and three sky blue spots appeared with R_*f*_ = 0.48, 0.84, 0.89, 0.97 and in 2^nd^ dimension fluorescent blue, purple, yellow and sky blue spots with R_*f*_ = 0.79, 0.5, 0.53 and 0.66 were observed. The information regarding the level of hydroxylation and glycosylation was gleaned from 2D-PC. It indicated the presence of phenolic acids, flavanones, flavones, flavanols and isoflavones and flavonoid glycosides. The **Figs 2–4** indicated the 2D-PC and TLC Chromatogram of TE(1), TE(2) and TER/MeOH.

**Fig 5:**
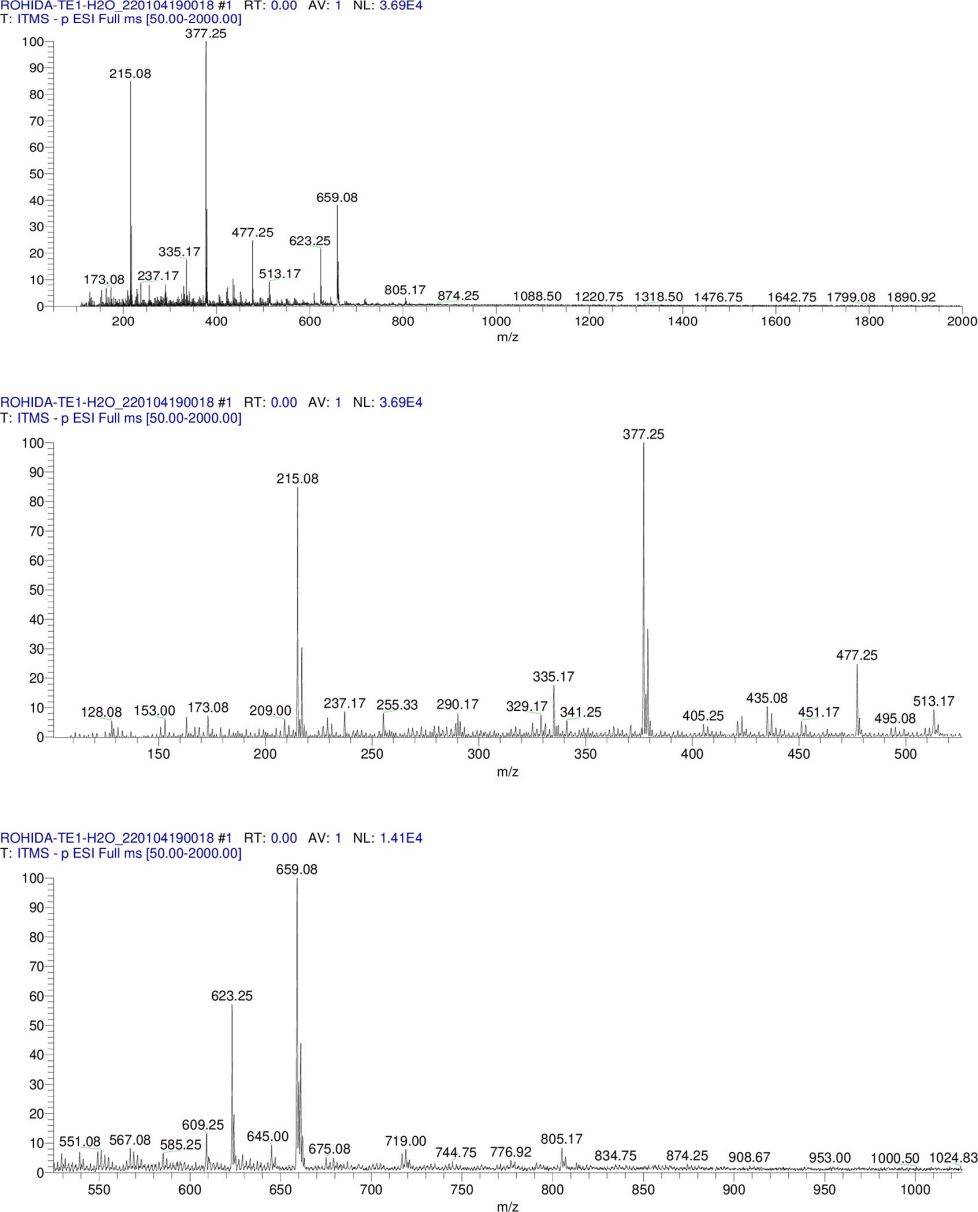
Methanolic extract MS/MS TIC profile of TE(1)/MeOH. Fig 5 showed the TIC profile of TE(1) in MS/MS.

**Fig 6:**
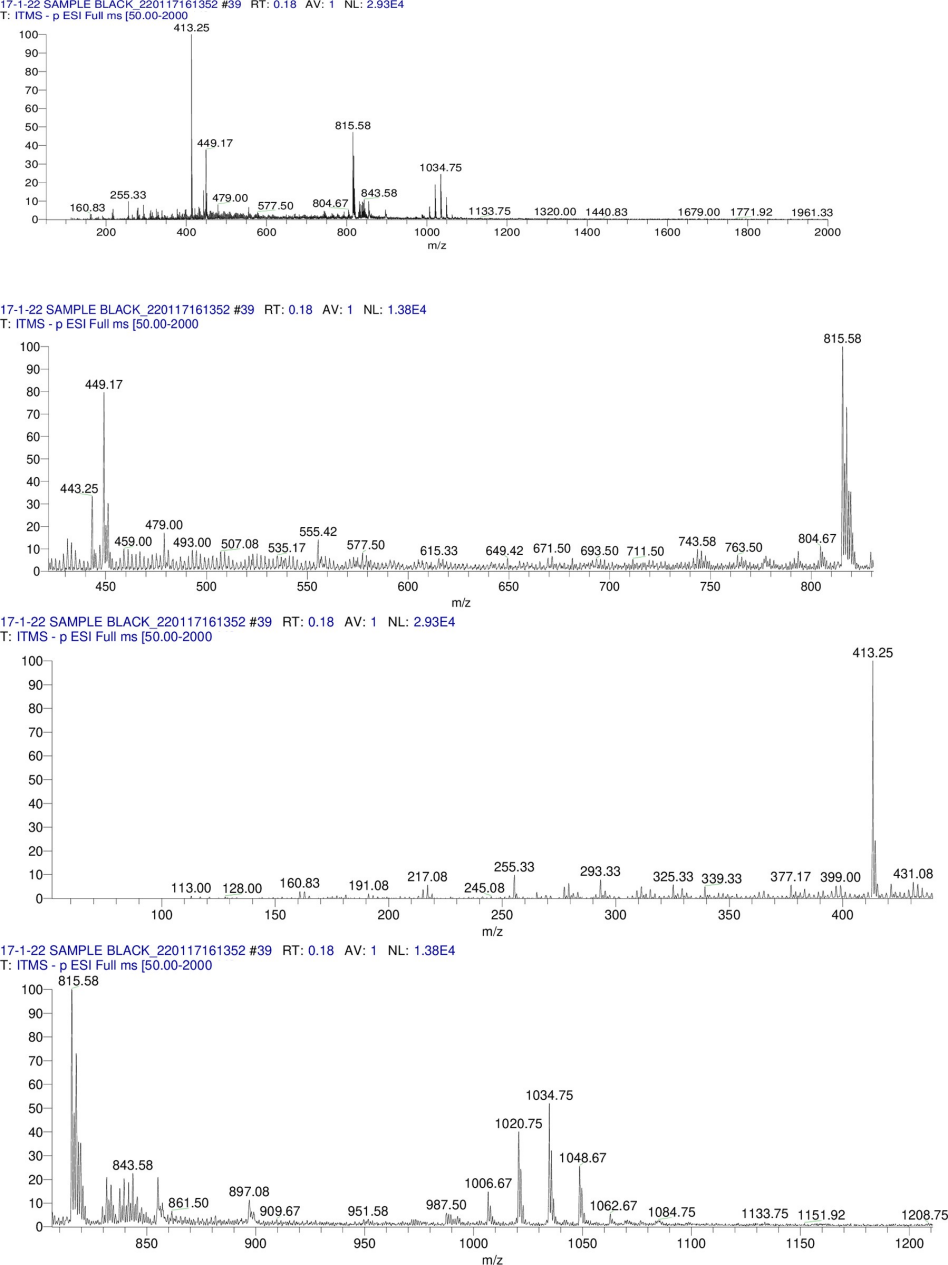
Methanolic extract MS/MS TIC profile of TE(2)/MeOH. Fig 6 showed the TIC profile of TE(2) in MS/MS.

**Fig 7:**
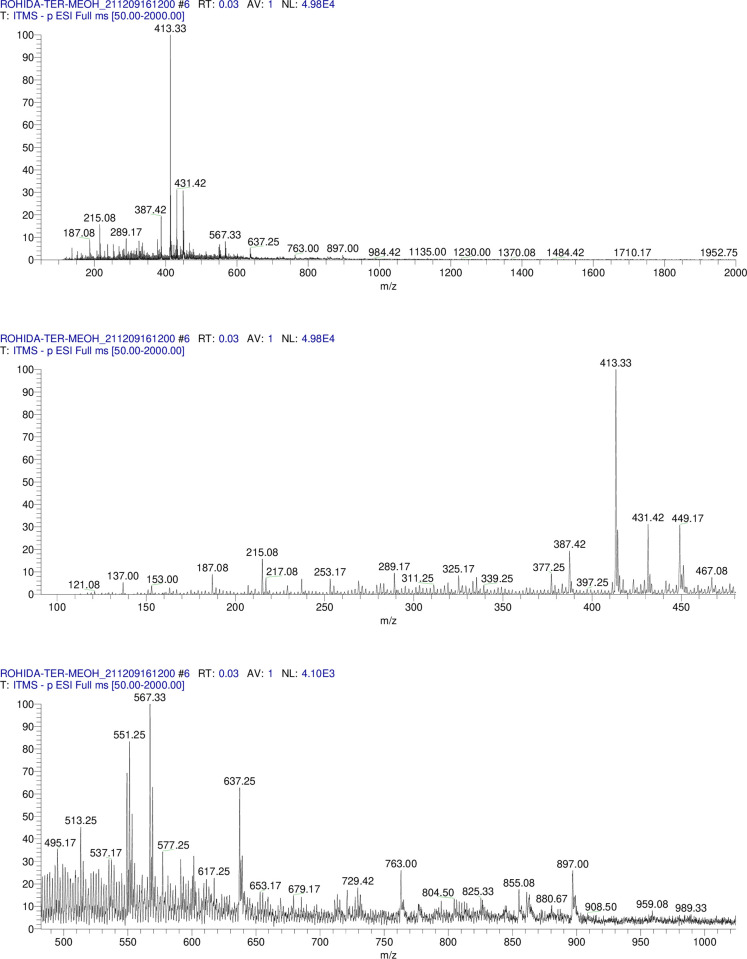
Methanolic extract MS/MS TIC profile of TER/MeOH. Fig 7 showed the TIC profile of TER/MeOH in MS/MS.

Glucose, rhamnose and xylose were detected in TE(1)/MeOH and TER/MeOH but only glucose was detected in TE(2)/MeOH hydrolysate. The R_*f*_ values of these sugars matched with the standard sugars applied on the Co-TLC. The 62 compounds were tentatively identified from TE(1)/MeOH, TE(2)/MeOH and TER/MeOH which are presented in **Tables 2–4** with their respective deprotonated molecular ion and product ions.The TIC profile of TE(1), TE(2) and TER/MeOH in **Figs 5–7.** indicated the precursor ions appeared in MS^1^ at the mass range of 50–2000 *amu*.

**Table 2:** Phytochemical compounds detected and characterized in methanolic extract of *Typha elephantina* TE(1)/MeOH aerial parts.

S. No	Names	[M-H]^-^(*m/z*)	Fragment Ion Peaks (*m/z*)	References
TE (1)-1	Protocatechuic acid (3,4 dihydroxy benzoic acid)	153	137.83, 121.00, 109.00, 95.58, **95.00**, 92.92	[23]
TE (1)-2	*p-*Coumaric acid	163	148.00, 131.00, **119.00**, 105.08, 94.83	[24, 25]
TE (1)-3	Phenyl-2,2,2 trihydroxy	183	169.75,164.33,**146.92**,139.08, 113.67, 112.08	[26]
	Acetate			
TE (1)-4	Acetylene shikimate	197	183.67, 179.00, **161.08**, 153.25, 141.25, 125.75, 111.00, 97.08, 74.92	[27]
TE (1)-5	Caffeic acid .2H_2_O	215	197.00, **179.08**,177.00,161.08, 143.00, 119.00, 101.00, 89.00, 71.00	[25, 27, 28]
TE (1)-6	Dihydrocaffeic acid .2H_2_O	217	199.17, 180.92, **179.08**, 173.08, 160.83, 143.08, 119.08, 97.00, 88.92	[25, 28]
TE (1)-7	4-hydroxy-3 methoxy-4-(3,4,5-trioxotetra hydro furan) butanal,	229	218.17, 214.92, 211.00, 198.83, 197.00, 193.17, 190.75, 185.08, 171.17, 155.08, 151.08, 140.08, 130.08, 126.92, 111.08, 97.50, 96.92, **94.92**, 92.92, 75.50, 74.92.	[29]
	Dehydroascorbic acid derivative			
TE (1)-8	1-*O*-Coumaroyl glycerol	237	219.08, 201.08, 179.00, 163.00, 156.00, 145.00, 138.92, **119.08**, 98.58, 92.92, 83.08	[28]
TE (1)-9	Glucaroyl-4-hydroxy benzoate	329	311.17, 293.00, 275.17, 270.75, 268.83, 242.75, 229.25, 210.92, **209.00**, 201.25, 173.17, 150.67, 136.83, 123.00, 93.00	[23, 30]
TE (1) -10	6-*O*-caffeoyl glucoside .2H_2_O	377	345.08, **341.17**, 313.17, 285.25, 244.92, 215.08, 197.00, 179.08, 161.00, 143.17, 119.00	[25, 28]
TE (1)-11	6-*O*-dihydrocaffeoyl glucoside .2H_2_O	379	347.17, **341.17**, 320.83, 277.17, 262.92, 227.08, 217.08, 199.00, 178.83, 160.67, 131.00	[25, 28]
TE (1)-12	3′-*C*- (4-Propenyl cyclohexyl-1,2 diol) apigenin	423	405.17, **387.17**, 365.33, 345.17, 311.25, 305.92, 298.83, 275.08, 249.08, 225.25, 223.08, 197.00, 160.83, 139.00	[31]
TE (1)-13	3-*O*-Glucopyranosyl isorhamnetin.	477	441.92, 419.92, 388.25, 358.17, 330.08,	[27, 32, 33]
			**315.08**, 286.00, 271.00, 244.00, 221.17, 204.83, 180.00, 151.00	
TE (1)-14	6-*O*-Pentenoyl glucopyranosyl-6-*C*-apigenin,	513	**477.17**, 469.17, 411.17, 379.08, 327.08, 323.00, 297.00, 293.17, 278.83, 233.17, 190.83, 172.33	[34, 35]
	Isovitexin derivative			
TE (1)-15	Quercetin-3-*O*-[*α-*L-rhamnopyranosyl-(1→6)-*β-*D*-*glucopyranoside])	609	591.17, 535.33, 491.25, 447.33, 384.75, 315.08, **301.08**, 271.08, 243.83, 193.00	[27, 32, 33]
	(Rutin)			
TE (1)-16	Isorhamnetin-3-*O*-[*α-*L-rhamnopyranosyl(1→2)glucopyranoside]	623	587.00, 503.25, 458.92, 355.08, **315.08** 300.08, 271.00, 244.00, 189.08	[27, 32, 33]
TE (1)-17	Verbascoside .2H_2_O	659	**623.17**, 599.33, 563.25, 541.25, 473.58, 461.50, 400.08, 396.92, 346.33, 271.17, 249.08	[36, 40]
TE (1)-18	Forsythoside A .2H_2_O+H_2_	661	629.42, **623.08**, 612.83, 561.83, 493.00, 461.17, 434.58, 374.25, 355.17, 311.42, 253.25	[36, 40]

**Table 3:** Phytochemical compounds detected and characterized in methanolic extract of *Typha elephantina* TE(2)/MeOH aerial parts.

S.No	Names of Compounds	[M-H]^-^(*m/z*)	Fragment ion peaks (*m/z*)	References
TE(2)-1	3-Amino-1-methyl piperidine (Alkaloid)	113	98.58, 94.92, 87.67, 85.00, 81.00, 70.00, **69.00**, 59.50, 59.08, 58.00	[37, 38]
TE(2)-2	Piperidine-1-carboxylic acid	128	111.17, 110.00, 84.83, **84.08**, 83.83, 83.08, 56.67, 56.08	[37, 38]
TE(2)-3	3-Amino-5-hydroxy piperidine-1-methane-diol	161	160.00, 145.92, 141.92, 129.83, 128.08, **116.00**, 112.92, 100.00, 97.92, 88.08, 84.08, 73.92, 59.67, 59.08	[37, 38]
TE(2)-4	Ascorbic acid	175	161.83, 159.83, **157.08,** 147.08, 139.33, 129.08, 116.75, 114.83, 111.00, 95.67, 95.08, 84.92, 73.67, 73.00, 71.00	[27]
TE(2)-5	Dihydrocaffeic acid	181	166.00, **163.00**, 161.08, 149.00, 131.00, 119.00, 113.00, 101.08, 96.92, 89.00, 85.08, 71.00, 59.00	[28, 39]
TE(2)-6	Quinic acid	191	176.00, 173.08, 171.00, 155.00, 149.00, 146.92, 127.00, 125.00, 111.00, 101.08, 93.00, **85.00**, 71.00, 59.08	[24, 40]
]TE(2)-7	Galactonic acid.H_2_O	213	195.08, 185.08, **177.08**, 169.17, 158.92, 151.08, 139.25, 129.00, 125.08, 107.00, 98.92, 78.92	[36]
TE(2)-8	Caffeic acid	215	197.00, 183.00, **179.00**, 177.00, 161.00, 153.08, 143.08, 129.00, 118.92, 89.17, 78.83	[25, 28]
TE(2)-9	Dihydrocaffeic acid .2H_2_O	217	199.08, **181.08**, 179.08, 173.00, 147.92, 136.25, 118.92, 95.83, 89.00, 75.00	[28, 39]
TE(2)-10	Dihydrocaffeic acid .2H_2_O+H_2_	219	201.08, **181.08**, 175.00, 157.00, 143.00, 129.00, 119.00, 88.00, 69.00	[28, 39]
TE(2)-11	Pinocembrin (flavanone)	255	**240.08**, 237.17, 227.08, 211.17, 182.92, 151.00, 137.08, 123.17, 94.83, 85.00	[41, 42]
TE(2)-12	Apigenin (flavone)	269	254.08, 251.17, 241.08, **225.25**, 223.17, 213.08, 197.00, 195,08, 183.17, 159.17, 131.00, 113.08, 97.08, 89.00	[28, 43]
TE(2)-13	Prunetin (Isoflavone)	283	**268.00**, 255.08, 240.08, 224.08, 207.17, 183.08, 181.08, 155.08, 136.83, 102.25, 92.92	[42, 43]
TE(2)-14	1-*O*-feruloyl-3-*O*-*p*-coumaroyl glycerol	413	398.17, 369.17, 354.25, 313.08, 293.08, 267.08, 249.08, **235.08**, 219.08, 205.08, 193.08, 177.08, 161.08, 135.00	[28, 44]
TE(2)-15	1,3-*O*-diferuloyl glycerol	443	428.17, 399.42, 369.33, 353.25, 307.33, 293.08, 267.08, 249.08, **235.08,** 217.08, 207.08, 193.08, 175.08, 161.08, 149.08, 134.00	[28, 44]
TE(2)-16	3-Dihydroferuloyl-1-dihydro caffeoyl propane-1,2-diol	449	417.33, **413.25**, 405.50, 387.33, 345.25, 294.83, 281.25, 255.25, 235.08, 203.25, 181.17, 153.00, 137.08	[28]
TE(2)-17	3-[(3-ethynyl) caffeoyl-2-oxo-propyl] ferulic acid	451	433.50, 415.25, **413.25,** 407.33, 391.08, 365.33, 339.25, 319.33, 305.33, 283.25, 255.25, 225.08, 200.17, 173.00, 153.08	[28]
TE(2)-18	3-[1,3-dihydroxy-3-(4-hydroxy-3-methoxy phenyl) propoxy]-2,3-dihydroxy propyl ferulic acid.	479	461.25**, 443.00,** 435.33, 405.33, 369.42, 343.33, 311.25, 281.17, 255.42, 217.58, 201.00, 171.33, 149.08	[28]

**Table 4:** Phytochemical compounds detected and characterized in methanolic extract of *Typha elephantina* TER/MeOH roots.

S.No	Names of Compounds	[M-H]^-^(m/z)	Fragment ions	References
TER-1	2-Hydroxy benzoic acid	137	122.00, 109.08, 93.58, **93.00,** 80.92, 75.00, 64.92	[23]
TER-2	4-Hydroxy acetophenone	151	136.08, 132.92, 130.92, 107.08, **92.92,** 89.00, 78.92, 71.00, 59.00	[23]
TER-3	Protocatechuic acid	153	138.08, 123.08, **109.00**, 107.17, 94.92,	[24]
			92.92, 83.08, 69.00	
TER-4	*p*-Coumaric acid	163	148.00, 135.00, **119.00**, 104.92, 93.08,	[25, 45]
			82.92, 74.92, 59.00	
TER-5	Gallic acid.H_2_O	187	171.92, 169.08, 159.08, 143.08, 141.00, **125.08**, 123.00, 107.08, 97.08, 82.92, 72.92, 56.92	[25, 28, 46, 47]
TER-6	Dihydrofurano coumarin.CH_3_	202	188.33, 187.08, 174.08, 166.00, **158.08**, 145.92, 143.08, 140.08, 126.00, 116.00, 114.75, 94.25, 87.92, 71.17	[48, 49]
TER-7		207	205.33, 192.08, 189.00, 179.00, 177.00, **163.08**, 161.08, 145.08, 135.00, 122.00, 119.08, 109.08, 93.00, 85.08, 71.33	[28]
TER-8	Caffeic acid. 2H_2_O	215	212.92, 197.08, 187.08, **179.00,** 161.00, 153.08, 143.00, 130.92, 119.00, 101.08, 89.00, 71.08	[25, 28]
TER-9	Dihydrocaffeic acid	217	215.08, 202.08, 199.08, 181.08, **179.00**, 173.08, 171.00, 161.00, 143.00, 131.00, 118.92, 101.00, 89.00, 70.92, 65.08	[25, 28, 39]
	.2H_2_O			
TER-10	4-Hydroxy-3 methoxy-4(3,4,5-trioxo-tetrahydro furan) butanal	229	213.08, **211.08**, 201.17, 193.00, 185.08, 167.08, 147.00, 143.17, 119.08, 109.00, 94.92, 83.08, 70.83	[29]
	Dehydroascorbic acid derivative			
TER-11	1*-O*-Coumaroyl glycerol	237	222.08, 205.00, 193.08, 177.08, 163.00, 161.08, 149.00, 145.00, 142.92, **119.00**, 117.00, 97.00, 81.00	[28]
TER-12	Daidzein,	253	251.00, 235.08, **225.08**, 209.08, 197.08, 181.00, 167.08, 157.00, 153.08, 135.08, 123.00, 97.00, 85.08, 74.92	[28]
	Iso flavone			
TER-13	Pinocembrin,	255	253.25, **240.08**, 223.08, 211.08, 195.00, 181.08, 175.00, 167.00, 151.00, 127.00, 108.92, 92.92, 85.17	[41, 42]
	flavanone			
TER-14	Genistein,	269	267.00, 254.08**, 241.08**, 225.08, 210.83, 197.08, 195.08, 173.00, 159.08, 152.92, 150.92, 131.08, 121.00, 97.00, 92.92, 82.92	[51]
	Isoflavone			
TER-15	1-*O*-coumaroyl 2,3 dihydroxy butanoic acid	281	266.08, 263.17, 249.00, **237.08,** 233.08, 219.08, 207.08, 193.17, 182.00, 163.08, 145.08, 123.00, 117.00, 96.83, 87.08	[28]
TER-16	Catechin	289	271.08, 259.08, 247.08, **245.08**, 231.00, 209.08, 205.08, 203.00, 187.00, 179.08, 165.00,137.00, 125.00, 109.00, 97.00, 83.08	[28]
TER-17	Dihydro Quercetin,	303	286.92, 285.17, 267.08, 257.17, **241.08**, 224.83, 217.08, 212.92, 199.08, 185.00, 175.00, 156.92, 141.17, 112.75, 111.08	[52]
	flavanonol			
TER-18	Ampelopsin, Dihydromyricetin,	319	304.08, **301.17,** 287.08, 275.08, 257.17, 239.08, 224.00, 197.00, 177.00, 165.00, 145.00, 125.08, 107.08, 96.92	[53]
	flavanonol			
TER-19	6-*O*-Caffeoyl glucoside .2H_2_O	377	359.25, 345.17, **341.17**, 333.17, 315.25, 297.08, 279.17, 245.08, 221.08, 215.00, 197.00, 179.08, 161.00, 137.08, 113.00	[27, 28]
TER-20	Feruloyl methyl caffeicacid	385	367.17, 349.17, 341.17, 325.25, 305.25, 303.25, 293.17, 268.83, 266.83, 259.17, 231.08, 217.08, 203.08, **190.00**, 179.00, 163.08, 145.00, 139.08, 117.00	[28]
TER-21	Feruloyl methyl dihydrocaffeic acid.	387	369.17, 343.33, 317.33, 305.17, 268.83, 234.92, 203.00, 190.00, 179.08, 163.08, **145.00**, 132.00,119.00	[28]
TER-22	Feruloyl methyl ethenyl caffeate	411	393.25, 367.25, 349.25, 331.25, 287.17, 259.17, 245.08, 217.00, **203.00**, 190.00, 176.00, 151.92, 134.08	[28]
TER-23	1-*O*-feruloyl-3-*O*-*p*-coumaroyl glycerol.	413	398.17, 369.33, 345.33, 327.25, 315.25, 303.33, 273.08, 259.17, 245.08, 235.08, 217.08, 203.08, **190.00,** 177.08, 161.00, 152.00, 135.08	[28]
TER-24	Isovitexin	431	416.33, 413.33, 401.25, 387.25, 373.17, 345.33, 313.17, 305.25, 277.08, 261.08, 241.17, 218.00, 187.17, 167.08, 149.00, **125.00**	[34, 35]
TER-25	6-*O*-Pentenoyl glucopyranosyl-6-*C*-apigenin,	513	495.33, **477.17**, 469.08, 455.42. 433.33, 395.25, 377.08, 349.00, 329.33, 293.08, 283.17, 255.08, 232.92, 190.92, 165.00	[34, 35]
	Isovitexin derivative			
TER-26	Procyanidin B1	577	562.42, 559.25, 541.33, 533.17, 503.33, 485.33, 471.08, 451.17, **425.17**, 407.25, 381.25, 357.08, 331.17, 299.00, 289.08, 244.92, 198.92, 174.92	[54, 55]

### 3.2. Compounds identified from TE (1)/MeOH

The deprotonated molecular ion peak [M-H]^-^ of compound (1) appeared at *m/z* 153 while the actual mass of compound was 154 *a*.*m*.*u*. The product ion peak at *m/z* 138 appeared due to loss of methyl radical. The loss of oxygen molecule from [M-H]^-^ gave a peak at *m/z* 121 corresponding to benzoyl group. The loss of 44 Da corresponding to CO_2_ from [M-H]^-^ gave the fragment ion peak *i*.*e*., deprotonated catechol at *m/z* 109. Further loss of 57 Da and 58 Da from the deprotonated molecular ion peak gave the product ion peak at *m/z* 96 and the base peak at *m/z* 95 respectively. The product ion peak at *m/z* 93 [23] of phenoxide ion upon loss of 60 Da indicated the presence of COOH group in the identified compound. Hence, in the light of above arguments and reported literature [28] the compound (1) was tentatively identified as 3,4-dihydroxy benzoic acid/ protocatechuic acid.

The quasi-molecular ion peak appeared at *m/z* 163 with the actual mass of the compound being 164 *a*.*m*.*u*. The loss of methyl radical from parent molecular ion gave the product ion peak at *m/z* 148. The peak at *m/z* 131 appeared due to loss of oxygen molecule from [M-H]^-^ indicating the presence of two hydroxyl groups in the compound. The base peak at *m/z* 119 [24, 25] appeared on the mass spectrum upon the loss of CO_2_ from [M-H]^-^. The peaks at *m/z* 105 and *m/z* 95 appeared due to significant losses of 58 Da and 68 Da respectively from parent ion. The whole analysis tentatively recognized that compound (2) was *p*-coumaric acid.

The deprotonated precursor ion peak [M-H]^-^ of compound (3)appeared at *m/z* 183 with the actual mass of 184 *a*.*m*.*u*. The loss of 13 Da and 19 Da from [M-H]^-^ gave product ions at *m/z* 170 and *m/z* 164. Base peak at *m/z* 147 [26] via the loss of 36 Da [M-H-2H_2_O]^-^ showed the presence of phenyl acetate derivative. The loss of 44 Dafrom parent ion [M-H-CO_2_]^-^ showed product ion peak at *m/z*139. The peaks at *m/z*114 and *m/z* 112 were assigned for the loss of 69 Da and 71 Da *i*.*e*., [M-H-3OH^·^+H_2_O]^-^ and [M-H-C_3_O_2_H_3_]^-^. The entire fragmentation scheme suggested that compound (3) was Phenyl-2,2,2-Trihydroxyacetate.

The deprotonated molecular ion peak [M-H]^-^ for compound (4) displayed at *m/z* 197 with the actual mass of 198 *a*.*m*.*u*. Which suffered the loss of 13 Dayielding *m/z* 184. The loss of water molecule from molecular ion gave the daughter ion which appeared at *m/z* 179. The base peak at *m/z* 161 was produced via the loss of 36 Da [M-H-2H_2_O]^-^. The daughter ion peak at *m/z* 153 was generated upon the loss of 44 Da indicating the presence of COOH moiety. The fragment ion peaks at *m/z* 141, *m/z* 126, *m/z* 111, *m/z* 97 and *m/z* 75 appeared on MS^2^ due to significant losses of 56 Da, 71 Da, 86 Da, 100 Da and 122 Da from the precursor ion. The base peak at *m/z* 161 [28] was observed on mass spectrum and upon comparison with bibliographic data it matched with the base peak of shikimic acid derivative. All the fragment ions and their losses affirmed that compound (4) was Acetylene shikimate.

The molecular ion peak of compound (5) appeared at *m/z* 215. The actual mass of compound was 216 *a*.*m*.*u*. The loss of 18 Da from [M-H]^-^ gave the daughter ion peak at *m/z* 197. The sequential loss of 36 Da from precursor ion resulted in the base peak at *m/z* 179 corresponding to the caffeic acid [25, 28]. The loss of neutral hydrogen molecule from base peak gave peak at *m/z* 177. An additional loss of 18 Da from the base peak gave the daughter ion peak at *m/z* 161. The loss of 36 Da of two water molecules from base peak gave the fragment ion peak at *m/z* 143. The *m/z* 161 and *m/z* 143 were characteristic fragment ion peaks of caffeoyl moiety [26]. The peak at *m/z* 119 of vinyl 4-hydroxy benzene was obtained upon the loss of 60 Da. The fragment ion peaks at *m/z* 101, *m/z* 89 and *m/z* 71 appeared on the mass spectrum due to the losses of 78 Da, 90 Da and 108 Da from the fragment ion which gave the base peak. From the above arguments, it was proclaimed that compound (5) was the Dihydrate of caffeic acid.

The [M-H]^-^ of the compound (6) appeared at *m/z* 217 with 218 *a*.*m*.*u*. as its actual mass. The peak at *m/z* 199 was observed upon the loss of a water molecule from the precursor ion. The characteristic peak at *m/z* 181 corresponding to dihydrocaffeic acid appeared by the loss of 36 Da. The loss of 2 Da (H_2_) from *m/z* 181 gave the base peak at *m/z* 179 due to deprotonated caffeic acid [25, 28] among them the loss of 6 Da (3H_2_) resulted *m/z* 173. The loss of 18 Da and 36 Da from base peak generated characteristic fragment ions of caffeic acid at *m/z* 161 and *m/z* 143 [27]. The daughter ion peak at *m/z* 119 characteristic of vinyl 4-hydroxy benzene was produced by the loss of 60 Da from *m/z* 179. The peaks at *m/z* 97 and *m/z* 89 were observed due to losses of 82 Da and 90 Da from the fragment ion peak at *m/z* 179. The above discussion signalized that compound (6) was a Dihydrate of dihydrocaffeic acid.

The compound (7) yielded deprotonated molecular ion peak [M-H]^-^ at *m/z* 229 and the actual mass was inferred as 230 *a*.*m*.*u*. The product ion peak appeared at *m/z* 215 by the loss of 14 Da. The loss of 18 Da from [M-H]^-^ gave the daughter ion peak at *m/z* 211. The peak at *m/z* 199 was produced due to removal of 30 Da [M-H-CO+H_2_]^-^ from it the loss of 2 Da resulted in product ion at *m/z* 197. The peak at *m/z* 193 was initiated by the loss of 36 Da (2H_2_O) from the deprotonated molecular ion. The losses of 38 Da (2H_2_O+H_2_) and 44 Da (CO_2_) from the parent ion gave peaks at *m/z* 191 and *m/z* 185. The peaks at *m/z* 211 and *m/z* 191 were the characteristic peaks of dehydroascorbic acid [29] portraying the presence of dehydroascorbic acid. With the loss of 58 Da [M-H-C_3_H_6_O]^-^ the *m/z* 171 was obtained from precursor ion. The peak at *m/z* 155 was produced from [M-H]^-^ due to the loss of 74 Da. From *m/z* 191, an additional loss of 40 Da gave the fragment ion peak at *m/z* 151. The *m/z* 140, *m/z* 130 and *m/z* 127 were produced upon the loss of 89 Da (C_4_H_9_O_2_), 99 Da (C_3_O_4_) and 102 Da (C_4_H_6_O_3_) from the deprotonated molecular ion peak. The loss of 16 Da from the peak at *m/z* 127 gave rise to a fragment ion peak at *m/z* 111. From it (*m/z* 111), the loss of 13 Da, 14 Da, 16 Da and 18 Da generated *m/z* 98, *m/z* 97, *m/z* 95 and *m/z* 93. The last two daughter ion peaks at *m/z* 76 and *m/z* 75 appeared due to the loss of 153 Da (C_7_H_5_O_4_) and 154 Da (C_6_H_2_O_5_) from the precursor ion. The whole fragmentation pattern ascertained that compound (7) was the derivative of dehydroascorbic acid.

The deprotonated precursor ion [M-H]^-^ for compound (8) appeared at *m/z* 237. Its actual mass was inferred as 238 *a*.*m*.*u*. The loss of 18 Da (H_2_O) gave the fragment ion peak at *m/z* 219. The loss of two water molecules from precursor ion gave rise to the fragment ion peak at *m/z* 201. Further, the peak at *m/*z 179 was generated due to removal of 58 Da (C_2_H_2_O_2_) from the parent ion. The prominent peak at *m/z* 163 appeared by the loss of 74 Da (C_3_H_6_O_2_) from *m/z* 237. The fragment ion peaks at *m/z* 156 and *m/z* 145 were produced due to the losses of 81 Da (C_5_H_5_O) and 92 Da (C_3_H_8_O_3_) from the parent ion respectively. The fragment ion peaks at *m/z* 163 and *m/z* 145 were the characteristic peaks of coumaric acid [28]. The peak at *m/z* 139 appeared on the MS^2^ upon the removal of 98 Da (C_4_H_2_O_3_) from *m/z* 237. The base peak at *m/z* 119 which is characteristic of 4-hydroxy vinyl benzene was obtained by the loss of 118 Da (C_4_H_6_O_4_) from parent ion. The peaks at *m/z* 99, *m/z* 93 and *m/z* 83 were produced by the loss of 138 Da (C_4_H_10_O_5_), 145 Da (C_6_H_8_O_4_), 154 Da (C_7_H_6_O_4_) from *m/z* 237 respectively. The peak at *m/z* 93 is the characteristic peak of phenol. Hence, the compound (8) was tentatively identified as 1-*O*-coumaroyl glycerol.

The compound (9) exhibited the deprotonated molecular ion peak [M-H]^-^ at *m/z* 329 with the actual mass of the compound being 330 *a*.*m*.*u*. The fragment ion peak at *m/z* 311 was produced upon the loss of 18 Da (H_2_O) from the precursor ion. The peak at *m/z* 293 was due to the removal of 36 Da (2H_2_O) from *m/z* 329. The loss of 54 Da (3H_2_O) from molecular ion gave the peak at *m/z* 275. The product ion at *m/z* 271 gave second most intense peak which was produced due to 58 Da from parent ion. An additional loss of hydrogen molecule from *m/z* 271 gave the fragment ion peak at *m/z* 269. The daughter ion peak at *m/z* 243 appeared via the loss of 86 Da (C_4_H_4_O+H_2_O) from the precursor ion. The loss of 100 Da (C_4_H_4_O+O_2_) from *m/z* 329 yielded the daughter ion peak at *m/z* 229. The additional loss of 18 Da from it gave peak at *m/z* 211. The base peak at *m/z* 209 [30] was observed due to the loss of 120 Da (C_7_H_4_O_2_) from deprotonated ion peak indicating the presence of glucaric acid. The peak at *m/z* 201 was obtained by the loss of 28 Da from *m/z* 229. The loss of 36 Da and 58 Da from base peak provided peaks at *m/z* 173 and *m/z* 151 respectively. The daughter ion peak at *m/z* 137 was generated by the loss of 192 Da (C_6_H_8_O_7_) from precursor ion. The removal of 14 Da from *m/z* 137 gave the peak at *m/z* 123. The fragment ion peak at *m/z* 93 [23] *i*.*e*., the characteristic peak of phenoxide ion was produced due to loss of 236 Da (C_7_H_8_O_9_) from *m/z* 329. The overall fragmentation outline reveals the presence of glucaric acid with benzoic acid therefore compound (9) was tentatively identified as Glucaroyl-4-hydroxy benzoate.

The molecular ion peak in negative mode for compound (10) appeared at *m/z* 377 with its actual mass of 378 *a*.*m*.*u*. The second most intense fragment ion peak appeared at *m/z* 345 due to the loss of 32 Da (O_2_) from molecular ion peak. The base peak at *m/z* 341 characteristic of caffeoyl hexoside was obtained by the loss of 36 Da from the parent ion. The loss of 28 Da gave daughter ion peak at *m/z* 313 from *m/z* 341. The removal of 28 Da from *m/z* 341 [28] brought out *m/z* 285. The loss of 96 Da from base peak (C_3_H_12_O_3_) provided peak at *m/z* 245. From deprotonated precursor ion peak, the loss of 162 Da C_6_H_10_O_5_ corresponding to glucose from *m/z* 377 yielded the fragment ion peak at *m/z* 215. The removal of 18 Da (H_2_O) from *m/z* 215 initiated the fragment ion peak at *m/z* 197. The daughter ion peak at *m/z* 179 characteristic of caffeoyl moiety was produced from *m/z* 215 upon the loss of 36 Da. The peaks at *m/z* 161, *m/z* 143, *m/z* 119 were generated from *m/z* 179 via the losses of 18 Da, 36 Da and 60 Da. The peaks at *m/z* 161 and *m/z* 143 were the characteristic fragment ions of caffeic acid [25] and the daughter ion peak at *m/z* 119 was the characteristic of 4-hydroxy vinyl benzene. The entire fragmentation scheme and the corresponding losses indicated the presence of caffeic acid with glucose hence the compound (10) was tentatively identified as Dihydrate of 6-*O*-caffeoyl glucoside.

The deprotonated molecular ion peak [M-H]^-^ appeared at *m/z* 379 for compound (11). The actual mass of compound was inferred as 380 *a*.*m*.*u*. The daughter ion peak at *m/z* 347 was produced by the loss of 32 Da. The 38 Da loss [M-H-2H_2_O+H_2_]^-^ provided base peak at *m/z* 341 [28] which was the characteristic peak of caffeoyl-*O*-hexoside. The loss of 20 Da and 64 Da from *m/z* 341 gave product ion peaks at *m/z* 321 and *m/z* 277. From them, the loss of 14 Da gave *m/z* 263. Further, the removal of 36 Da (2H_2_O) from *m/z* 263 justified *m/z* 227. The loss of 162 Da (C_6_H_10_O_5_) from *m/z* 379 generated peak at *m/z* 217 indicating the presence of glucose in the compound. The removal of 18 Da (H_2_O) from *m/z* 217 resulted in the peak at *m/z* 199. The loss of 38 Da from *m/z* 217 provided the daughter ion peak at *m/z* 179 very much characteristic of caffeoyl group. The peaks at *m/z* 161 characteristic of caffeic acid [25] and *m/z* 131 were obtained by the loss of 18 Da and 48 Da from *m/z* 179. From the above discussion, it was ascertained that compound (11) was the Dihydrate of 6-*O*-dihydrocaffeoyl glucoside.

The compound (12) exhibited deprotonated molecular ion peak [M-H]^-^ at *m/z* 423 with actual mass of 424 *a*.*m*.*u*. The peak at *m/z* 405 was obtained by the loss of 18 Da from the precursor ion. The loss of 36 Da from *m/z* 423 yielded the base peak at *m/z* 387. The loss of 58 Da (C_4_H_10_) from *m/z* 423 gave peak at *m/z* 365. An additional removal of 20 Da (H_2_O+H_2_) and 54 Da (C_3_H_2_O) provided peaks at *m/z* 345 and *m/z* 311 respectively. The loss of 117 units (C_7_H_17_O) from *m/z* 423 was the cause of the peak appearing at *m/z* 306. The daughter ion peak at *m/z* 299 appeared due to loss of 124 Da (C_8_H_12_O) from the precursor ion. The fragment ion peak at *m/z* 311 suffered the loss of 36 Da yielding another fragment at *m/z* 275. From base peak, the loss of 138 Da (C_8_H_7_O+OH^·^+H_2_) gave peak at *m/z* 249. The loss of 198 Da (C_11_H_16_O_2_+H_2_O) showed peak at *m/z* 225 from precursor ion which is the characteristic peak of apigenin [31]. From it, the loss of 2 Da gave *m/z* 223. The peak at *m/z* 197 appeared due to the loss of 226 Da (C_13_H_6_O_4_) from *m/z* 423. It indicated the presence of 4-Propenyl Cyclohexane 1,2 diol in the identified compound. The fragment ion peaks at *m/z* 161 and *m/z* 139 were obtained by the loss of 36 Da and 58 Da from *m/z* 197. Overall discussion tentatively characterized the compound (12) as 3′-*C*- 4-Propenyl Cyclohexyl-1,2 diol Apigenin.

The deprotonated [M-H]^-^ ion for compound (13) appeared at *m/z* 477 with the actual mass of compound being 478 *a*.*m*.*u*. It suffered the loss of 35 Da (H_2_O+OH^·^) from parent molecular ion yielding the fragment ion peak at *m/z* 442. The daughter ion peak at *m/z* 420 appeared due to the loss of 57 Da from *m/z* 477. The peaks at *m/z* 388 and *m/z* 358 were obtained with losses of 89 Da (C_3_H_5_O_3_) and 119 Da (C_4_H_7_O_4_) from precursor ion respectively. The peak at *m/z* 330 was formed due to loss of 147 Da (C_5_H_7_O_5_) from *m/z* 477. The loss of 15 Da from *m/z* 330 gave the base peak at *m/z* 315 which indicated the presence of isorhamnetin [27, 32, 33]. From the base peak, additional daughter ion peaks at *m/z* 286, *m/z* 271, *m/z* 244, *m/z* 221 and *m/z* 151 were obtained by loss of 29 Da (CHO), 44 Da (CO_2_), 71 Da (C_3_H_3_O_2_), 94 Da (C_5_H_2_O_2_) and 164 Da (C_9_H_8_O_3_). The daughter ion peaks at *m/z* 151 and *m/z* 271 were the characteristic peaks of flavonoids. The peak at *m/z* 151 appeared due to RDA cleavage. The loss of 16 Da from *m/z* 221 gave the fragment ion peak at *m/z* 205. An additional loss of 25 Da from it gave the fragment ion peak at *m/z* 180. The above discussion led to the tentative proposal of compound (13) as 3-*O*-glucopyranosyl isorhamnetin.

The compound (14) displayed deprotonated molecular ion peak [M-H]^-^at *m/z* 513. The actual mass of the compound was inferred as 514 *a*.*m*.*u*. The loss of 36 Da (2H_2_O) from precursor ion gave the fragment ion peak at *m/z* 477. The loss of 44 Da (CO_2_) from the parent ion gave the peak at *m/z* 469. The loss of 102 Da (C_5_H_10_O_2_) from *m/z* 513 provided the fragment peak at *m/z* 411. The peak at *m/z* 379 resulted due to loss of 134 Da (C_8_H_6_O_2_) from parent ion. The peak at *m/z* 327 was obtained by the removal of 186 Da (C_8_H_9_O_4_+OH^·^) from *m/z* 513. From it, the loss of 4 Da (2H_2_) gave peak at *m/z* 323. The peak at *m/z* 298 appeared due to the removal of 216 Da (C_10_H_16_O_5_) from parent ion. From *m/z* 298, the loss of 4 Da (2H_2_) gave peak at *m/z* 293. The peaks at *m/z* 279, *m/z* 233, *m/z* 191 and *m/z* 172 were generated by the losses of 234 Da (C_10_H_18_O_6_), 280 Da (C_13_H_12_O_7_), 322 Da (C_15_H_14_O_8_) and 341 Da (C_16_H_21_O_8_) from *m/z* 513. The base peak at *m/z* 477 matched with reported literature [34, 35] which provided evidence for the presence of Isovitexin. The entire discussion prompted the presence of Isovitexin derivative. So, the compound (14) was identified as isovitexin-4-*O*-glucosyl pentanoate.

The deprotonated molecular ion peak [M-H]^-^ for compound (15) appeared at *m/z* 609 with actual mass of 610 *a*.*m*.*u*. The loss of H_2_O from *m/z* 609 gave fragment peak at *m/z* 591. The peak at *m/z* 535 appeared due to loss of 74 Da (C_3_H_6_O2) from *m/z* 609. The peak at *m/z* 491 was obtained due to the loss of 118 Da (C_5_H_10_O_3_) from precursor ion. The loss of 162 Da from parent molecular ion indicated the presence of hexose moiety (C_6_H_10_O_5_) and gave peak at *m/z* 447. The peak at *m/z* 385 and *m/z* 315 were obtained upon the loss of 224 Da (C_8_H_16_O_7_) and 294 Da (C_11_H_18_O_9_) from *m/z* 609. The peak at *m/z* 315 prompted the presence of flavonoid aglycone. The base peak at *m/z* 301 was observed upon the loss of 308 Da (C_6_H_10_O_4_+C_6_H_10_O_5_) from the parent ion. The loss of 162 Da indicated the presence of glucose and 146 Da loss showed the presence of rhamnose. The base peak at *m/z* 301 showed the presence of Quercetin. As literature reported [27, 32, 33] the loss of 30 Da, 57 Da and 108 Da from the aglycone having *m/z* 301 gave peaks at *m/z* 271, *m/z* 244 and *m/z* 193. The peak at *m/z* 271 is very much characteristic of flavonoid. In the light of all the arguments stated above, compound (15) was tentatively identified as Rutin.

The deprotonated molecular ion [M-H]^-^ peak of compound (16) appeared at *m/z* 623 with an actual mass of 624 *a*.*m*.*u*. The loss of 36 Da (2H_2_O) from precursor ion yielded fragment ion peak at *m/z* 587. The losses of 84 Da and 128 Da from *m/z* 587 gave the fragment ion peaks at *m/z* 503 and *m/z* 459. From *m/z* 459, the removal of 103 Da (C_4_O_3_H_7_) gave peak at *m/z* 355. The loss of 308 Da (C_6_H_10_O_4_+C_6_H_10_O_5_) from *m/z* 623 produced fragment peak at *m/z* 315. The loss of 146 Da indicated the presence of rhamnose and the loss of 162 units showed the presence of glucose while *m/z* 315 represented the aglycone moiety of flavonoid after looking through the literature [27, 32, 33] which was affirmed as isorhamnetin. The peaks at *m/z* 300, *m/z* 271, *m/z* 244 and *m/z* 189 were generated from *m/z* 315 upon the loss of 15 Da, 44 Da, 71 Da and 126 Da. The peak at *m/z* 271 is the characteristic peak of flavonoid. The compound (16) was tentatively identified as isorhamnetin-3-*O*-rhamnosyl(1→2) glucopyranoside upon detailed discussion.

The compound (17) exhibited the deprotonated molecular ion [M-H]^-^ at *m/z* 659. The actual mass of the compound was 660 *a*.*m*.*u*. The daughter ion peak at *m/z* 623 was observed due to the loss of 36 Da (2H_2_O) from the precursor ion. The peaks at *m/z* 599 and *m/z* 563 resulted upon the loss of 24 Da and 60 Da from *m/z* 623. The peaks at *m/z* 541 and *m/z* 474 were obtained by the removal of 82 Da (C_4_H_2_O_2_) and 147+2 Da (C_6_H_11_O_4_+H_2_) from *m/z* 623. The loss of 147 indicated the presence of deoxyhexose sugar. The loss of 161 Da (C_6_H_9_O_5_) and 147+76 Da (C_6_H_11_O_4_+C_3_H_8_O_2_) from *m/z* 623 generated fragment ion peaks at *m/z* 462 and *m/z* 400. From it, the loss of 3 Da gave daughter ion at *m/z* 397. The peaks at *m/z* 346, *m/z* 271 and *m/z* 249 were obtained by the loss of (C_12_H_16_O_6_+OH^·^+2H_2_), (C_10_H_8_O_4_+C_6_H_8_O_5_)and (C_17_H_20_O_9_+3H_2_) from *m/z* 623. The fragmentation pattern, presence of rhamnose sugar and the base peak at *m/z* 623 indicated the presence of verbascoside [36, 40]. Hence, the compound (17) was tentatively identified as Dihydrate of verbascoside.

The compound (18) displayed the deprotonated molecular ion at *m/z* 661 with an actual mass of 662 *a*.*m*.*u*. It suffered the loss of 32 Da yielding *m/z* 629. The loss of 38 Da from precursor ion yielded base peak at *m/z* 623. The peaks at *m/z* 613, *m/z* 562, *m/z* 493 and *m/z* 461 resulted by the sequential loss of 10 Da (5H_2_), 61 Da (C_2_H_5_O_2_),130 Da (C_5_H_6_O_4_) and 162 Da(C_6_H_10_O_5_) from *m/z* 623. The loss of 162 units indicated the presence of hexose moiety. From *m/z* 461, the loss of 26 Da (C_2_H_2_) and 106 Da (C_6_H_2_O_2_) the peaks at *m/z* 435 and *m/z* 355 were justified. The loss of 61 Da (C_2_H_5_O_2_) from *m/z* 435 gave the peak at *m/z* 374. The fragment ion peaks at *m/z* 311 and *m/z* 253 were obtained by the loss of 242+72 Da (C_11_H_14_O_6_+C_3_H_4_O_2_) and 370 Da (C_17_H_22_O_9_) from *m/z* 623 respectively. Looking at the fragmentation pathway, the base peak at *m/z* 623 [36, 40] and the presence of sugar moiety the compound (18) was tentatively identified as Dihydrate of dihydroforsythoside A.

### 3.3. Compounds identified from TE(2)/MeOH

ion [M-H]^-^ appeared at *m/z* 113. The actual mass of the compound (1) was inferred as 114 *a*.*m*.*u*. The precursor ion yielded product ion at *m/z* 99 with the loss of 14 Da [M-H-CH_2_]^-^. The peak at *m/z* 95 was obtained by the loss of 18 Da [M-H-CH_4_+H_2_]^-^and *m/z* 88, *m/z* 85 and *m/z* 81 were generated with the loss of 25 Da [M-H-C_2_H^·^], 28 Da [M-H-C_2_H_4_]^-^and 32 Da [M-H-CH_2_NH_2_+H_2_]^-^. The peaks at *m/z* 70, *m/z* 69 and *m/z* 60 resulted by the loss of 43 Da [M-H-C_2_H_5_N]^-^, 44 Da [M-H-C_3_H_8_]^-^ and 53 Da [M-H-C_4_H_5_^·^]^-^. The peaks at *m/z* 59 and *m/z* 58 were produced by the loss of 54 Da [M-H-C_4_H_6_]^-^and 55 Da [M-H-C_4_H_7_^·^]^-^. The *m/z* 113 and *m/z* 70 were the characteristics peaks of piperidine [37, 38]. From fragmentation scheme, it was decided that compound (1) was identified as 3 -Amino-1-methyl piperidine.

The deprotonated molecular ion [M-H]^-^ appeared at *m/z* 128 the actual mass of compound (2) was 129 *a*.*m*.*u*. The precursor ion suffered the loss of 17 Da [M-H-OH^·^]^-^ yielding *m/z* 111. The *m/z* 110, *m/z* 85 and *m/z* 84 were generated with the loss of 18 Da [M-H-H_2_O]^-^, 43 Da [M-H-C_3_H_7_]^-^ and 44 Da [M-H-CO_2_]^-^. The *m/z* 83, *m/z* 57 and *m/z* 56 resulted by the loss of 45 Da [M-H-COOH]^-^, 71 Da [M-H-C_2_HNO_2_]^-^ and 72 Da [M-H-C_2_H_2_NO_2_]^-^The *m/z* 84 and *m/z* 56 were the characteristics peaks of piperidine as stated in literature [37, 38]. From above arguments, the compound (2) was tentatively considered as Piperidine-1-carboxylic acid.

The deprotonated molecular ion [M-H]^-^ appeared at *m/z* 161. The actual mass of compound (3) was 162 *a*.*m*.*u*. From parent ion the loss of 15 Da [M-H-CH_3_^·^]^-^ brought out *m/z* 146. The *m/z* 142, *m/z* 130 and *m/z* 128 were generated with the loss of 19 Da [M-H-H_2_O+H^·^]^-^, 31 Da [M-H-OCH_3_^·^]^-^ and 33 Da [M-H-NH_2_OH]^-^. The *m/z* 116, *m/z* 113, *m/z* 100 and *m/z* 98 were generated with the loss of 45 Da [M-H-COOH]^-^, 48 Da [M-H-COOH+H_2_+H^·^]^-^, 61 Da [M-H-CH_3_NO_2_]^-^ and 63 Da [M-H-CH_3_NO_2_+H_2_]^-^. The *m/z* 88, *m/z* 84, *m/z* 74, *m/z* 60 and *m/z* 59 resulted by the loss of 73 Da [M-H-C_3_H_7_NO]^-^,77 Da [M-H-C_2_H_7_NO_2_]^-^, 87 Da [M-H-C_3_H_5_NO_2_]^-^,101 Da [M-H-C_5_H_11_NO]^-^ and 102 Da [M-H-C_3_H_6_N_2_O_2_]^-^. The whole fragmentation with supportive evidence [37, 38] suggested that compound (3) was considered as 3-Amino-5-hydroxy piperidine-1-methane-diol.

The deprotonated ion [M-H]^-^ for compound (4) displayed at *m/z* 175 with the actual mass of the compound being 175 *a*.*m*.*u*. From parent ion the loss of 13 Da gave *m/z* 164. Further *m/z* 162 was obtained due to loss of 2 Da from *m/z* 164. The *m/z* 157, *m/z* 147, *m/z* 139 and *m/z* 129 appeared due to the loss of 18 Da [M-H-H_2_O]^-^, 28 Da [M-H-CO]^-^, 36 Da [M-H-2H_2_O]^-^and 46 Da [M-H-CO_2_+H_2_]^-^ respectively. From parent ion the loss of 58 Da [M-H-2CO+H_2_]^-^, 60 Da [M-H-C_2_H_4_O_2_]^-^ and 64 Da [M-H-2O_2_]^-^ generated *m/z* 117, *m/z* 115 and *m/z* 111. From *m/z* 111 the loss of 40 Da and 15 Da gave *m/z* 71 and *m/z* 96. The loss of hydrogen radical from m/z 96 gave *m/z* 95. The loss of 90 Da [M-H-C_3_H_6_O_3_]^-^ and 101 Da [M-H-C_4_H_5_O_3_]^-^ brought out *m/z* 85 and *m/z* 74 upon additional loss of 1 Da gave *m/z* 73. The deprotonated ion and base peak were the characteristic peaks of ascorbic acid [27]. So, the compound (4) was tentatively assigned as Ascorbic acid.

Compound (5) exhibited deprotonated ion [M-H]^-^ at *m/z* 181 with the actual mass of the compound being 182 *a*.*m*.*u*. Precursor ionyielded *m/z* 166 with loss of methyl radical [M-H-CH_3_^.^]. The *m/z* 163 base peak appeared due to the loss of 18 Da water molecule from precursor ion [M-H-H_2_O]^-^.The *m/z* 161 was obtained by the loss of 20 Da [M-H-H_2_O+H_2_]^-^.By the loss of oxygen, *m/z* 149 resulted from parent ion [M-H-O_2_]^-^. The *m/z* 131 was produced upon the loss of 50 Da neutral carbon dioxide and 3 hydrogen molecules [M-H-CO_2_+3H_2_]^-^ from deprotonated ion the loss of 62 Da gave *m/z* 119 [M-H-CO_2_+H_2_O]^-^from it the loss of neutral 3 hydrogen molecule brought out *m/z* 113. *m/z*101, *m/z* 97 and *m/z* 89 were obtained with the loss of 80 Da [M-H-CO_2_+2OH^·^+H_2_]^-^, 84 Da [M-H-C_4_H_4_O_2_]^-^ and 92 Da [M-H-C_2_H_4_O_2_+O_2_]^-^ regarding literature [28, 39] the *m/z* 181 indicated the presence of dihydrocaffeic acid and *m/z* 163, *m/z* 161 were the charachteristic fragment ions of dihydrocaffeic acid. The compound (5) was considered as dihydrocaffeic acid.

The compound (6) showed deprotonated molecular ion at *m/z* 191 and the actual mass of the compound was 192 *a*.*m*.*u*. [M-H]^-^ suffered the loss of methyl radical yielded fragment ion *m/z* 176. The *m/z* 173 was obtained by the loss of water molecule [M-H-H_2_O] the loss of 2 Da from it gave *m/z* 171 the loss of two water molecules from precursor ion [M-H-2H_2_O]^-^ gave *m/z* 155 the 42 Da [M-H-C_2_H_2_O]^-^ loss resulted *m/z* 149 the neutral loss of 44 Da [M-H-CO_2_]^-^ showed product ion at *m/z* 147 an additional loss of two oxygen molecules 64 Da [M-H-2O_2_]^-^ generated regarding literature *m/z* 127 was the characteristic peak of quinic acid [40] from *m/z* 127 the loss of 2 Da (H_2_) hydrogen molecule gave *m/z* 125 further from deprotonated ion the loss of two oxygen molecule and one oxygen radical 80 Da [M-H-2O_2_+O^.^]^-^resulted *m/z* 111 from it the loss of 10 Da (5H_2_) gave *m/z* 101 from precursor ion the loss of 98 Da [M-H-3H_2_O+CO_2_]^-^, 106 Da [M-H-C_2_H_2_O_3_+O_2_]^-^, 120 Da [M-H-C_4_H_7_O_3_+OH^·^]^-^ and 132 Da [M-H-C_5_H_8_O_4_]^-^ resulted *m/z* 93 (catechol), *m/z* 85 (cyclopentanol), *m/z* 71 and *m/z* 59. According to literature [24] the base peak *m/z* 85 indicated the presence of quinic acid. From the entire fragmentation pattern and *m/z* 127, *m/z* 85 and *m/z* 111 were the characteristic of quinic acid the compound (6) was assigned as Quinic acid.

The deprotonated ion appeared at *m/z* 213 for compound (7). The actual mass of the compound was 214 *a*.*m*.*u*. [M-H]^-^ suffered the loss of 18 Da [M-H-H_2_O]^-^ yielded *m/z* 195 from it the loss of 10 Da (5H_2_) gave *m/z* 185 from *m/z* 195 the loss of 18 Da (H_2_O), 26 Da (C_2_H_2_), and 36 Da (2H_2_O) resulted *m/z* 177, *m/z* 169 and *m/z* 159. Further in fragmentation scheme the *m/z* 151, *m/z* 139 and *m/z* 129 were produced from *m/z* 195 by the loss of 44 Da (CO_2_), 56 Da (3H_2_O+H_2_) and 66 Da (COOH+OH^·^+2H_2_). The *m/z* 129 upon the loss of 4 Da (2H_2_) gave *m/z* 125 and from *m/z* 195 the product ions *m/z* 107, *m/z* 99 and *m/z* 79 were generated by the loss of 88 Da (C_3_H_4_O_3_), 96 Da (C_2_H_8_O_4_) and 116 Da (CH_8_O_6_). The base peak *m/z* 177, and *m/z*195, *m/z* 159 and *m/z* 129 were the characteristics of galactonic acid [36]. So, from whole fragmentation pattern and literature information the compound (7) was identified as monohydrate of galactonic acid.

The quasi-molecular ion displayed at *m/z* 215 and the actual mass of the compound (8) was 216 *a*.*m*.*u*. The loss of 18 Da [M-H-H_2_O]^-^, 32 Da [M-H-O_2_]^-^and 36 Da [M-H-2H_2_O]^-^resulted *m/z* 197, *m/z* 183 and base peak *m/z* 179. Further losses were carried out from base peak *m/z* 179. From it the loss of 2 Da (H_2_), 18 Da (H_2_O), 26 Da (C_2_H_2_) and 36 Da (2H_2_O) brought out *m/z* 177, *m/z* 161, *m/z* 153 and *m/z* 143. The *m/z* 129, *m/z* 119, *m/z* 89 and *m/z* 79 were obtained from *m/z* 179 with the loss of 50 Da (H_2_O+CH_2_O+H_2_), a 60 Da (CO_3_), 90 Da (C_2_H_2_O_2_+O_2_) and 100 Da (C_4_H_4_O_3_) regarding literature [25] *m/z* 179 was the characteristic of caffeic acid and *m/z* 161 and *m/z* 143 were the characteristic fragment ions of caffeic acid [28]. So, from above entire discussion it was suggested that compound (8) was identified as dihydrate of caffeic acid.

The compound (9) displayed deprotonated molecular ion [M-H]^-^ at *m/z* 217 with the actual mass of the compound being 218 *a*.*m*.*u*. [M-H]^-^ suffered the loss of 18 Da [M-H-H_2_O]^-^ and 36 Da [M-H-2H_2_O]^-^ yielded *m/z* 199 and base peak *m/z* 181 further losses were done from base peak *m/z* 181. From it the loss of 2 Da (H_2_), 8 Da (4H_2_), 33 Da and 45 Da (COOH) brought out *m/z* 179, *m/z* 173, *m/z* 148 and *m/z* 136 further from *m/z* 181 the fragment ions *m/z* 119, *m/z* 97, *m/z* 89 and *m/z* 75 were generated with the loss of 62 Da (CO_2_+OH^·^), 85 Da (C_4_H_5_O_2_), 92 Da (COOH+CH_2_O+OH^·^) and 106 Da (C_3_H_6_O_4_). The *m/z* 181 base peak was the characteristic of dihydrocaffeic acid as stated in literature [28, 39]. From above fragmentation pattern and literature the compound (9) was considered as dihydrate of dihydrocaffeic acid.

The deprotonated ion [M-H]^-^ for compound (10) was obtained at *m/z* 219 and the actual mass of the compound was 220 *a*.*m*.*u*. [M-H]^-^ yielded *m/z* 201, base peak *m/z* 181 by the loss of 18 Da [M-H-H_2_O]^-^and 38 Da [2H_2_O+H_2_] further losses were carried out from base peak *m/z* 181 from it *m/z* 175, *m/z* 157, *m/z* 143 and *m/z* 129 were generated by the loss of 6 Da (3H_2_), 24 Da (C_2_), 38 Da(2H_2_O+H_2_) and 52 Da (CO_2_+4H_2_) and *m/z* 119, *m/z* 88 and *m/z* 69 were resulted with the loss of 62 Da (COOH+OH^·^), 93 Da (C_2_O_4_H_5_) and 112 Da (C_5_H_4_O_3_). The *m/z* 181 was the characteristic of dihydrocaffeic acid according to literature [28, 39]. The compound (10) was identified as dihydrate of dihydrocaffeic acid.

For compound (11) deprotonated molecular ion [M-H]^-^ appeared at *m/z* 255 with actual mass of 256 *a*.*m*.*u*. [M-H]^-^with the loss of 15 Da [M-H-CH_3_]^-^ gave fragment ion at *m/z* 240 was a base peak. The parent ion generated *m/z* 237, *m/z* 227, *m/z* 211 and *m/z* 183 by the loss of 18 Da [M-H-H_2_O]^-^, 28 Da [M-H-CO]^-^, 44 Da [M-H-CO_2_]^-^ and 72 Da [M-H-CO_2_+CO]^-^.The *m/z* 151, *m/z* 137, *m/z* 123, *m/z* 95 and *m/z* 85 were produced from *m/z* 255 with loss of 104 Da [M-H-C_8_H_8_]^-^, 118 Da [M-H-C_8_H_6_O]^-^, 132 Da [M-H-C_9_H_8_O]^-^,160 Da [M-H-C_10_H_8_O_2_]^-^ and 170 Da [M-H-C_11_H_6_O_2_]^-^. Regarding literature [41, 42] *m/z* 255, *m/z* 183 and *m/z* 151 were the characteristics of pinocembrin. So, from fragmentation and literature the compound (11) was identified as Pinocembrin.

The deprotonated molecular ion appeared at *m/z* 269 for compound (12). The actual mass of the compound was 270 *a*.*m*.*u*. [M-H]^-^with the loss of 15 Da methyl radical [M-H-CH_3_]^-^ gave *m/z* 254. The loss of water molecule from parent ion [M-H-H_2_O]^-^ resulted in *m/z* 251. *m/z* 241 was obtained by the loss of 28 Da [M-H-CO]^-^. The*m/z* 225 was produced by the loss of 44 Da [M-H-CO_2_]^-^carbon dioxide. The *m/z* 223, *m/z* 213, *m/z* 197 were generated by the loss of 46 Da [M-H-CO_2_+H_2_]^-^_,_ 56 Da loss [M-H-C_3_H_4_O]^-^and 72 Da [M-H-C_4_H_8_O]^-^. From *m/z* 197 the loss of 2 Da gave *m/z* 195. The *m/z* 183, *m/z* 159, *m/z* 131, *m/z* 113 and *m/z* 97 were obtained upon the loss of 86 Da [M-H-C_4_H_6_O_2_]^-^, 110 Da [M-H-C_6_H_6_O_2_]^-^, 138 Da [M-H-C_7_H_6_O_3_]^-^, 156 Da [M-H-C_7_H_8_O_4_]^-^ and 172 Da [M-H-C_10_H_4_O_3_]^-^. From *m/z* 113 the loss of 24 Da gave *m/z* 89. Regarding literature [28, 43] the deprotonated molecular ion, base peak *m/z* 225 and fragment ions *m/z* 241 and *m/z* 197 were the characteristics of Apigenin. So, from entire fragmentation pattern and literature information the compound (12) was assigned as Apigenin.

For compound (13) deprotonated ion [M-H]^-^ appeared at *m/z* 283. The actual mass of the compound was 284 *a*.*m*.*u*. [M-H]^-^ with the loss of 15 Da methyl radical gave peak at *m/z* 268. The *m/z* 255, *m/z* 240 and *m/z* 224 were resulted by the loss of 28 Da [M-H-CO]^-^, 43 Da [M-H-CO+CH_3_]^-^, 59 Da [M-H-CO_2_+CH_3_]^-^. From deprotonated ion *m/z* 207 and *m/z* 183 were brought out by the loss of 76 Da [M-H-C_3_H_8_O_2_]^-^ and 100 Da [M-H-C_5_H_8_O_2_]^-^.*m/z* 183 upon the loss of 2 Da hydrogen molecule resulted *m/z* 181. *m/z* 155, *m/z* 138, *m/z* 102 and *m/z* 93 were generated from parent ion by the loss of 128 Da [M-H-C_9_H_4_O]^-^,146 Da [M-H-C_9_H_6_O_2_]^-^, 181 Da [M-H-C_9_H_9_O_4_]^-^and 190 Da [M-H-C_10_H_6_O_4_]^-^. Regarding literature [42, 43] 20 the *m/z* 283 and *m/z* 268 were the characteristics of prunetin. So, from fragmentation pattern and literature evidence the compound (13) was identified as Prunetin.

The deprotonated ion [M-H]^-^ appeared at *m/z* 413 for compound (14) with actual mass of 414 *a*.*m*.*u*. The fragment ions *m/z* 398, *m/z* 369, *m/z* 354 and *m/z* 313 were generated from deprotonated ion with the loss of 15 Da [M-H-CH_3_]^-^, 44 Da[M-H-CO_2_]^-^,59 Da [M-H-C_2_H_3_O_2_]^-^ and 100 Da [M-H-C_5_H_8_O_2_]^-^. The *m/z* 293, *m/z* 267 and *m/z* 249 were obtained by the loss of 120 Da [M-H-C_2_H_4_O_2_]^-^,146 Da [M-H-C_9_H_6_O_2_]^-^and 165 Da [M-H-_9_H_9_O_3_]^-^.Base peak *m/z* 235 appearedfrom deprotonated ion with the loss of [M-H-C_10_H_10_O_3_]^-^. From base peak *m/z* 219, *m/z* 205 and *m/z* 193 were obtained by the loss of 16 Da [M-H-O^.^]^-^ oxygen radical, 30 Da [M-H-CH_2_O]^-^and 42 Da [M-H-C_2_H_2_O]^-^. From *m/z* 193 the loss of 16 Da oxygen radical gave *m/z* 177 from it the loss of 16 Da gave *m/z* 161 and from base peak *m/z* 235 the loss of 100 Da (C_4_H_4_O_3_) brought *m/z* 135. Regarding literature [28, 44] the *m/z* 413 and base peak 235 was the characteristic of 1-*O*-feruloyl-3-*O*-*p*-coumaroyl glycerol from fragmentation scheme and significant losses the compound (14) was considered as of 1-*O*-feruloyl-3-*O*-*p*-coumaroyl glycerol.

The molecular ion [M-H]^-^ peak appeared at *m/z* 443. The actual mass of the compound (15) was 444 *a*.*m*.*u*. The peak at *m/z* 428 was obtained by the loss of 15 Da (CH_3_^·^). From [M-H]^*-*^ the loss 44 Da (CO_2_) from [M-H]^-^ gave peak at *m/z* 399. The *m/z* 369 resulted by the loss of 30 Da (CH_2_O) from *m/z* 399. The loss of 16 Da (O^·^) from *m/z* 369 gave peak at *m/z* 353. From *m/z* 353 the loss of 46 Da (CH_2_O_2_) gave peak at *m/z* 307. The peak at *m/z* 293 appeared by the loss of 150 Da (C_9_H_10_O_2_)from molecular ion peak. The *m/z* 267 was obtained by the loss of 176 Da (C_10_H_8_O_3_) from *m/z* 443. The *m/z* 267 with the loss of 18 Da H_2_O gave peak at *m/z* 249. The loss of 208 Da (C_11_H_12_O_4_) from *m/z* 443 gave base peak at *m/z* 235. The loss of 18 Da H_2_O from *m/z* 235 gave peak at *m/z* 217. The peak *m/z* 207 appeared by the loss of 28 Da (CO) from base peak. The [M-H]^-^ with the loss of 250 Da (C_13_H_14_O_5_) gave peak at *m/z* 193. The loss of 268 Da (C_13_H_16_O_6_) from *m/z* 443 gave peak at *m/z* 175. The *m/z* 161 appeared by the loss of 282 Da (C_14_H_18_O_6_)from [M-H]^-^. The loss of 294 Da (C_14_H_14_O_7_) from [M-H]^-^ gave peak at *m/z* 149. The loss of 15 Da (CH_3_^·^) from *m/z* 149 gave peak at *m/z* 134. from overall fragmentation and literature [27, 43] the compound (15) was assigned as 1,3-*O-*diferuloylglycerol.

The compound (16) deprotonated molecular [M-H]^-^ ion appeared at *m/z* 449 with actual mass of 450 *a*.*m*.*u*. [M-H]^-^ yielded *m/z* 417 with the loss of 32 Da [M-H-O_2_]^-^.From *m/z* 449 the loss of 36 Da [M-H-H_2_O]^-^ gave *m/z* 413. *m/z* 406, *m/z* 387, *m/z* 345 and *m/z* 295 were generated by the loss of 43 Da [M-H-CO+CH_3_]^-^, 62 Da [M-H-C_2_H_2_O_2_+2H_2_]^-^, 104 Da [M-H-C_5_H_6_O_2_+3H_2_]^-^, and 154 Da [M-H-C_9_H_14_O_2_]^-^. The *m/z* 281, *m/z*255, *m/z* 235 and *m/z* 203 were obtained with the loss of 168 Da [M-H-C_9_H_12_O_3_]^-^, 194 Da [M-H-C_10_H_10_O_4_]^-^, 214 Da [M-H-C_10_H_14_O_5_]^-^and 246 Da [M-H-C_11_H_18_O_6_]^-^. The *m/z* 181, *m/z* 153 and *m/z* 137 were produced by the loss of 268 Da [M-H-C_13_H_16_O_6_]^-^, 296 Da [M-H-C_14_H_16_O_7_]^-^ and 312 Da [M-H-C_14_H_16_O_8_]^-^. *m/z* 181 and *m/z* 137 indicated the presence of dihydrocaffeic acid and *m/z* 235 showed the presence of ferulic acid with any other acidic moiety and *m/z* 413 indicated the presence of ferulic acid with dihydrocaffeic acid by binding with each other with propane-1,2 di-ol chain. [28] so the compound was considered as 3-dihydroferuloyl-1-dihydrocaffeoyl propane-1,2-diol.

The deprotonated molecular ion for compound (17) was displayed at *m/z* 451. The actual mass of the compound was 452 *a*.*m*.*u*. From [M-H]^-^ the loss of 17 Da [M-H-OH]^-^ yielded *m/z* 434. The *m/z* 415, *m/z* 413, *m/z* 407 and *m/z* 391 were generated by the loss of 36 Da [M-H-2H_2_O]^-^, 38 Da [M-H-2H_2_O+H_2_]^-^, 44 Da [M-H-CO_2_]^-^and 60 Da [M-H-C_2_H_4_O_2_]^-^. *m/z* 365, *m/z* 339, *m/z* 320 and *m/z* 305 were obtained with the loss of 86 Da [M-H-C_4_H_6_O_2_]^-^, 112 Da [M-H-C_6_H_8_O_2_]^-^, 132 Da [M-H-C_8_H_4_O_2_]^-^ and 146 Da [M-H-C_9_H_6_O_2_]^-^. *m/z* 283, *m/z* 255, *m/z* 225, *m/z* 200, *m/z* 173 and *m/z* 153 were resulted upon the loss of 168 Da [M-H-C_9_H_10_O_2_+H_2_O]^-^,196 Da [M-H-C_10_H_12_O_4_]^-^, 226 Da [M-H-C_11_H_14_O_5_]^-^, 251 Da [M-H-C_13_H_15_O_5_]^-^, 278 Da [M-H-C_14_H_14_O_6_]^-^, and 298 Da [M-H-C_14_H_18_O_7_]^-^. on MS^3^ data It gave peaks of *m/z* 134, *m/z* 193 which showed the presence of feruloyl group. The base peak *m/z* 413 indicated the presence of feruloyl group with some acidic moiety or other functional groups [28]. So, from above fragmentation the compound (17) was identified as 3-[(3-ethynyl) caffeoyl-2-oxo-propyl] ferulic acid.

The deprotonated molecular ion appeared at *m/z* 479 for compound (18). The actual mass of the compound was 480 *a*.*m*.*u*. [M-H]^-^ suffered the loss of 18 Da [M-H-H_2_O]^-^, 36 Da [M-H-2H_2_O]^-^ and 44 Da [M-H-CO_2_]^-^ Yielded *m/z* 461, *m/z* 443 and *m/z* 435. The *m/z* 405, *m/z* 369, *m/z* 343, *m/z* 311 and *m/z* 281 were obtained from precursor ion by the loss of 74 Da[M-H-C_3_H_6_O_2_]^-^, 110 Da[M-H-C_6_H_6_O_2_]^-^, 136 Da[M-H-C_8_H_8_O_2_]^-^, 168 Da[M-H-C_9_H_12_O_3_]^-^ and 198 Da[M-H-C_10_H_14_O_4_]^-^. *m/z* 255, *m/z* 218, *m/z* 201, *m/z* 171 and *m/z* 149 were generated by the loss of 224 Da [M-H-C_10_H_8_O_6_]^-^, 261 Da [M-H-C_11_H_17_O_7_]^-^, 278 Da [M-H-C_12_H_22_O_7_]^-^, 308 Da [M-H-C_13_H_24_O_8_]^-^ and 330 Da [M-H-C_14_H_18_O_9_]^-^. *m/z* 443 regarding literature showed the presence of diferuloyl moiety with glycerol [28]. So, the compound was considered as 3-[1,3-dihydroxy-3-(4-hydroxy-3-methoxy phenyl) propoxy]-2,3-dihydroxy propyl ferulic acid.

### 3.4. The compounds identified from TER/MeOH

The quasi molecular ion peak [M-H]^-^ appeared at *m/z* 137 with actual mass of 138 *a*.*m*.*u*. [M-H]^-^ with the loss of 15 Da [M-H-CH_3_^·^]^-^ gave *m/z* 122. The *m/z* 109 and *m/z* 94 were produced due to the loss of 28 Da [M-H-CO]^-^and 43 Da [M-H-C_2_OH_3_]^-^.The *m/z* 93 (phenoxide ion) with the removal of [M-H-CO_2_]^-^indicated the presence of benzoic acid. The *m/z* 81, *m/z* 75 and *m/z* 65 were generated by the loss of 56 Da [M-H-2CO]^-^, 62 Da [M-H-COOH+OH]^-^ and [M-H-C_2_O_3_]^-^. *m/z* 65 was the characteristic of benzene group. Regarding reported literature [23] and at *m/z* 75 the loss of COOH+OH^·^ showed the presence of benzoic acid with hydroxy group as a functional group the whole conversation and fragmentation suggested that compound (1) was 2-hydroxy benzoic acid.

The deprotonated molecular ion peak for compound (2) appeared at *m/z* 151 with actual mass of 152 *a*.*m*.*u*. [M-H]^-^ with loss of 15 Da [M-H-CH_3_^·^] gave ^-^*m/z* 136 by homolytic cleavage. The *m/z* 133 was the cause of 18 Da [M-H-H_2_O]^-^. From it the removal of 2 Da provided *m/z* 131. The loss of 44 Da [M-H-CO_2_]^-^ generated *m/z* 107. Base peak *m/z* 93 was obtained by the subsequent loss of 58 Da [M-H-CO_2_+CH_2_^-^]^-^ indicated the presence of benzoic acid derivative [23]. From it the loss of 4 Da produced *m/z* 89. The *m/z* 79, *m/z* 71 and *m/z* 59 were generated by the loss 72 Da [C_3_H_4_O_2_]^-^,80 Da [M-H-C_5_H_4_O]^-^ and 9 Da [M-H-C_6_H_4_O]^-^. Thefragmentation scheme prompted compound (2) was tentatively considered as 4-hydroxy acetophenone.

Compound (3) displayed deprotonated molecular ion at *m/z* 153. The actual mass of the compound was 154 *a*.*m*.*u*. The fragment ion at *m/z* 138 was produced by the loss of 15 Da [M-H-CH_3_^·^]^-^and the 30 Da [M-H-CO+H_2_]^-^corresponding to *m/z* 123. The base peak *m/z* 109 (catechol) was assigned for 44 Da [M-H-CO_2_]^-^showed the presence of benzoic acid moiety with two adjacent hydroxyl group. The *m/z* 95 and *m/z* 93 were resulted upon the loss of 58 Da [M-H-C_2_H_2_O_2_]^-^ and 60 Da [M-H-CO_2_+O^·^]^-^.The daughter ion peaks *m/z* 83 and *m/z* 69 were generated by the loss of 70 Da [M-H-C_3_H_2_O_2_]^-^ and 84 Da [M-H-C_4_H_4_O_2_]^-^.The base peak was matched with literature so according to reported literature the presence of protocatechuic acid was expected [28] the fragmentation pattern proclaimed that compound (3) was assigned as Protocatechuic acid.

The deprotonated molecular ion [M-H]^-^ for compound (4) was produced at *m/z* 163. The actual mass of the compound was 164 *a*.*m*.*u*. [M-H]^-^ suffered the loss of 15 Da [M-H-CH_3_^·^]^-^ resulted *m/z* 148. The MS^2^fragment ion *m/z* 135 was obtained with loss of 28 Da [M-H-CO]^-^. Base peak *m/z* 119 (4-hydroxy vinyl benzene) was generated via the loss of [M-H-CO_2_]^-^indicated the presence of coumaric acid [25]. Fragment ions *m/z* 105, *m/z* 93 and *m/z* 83 were produced by the loss of 58 Da [M-H-C_2_H_2_O_2_]^-^,70 Da [M-H-C_3_H_2_O_2_]^-^ and 80 Da [M-H-C_5_H_4_O]^-^.*m/z* 93 was the characteristics of phenoxide ion the *m/z* 75 and *m/z* 59 corresponding to 88 Da [M-H-C_3_H_3_O_2_+OH^·^] and 104 Da [M-H-C_7_H_4_O]^-^. *m/z* 75 was the characteristic of benzene.according to base peak corresponding to reported literature [25, 45] and fragmentation scheme the compound (4) was identified as *p*-coumaric acid.

Compound (5) exhibited deprotonated precursor ion [M-H]^-^ at *m/z* 187 with actual mass of 188 *a*.*m*.*u*. Fragment ion *m/z* 172 resulted due to loss of 15 Da [M-H-CH_3_]^-^. The*m/z* 169 was brought out by 18 Da [M-H-H_2_O]^-^. The *m/z* 159, *m/z* 143, *m/z* 141 were obtained with the loss of 28 Da [M-H-CO]^-^,44 Da [M-H-CO_2_]^-^and 46 Da [M-H-CO_2_+H_2_]^-^. The loss of CO_2_ showed the presence acidic group. The base peak *m/z* 125 [25] resulted due to 62 Da [M-H-COOH+OH^·^]^-^ indicated the presence of gallic acid. From base peak *m/z* 123 and *m/z* 83 were obtained by the loss of 2 Da (H_2_) and 42 Da (C_2_H_2_O). The *m/z* 107, *m/z* 97, *m/z* 73 and *m/z* 57 were generated with the loss of 80 Da [M-H-CH_4_O_4_]^-^, 90 Da [M-H-C_3_H_6_O_3_]^-^, 114 Da [M-H-C_4_H_2_O_4_]^-^and 130 Da [M-H-C_5_H_6_O_4_]^-^. The base peak *m/z* 125 according to published data [46, 47] showed the presence of gallic acid. So, from whole entire fragmentation scheme or base peak evidence from literature the compound (5) was assigned as monohydrate of gallic acid.

The Pseudo deprotonated molecular ion appeared at *m/z* 202 for compound (6). The actual mass of the compound was 203 *a*.*m*.*u*. The *m/z* 188 and *m/z* 187 were obtained with the loss of 14 Da (CH_2_) and 15 Da (CH_3_^·^). Further fragmentation was proceeded from *m/z* 187 because the compound was considered adduct of dihydrofurano coumarin with the help of literature. The other fragment ions *m/z* 174, *m/z* 166, *m/z* 158, *m/z* 146, and *m/z* 143 and were generated due to loss of 13 Da (CH), 21 Da (H_2_O+H_2_+H^·^), 29 Da (CHO), 41 Da (C_2_HO), 44 Da (CO_2_) and *m/z* 140 was obtained due to loss of 3Da (H_2_+H^·^) from *m/z* 143. The product ions *m/z* 126, *m/z* 116, *m/z* 115, *m/z* 94, *m/z* 88 and *m/z* 71 were resulted with the loss of 61 Da (C_2_H_5_O_2_), 71 Da (C_3_H_3_O_2_), 72 Da (C_3_H_4_O_2_), 93 Da (C_5_HO_2_), 99 Da (C_4_H_3_O_3_), 116 Da (C_8_H_4_O) regarding literature [48, 49] *m/z* 187, *m/z* 174, *m/z* 146 were the characteristic of dihydrofurano coumarin so the compound (6) was considered as adduct of dihydrofurano coumarin.

The deprotonated molecular ion appeared at *m/z* 207 for compound (7). The actual mass of the compound was 208 *a*.*m*.*u*. The fragment ions *m/z* 205, *m/z* 192, *m/z* 189, *m/z* 179, *m/z* 177, *m/z* 163 and *m/z* 161 were generated with loss of 2 Da [M-H-H_2_]^-^, 15 Da [M-H-CH_3_]^-^, 18 Da [M-H-H_2_O]^-^, 28 Da [M-H-CO]^-^, 30 Da [M-H-CH_2_O]^-^, 44 Da [M-H-CO_2_]^-^, and 46 Da[M-H-C_2_H_6_O]^-^and other fragment ions *m/z* 145, *m/z* 135, *m/z* 122, *m/z* 119, *m/z* 109, *m/z* 93, *m/z* 85 and *m/z* 71 were resulted upon the loss of 62 Da [M-H-C_2_H_6_O_2_]^-^, 72 Da [M-H-C_3_H_4_O_2_]^-^, 85 Da [M-H-C_4_H_5_O_2_]^-^, 88 Da [M-H-C_3_H_4_O_3_]^-^, 98 Da [M-H-C_5_H_6_O_2_]^-^, 114 Da [M-H-C_5_H_6_O_3_]^-^, 122 Da [M-H-C_7_H_6_O_2_]^-^, and 136 Da [M-H-C_8_H_8_O_2_]^-^. From literature [27] and overall fragmentation pattern the compound (7) was considered as derivative of ferulic acid.

The deprotonated pseudomolecular ion at *m/z* 215 corresponding to compound (8). The actual mass of the compound was 216 *a*.*m*.*u*. [M-H]^-^ yielded *m/z* 213, *m/z* 197 and *m/z* 187 with the loss of 2 Da [M-H-H_2_]^-^,18 Da [M-H-H_2_O]^-^ and 28 Da [M-H-CO]^-^. The base peak *m/z* 179 was the cause of 36 Da [M-H-2H_2_O]^-^ [25] showed presence of caffeic acid from it *m/z* 161, *m/z* 153 and *m/z* 143 were generated with the loss of 18 Da (H_2_O), 26 Da (2CH) and 36 Da (2H_2_O). The *m/z* 161 and *m/z* 143 were the characteristic fragment ion peaks of caffeic acid [28]. From *m/z* 179, *m/z* 131 and *m/z* 119 were obtained by the loss of 48 Da (CO_2_+2H_2_) and 60 Da (CO+O^·^). From *m/z* 119 the loss of 18 Da (H_2_O) and 30 Da (CH_2_O) resulted *m/z* 101 and *m/z*89. from base peak *m/z* 179 the loss of 108 Da (C_6_H_4_O_2_) gave *m/z* 71. From above justification and fragmentation pattern the compound (8) assigned as dihydrate of caffeic acid.

The deprotonated ion for compound (9) was displayed at *m/z* 217. The actual mass of the compound was 218 *a*.*m*.*u*. [M-H]^-^ suffered the loss of 2 Da [M-H-H_2_]^-^15 Da [M-H-CH_3_]^-^, 18 Da [M-H-H_2_O]^-^ and 36 Da [M-H-2H_2_O]^-^ yielded *m/z* 215, *m/z* 202, *m/z* 199, *m/z* and *m/z* 181 showed the presence of dihydrocaffeic acid [28, 39] from it the loss of 2 Da initiated base peak *m/z* 179 [25] indicated the presence of caffeic acid. From base peak the *m/z* 161, *m/z* 143, *m/z* 131, *m/z* 119 and *m/z* 71 were generated by the subsequent losses of 18 Da (H_2_O), 36 Da (2H_2_O), 48 Da (CO_2_+2H_2_), 60 Da (CO_2_+O^·^) and 108 Da (C_6_H_4_O_2_). The *m/z* 161and *m/z* 143 were the characteristic daughter ion peaks of caffeic acid. *m/z* 119 represented the 4-hydroxy vinyl benzene. From precursor ion the loss of 44 Da [M-H-CO_2_]^-^gave *m/z* 173 and from it the loss of 2 Da provided *m/z* 171 from *m/z* 119 the loss of 18 Da (H_2_O) resulted *m/z* 101. From it the loss of 12 Da yielded *m/z* 89 and from it the loss of 24 Da gave *m/z* 65 was also the characteristic of benzene. By observing fragmentation scheme, it was assumed that caffeic acid is present in derivatized form. So, the compound (9) was probably considered as dihydrate of dihydrocaffeic acid.

Compound (10) showed deprotonated molecular ion at *m/z* 229 with actual mass of 230 *a*.*m*.*u*. [M-H]^-^ with the loss of 16 Da (O^·^) gave *m/z* 213. The loss of 18 Da [M-H-H_2_O]^-^initiated *m/z* 211. *m/z* 201, *m/z* 193, *m/z* 185 and *m/z* 167 were generated with the loss of 28 Da [M-H-CO]^-^, 36 Da [M-H-2H_2_O]^-^, 44 Da [M-H-CO_2_]^-^and 62 Da [M-H-CO_2_+H_2_O]^-^.Fragment ions *m/z* 147, *m/z* 144, *m/z* 119 and *m/z* 109 were obtained by the loss of 82 Da [M-H-C_4_H_2_O_2_]^-^,86 Da [M-H-C_4_H_6_O_2_]^-^, 110 Da [M-H-CH_2_O_6_]^-^ and 120 Da [M-H-C_2_O_6_]^-^. From *m/z* 109 the loss of 14 Da produced *m/z* 95. From it the loss of 12 Da and 24 Da brought out *m/z* 83 and *m/z* 71. From fragmentation and base peak regarding literature [29] it was suggested that compound (10) was identified as derivative of dehydroascorbic acid.

The compound (11) displayed quasi molecular ion peak at *m/z* 237 and the actual mass of the compound was 238 *a*.*m*.*u*. [M-H]^-^ in MS^2^ yielded *m/z* 223, *m/z* 205, *m/z* 194 and *m/z* 177 with the loss of 15 Da [M-H-CH_3_^·^]^-^, 32 Da [M-H-O_2_]^-^, 44 Da [M-H-CO_2_]^-^ and 60 Da [M-H-C_2_H_4_O_2_]^-^. The *m/z* 163 resulted upon the loss of 74 Da [M-H-C_3_H_6_O_2_]^-^was the characteristic of coumaric acid. From it the loss of 2 Da produced *m/z* 161. The loss of 12 Da gave *m/z* 149 from m/z 161. From precursor ion the loss of 118 Da [C_4_H_6_O_4_]^-^ yielded *m/z* 119 the base peak was the characteristic of 4-hydroxy vinyl benzene. From it the loss of 2 Da brought out *m/z* 117. The *m/z* 97 and *m/z* 81 had resulted by the loss of 140 Da [M-H-C_8_H_12_O_2_]^-^and 156 Da [M-H-C_7_H_8_O_4_]^-^. From above discussion and reported literature [28] the compound (11) was considered as 1*-O*-coumaroyl glycerol.

Compound (12) showed deprotonated ion at *m/z* 253. The actual mass of the compound was 254 *a*.*m*.*u*. [M-H]^-^ suffered the loss of 2 Da [M-H-H_2_]^-^, 18 Da [M-H-H_2_O]^-^, 28 Da [M-H-CO]^-^and 44 Da [M-H-CO_2_]^-^ yielded *m/z* 251, *m/z* 235, *m/z* 225 and *m/z* 209. From *m/z* 225 base peak the loss of 28 Da (CO) gave *m/z* 197. The *m/z* 209 with the loss of 28 Da (CO) yielded *m/z* 181. From it the loss of 14 Da brought out *m/z* 167. From precursor ion the loss of 96 Da [M-H-C_6_H_8_O]^-^yielded *m/z* 158. From *m/z* 167 the loss of 14 Da gave *m/z* 153. From parent ion *m/z* 135, *m/z* 123, *m/z* 97, *m/z* 85 and *m/z* 75 were generated by the loss of 118 Da [M-H-C_8_H_6_O]^-^, 130 Da [M-H-C_8_H_2_O_2_]^-^, 156 Da [M-H-C_7_H_8_O_4_]^-^, 168 Da [M-H-C_10_O_3_]^-^ and 178 Da [M-H-C_9_H_6_O_4_]^-^. The base peak *m/z* 225 was matched with literature and *m/z* 209, *m/z* 197, *m/z* 181 and *m/z* 135 were the charachteristic of diadzen [50] from fragmentation and base peak it was decided to assigned compound (12) as diadzen.

The deprotonated molecular ion appeared at *m/z* 255. The actual mass of the compound (13) was 256 *a*.*m*.*u*. The fragment ions *m/z* 253, *m/z* 240, *m/z* 223, *m/z* 211, *m/z* 195, *m/z* 181 and *m/z* 175, *m/z* 167, and *m/z* 151 were generated due to loss of 2 Da [M-H-H_2_]^-^, 15 Da [M-H-CH_3_^·^], 32 Da [M-H-O_2_]^-^, 44 Da[M-H-CO_2_]^-^, 60 Da[M-H-CO2+O^·^]^-^, 74 Da[M-H-C_6_H_2_]^-^, 80 Da[M-H-C_4_O_2_]^-^, 88 Da [M-H-C_7_H_4_]^-^ and 104 Da [M-H-C_8_H_8_]^-^. The *m/z* 127, *m/z* 109, *m/z* 93 and *m/z* 85 were obtained with the loss of 128 Da [M-H-C_9_H_4_O]^-^, 146 Da [M-H-C_9_H_6_O_2_]^-^, 162 Da [M-H-C_9_H_6_O_3_]^-^ and 170 Da[M-H-C_11_H_6_O_2_]^-^. From fragmentation and literature [41, 42] the compound (13) was considered as Pinocembrin.

Compound (14) displayed deprotonated molecular ion at *m/z* 269. The actual mass of the compound was 270 *a*.*m*.*u*. [M-H]^-^generated *m/z* 267, *m/z* 254, *m/z* 241 and *m/z* 225 with the loss of 2 Da [M-H-H_2_]^-^, 15 Da [M-H-CH_3_^·^]^-^, 28 Da [M-H-CO]^-^ and 44 Da [M-H-CO_2_]^-^. The loss of 14 Da (CH_2_), 28 Da (CO) from *m/z* 225 gave *m/z* 211 and *m/z* 197. The loss of 2 Da (H_2_) from *m/z* 197 brought out *m/z* 195. The *m/z* 173, *m/z* 159 and *m/z* 153 were resulted from precursor ion due to loss of 96 Da [M-H-C_6_H_8_O]^-^, 110 Da [M-H-C_6_H_6_O_2_]^-^ and 116 Da [M-H-2O_2_+C_4_H_4_]^-^.The *m/z* 151, *m/z* 131, *m/z* 121, *m/z* 97 and *m/z* 93 were generated by the loss of 118 Da [M-H-C_8_H_6_O]^-^, 138 Da [M-H-C_7_H_6_O_3_]^-^,148 Da[M-H-C_9_H_8_O_2_]^-^, 172 Da [M-H-C_10_H_4_O_3_]^-^ and [M-H-C_9_H_4_O_4_]^-^.So according to literatcure *m/z* 269, *m/z* 241, *m/z* 225, *m/z* 197 and *m/z* 151 were the characteristics of genistein [51]. The compound was considered Genistein.

The deprotonated molecular ion appeared at *m/z* 281. The actual mass of compound (15) was 282 *a*.*m*.*u*. The fragment ions *m/z* 266, *m/z* 263, *m/z* 249, *m/z* 237 and *m/z* 233 were generated due to the loss of 15 Da [M-H-CH_3_^·^]^-^, 18 Da [M-H-H_2_O]^-^, 32 Da[M-H-O_2_]^-^, 44 Da [M-H-CO_2_]^-^ and 48 Da [M-H-CO_2_+2H_2_]^-^.The other fragment ions *m/z* 219, *m/z* 207, *m/z* 193, *m/z* 182 and *m/z* 163 were obtained with the loss of 62 Da [M-H-COOH+OH^·^]^-^, 74 Da [M-H-C_2_H_2_O_3_]^-^, 88 Da [M-H-COOH+CO_2_]^-^ 99 Da [M-H-C_2_H_11_O_4_]^-^ and 118 Da [M-H-C_4_H_6_O_4_]^-^ and fragment ions *m/z* 145, *m/z* 123, *m/z* 117, *m/z* 97 and *m/z* 88 were resulted by the loss of 136 Da [M-H-C_4_H_8_O_5_]^-^,158 Da [M-H-C_5_H_2_O_6_]^-^, 164 Da [M-H-C_5_H_8_O_6_]^-^, 184 Da [M-H-C_7_H_4_O_6_]^-^ and 194 Da [M-H-C_9_H_10_O_2_+CO_2_]^-^. The base peak appeared at *m/z* 237 by Comparing with literature [28] so, it was considered as derivative of 1-*O*-coumaroyl glycerol derivative.

The [M-H]^-^appeared at *m/z* 289. The actual mass of the compound (16) was 290 *a*.*m*.*u*. The *m/z* 271, *m/z* 259, *m/z* 247 and *m/z* 245 were obtained by the loss of 18 Da (H_2_O), 30 Da (CH_2_O), 42 Da C_2_H_2_O and) 44 Da (C_2_H_4_O) from [M-H]^-^. The *m/z* 231 was justified by the loss of 14 Da CH_2_ from *m/z* 245. The loss of 36 Da 2H_2_O two water molecules from *m/z* 245 gave peak at *m/z* 209. From [M-H]^-^ the loss of 84 Da (C_4_H_4_O_2_), 86 Da (C_4_H_6_O_2_), 102 Da (C_4_H_6_O_3_), 110 Da (C_6_H_6_O_2_), 124 Da (C_7_H_8_O_2_) and 152 Da (C_8_H_8_O_3_). resulted *m/z* 205, *m/z* 203, *m/z* 187, *m/z* 179, *m/z* 165 and *m/z* 137. The *m/z* 125, *m/z* 109 and *m/z* 97 were obtained by the loss of 164 Da (C_9_H_8_O_3_), 180 Da (C_9_H_8_O_4_), 193 Da (C_10_H_9_O_4_) the *m/z* 83 was justified by the loss of 14 Da (CH_2_) from *m/z* 97. From above discussion and literature [28] the compound was identified as Catechin.

The deprotonated molecular ion peak for compound (17) appeared at *m/z* 303. The actual mass of the compound was 304 *a*.*m*.*u*. The *m/z* 287, *m/z* 285, *m/z* 267, *m/z* 257 and *m/z* 241 were obtained with the loss of 16 Da [M-H-O^·^]^-^,18 Da[M-H-H_2_O]^-^,36 Da [M-H-2H_2_O]^-^, 46 Da[M-H-CO_2_+H_2_]^-^ and 62 Da [M-H-C_2_H_2_O_2_+2H_2_]. *m/z* 241 with loss of 16 Da(O^·^) and 24 Da (2C) brought out *m/z* 225 and *m/z* 217. The *m/z* 217 with the loss of 4 Da (2H_2_) gave *m/z* 213. The *m/z* 199, *m/z* 185, *m/z* 175 and *m/z* 157 were resulted by the loss of 104 Da [M-H-C_4_H_8_O_3_]^-^, 118 Da [M-H-C_7_H_2_O_2_]^-^, 128 Da [M-H-C_6_H_8_O_3_]^-^and 146 Da [M-H-3O_2_+C_4_H_2_]^-^. The *m/z* 157 with loss of 16 Da (O^·^) gave *m/z* 141. *m/z* 141 brought out *m/z* 113 with the loss of 28 Da (C_2_H_4_). *m/z* 113 gave *m/z* 111 with the loss of 2 Da (H_2_). According to literature [52] *m/z* 303, *m/z* 241, *m/z* 285, *m/z* 217, *m/z* 213 and *m/z* 175 were the characteristics of dihydro quercetin so the compound (17) was assigned as dihydroquercetin.

The deprotonated molecular ion appeared at *m/z* 319. The actual mass of the compound (18) was 320 *a*.*m*.*u*. The *m/z* 304, *m/z* 301, *m/z* 287, *m/z* 275, *m/z* 257, *m/z* 239, *m/z* 224 and *m/z* 197 resulted by the loss of 15 Da [M-H-CH_3_^·^]^-^, 18 Da [M-H-H_2_O]^-^, 32 Da [M-H-O_2_]^-^, 44 Da [M-H-CO_2_]^-^, 62 Da [M-H-CO+O_2_+H_2_]^-^, 80 Da [M-H-2O2+O^·^]^-^, 95 Da [M-H-C_5_H_3_O_2_]^-^ and 122 Da [M-H-C_6_H_2_O_3_]^-^. The *m/z* 239 gave *m/z* 177 with the loss of 62 Da(CO+O_2_+H_2_). Precursor ion with the loss of 154 Da [M-H-C7H6O4]- gave *m/z* 165. The *m/z* 275 by the loss of 130 Da (C_6_H_10_O_3_) brought out *m/z* 145. The *m/z* 125 was obtained with the loss of 194 Da [M-H-C_9_H_6_O_5_]- from deprotonated ion and *m/z* 125 brought *m/z* 107 and *m/z* 97 with the loss of 18 Da (H_2_O) and 28 Da (CO) according to literature [53] *m/z* 319, *m/z* 301, *m/z* 257 and *m/z* 125 were the characteristic of ampelopsin. So, the compound (18) was assigned as ampelopsin.

The deprotonated ion appeared at *m/z* 377. The actual mass of the compound (19) was 378 *a*.*m*.*u*. The *m/z* 359, *m/z* 345, *m/z* 341 and *m/z* 333 were generated with the loss of 18 Da [M-H-H_2_O]^-^, 32 Da [M-H-O_2_]^-^, 36 Da [M-H-2H_2_O]^-^and 44 Da[M-H-CO_2_]^-^. *m/z* 341 brought out *m/z* 315, *m/z* 297 and *m/z* 279 by the loss of 26 Da [C_2_H_2_], 44 Da [CO_2_] and 62 Da [C_2_H_5_O_2_]^-^. *m/z* 377 gave *m/z* 245 with the loss of 132 Da [M-H-C_5_H_8_O_4_]^-^. The*m/z* 245 brought *m/z* 221 by the loss of 24 Da [2C]. *m/z* 377 with the loss of 162 Da[M-H-C_6_H_10_O_5_] gave *m/z* 215. *m/z* 215 with the loss of 18 Da [H_2_O] produced *m/z* 197. The *m/z* 377 with the loss of 162 Da+36 Da [M-H-C_6_H_10_O_5_+2H_2_O]^-^ resulted *m/z* 179. From *m/z* 179 the loss of 18 Da [H_2_O] and 42 Da [C_2_H_2_O] gave *m/z* 161 and *m/z* 137. *m/z* 137 gave *m/z* 113 with the loss of 26 Da (C_2_H_2_). According to literature the [27, 28] *m/z* 341 was the characteristics of caffeoyl glucoside and *m/z* 179, *m/z* 161 were the characteristic of caffeic acid. So, the compound (19) was considered as 6-*O*-caffeoyl glucoside dihydrate.

Thedeprotonated peak of compound (20) appeared at *m/z* 385. The actual mass of the compound was 386 *a*.*m*.*u*. The loss of 18 Da [M-H-H_2_O]^-^ gave the product ion *m/z* 367. The *m/z* 349, *m/z* 341, *m/z* 325, *m/z* 305 and *m/z* 303 were generated with significant losses of 36 Da [M-H-2H_2_O]^-^, 44 Da [M-H-CO_2_]^-^, 60 Da [M-H-COOH+CH_3_^·^]^-^, 80 Da [M-H-COOH+OH^·^+H_2_O]^-^, and 82 Da [M-H-C_4_H_2_O_2_]^-^. The *m/z* 293, *m/z* 269, *m/z* 267, *m/z* 259, *m/z* 231 and *m/z* 217 were brought out by the losses of 92 Da [M-H-OCH_3_^·^+OH^·^+H_2_O]^-^, 116 Da [M-H-C_5_H_4_O_2_+H_2_O+H_2_]^-^, 118 Da [M-H-C_5_H_6_O_2_+H_2_O+H_2_]^-^, 126 Da [M-H-C_7_H_10_O_2_]^-^, 154 Da [M-H-C_8_H_10_O_3_]^-^ and 168 Da[M-H-C_9_H_12_O_3_]^-^. The fragment ions *m/z* 203, *m/z* 190, *m/z* 179, *m/z* 163, *m/z* 145, *m/z* 139 and *m/z* 117 were generated with the loss of 182 Da [M-H-C_10_H_14_O_3_]^-^, 195 Da [M-H-C_10_H_11_O_4_]^-^, 206 Da [M-H-C_11_H_10_O_4_]^-^, 222 Da [M-H-C_11_H_10_O_5_]^-^, 240 Da [M-H-C_11_H_11_O_5_+OH^·^]^-^, 246 Da [M-H-C_13_H_10_O_5_]^-^and 268 Da [M-H-C_14_H_4_O_6_]^-^ The *m/z* 203, *m/z* 190, *m/z* 163 and *m/z* 145 were the characteristic peaks of ferulic acid derivative [28]. From above discussion the compound was identified as feruloyl methyl caffeic acid.

The deprotonated molecular ion peak for compound (21) appeared at *m/z* 387 with actual mass of 388 *a*.*m*.*u*. The 1^st^ fragment ion peak obtained with the loss of 18 Da [M-H-H_2_O]^-^ at *m/z* 369. The *m/z* 343, *m/z* 317, *m/z* 305, *m/z* 269, *m/z* 235, and *m/z* 203 were resulted due to the loss of 44 Da [M-H-CO_2_]^-^, 70 Da [M-H-C_3_H_2_O_2_]^-^, 82 Da [C_4_H_2_O_2_]^-^,118 Da [M-H-C_5_H_6_O_2_+H_2_O+H_2_]^-^, 152 Da [M-H-C_8_H_8_O_3_]^-^ and 184 Da [M-H-C_9_H_12_O_4_]^-^. The fragment ions *m/z* 190, *m/z* 179, *m/z* 163, *m/z* 145, *m/z* 132 and *m/z* 119 were generated upon the significant losses of 197 Da [M-H-C_10_H_13_O_4_]^-^, 208 Da [M-H-C_11_H_12_O_4_]^-^, 224 Da [M-H-C_11_H_12_O_5_]^-^, 242 Da [M-H-C_11_H_14_O_6_]^-^, 255 Da [M-H-C_12_H_15_O_6_] and 268 Da [M-H-C_11_H_11_O_5_+COOH]^-^. The *m/z* 203, *m/z* 190, *m/z* 179, *m/z* 163, and *m/z* 145 were the characteristic of ferulic acid derivative [28]. So, the compound (21) was considered as feruloyl methyl dihydrocaffeic acid.

The deprotonated molecular ion of compound (22) was displayed at *m/z* 411 the actual mass of compound was 412 *a*.*m*.*u*. The *m/z* 393, *m/z* 367, *m/z* 349, *m/z* 331, *m/z* 287, *m/z* 259, *m/z* 245 and *m/z* 217 were brought out by the loss of 18 Da [M-H-H_2_O]^-^, 44 Da [M-H-CO_2_]^-^, 62 Da [M-H-C_2_H_4_O+H_2_O]^-^, 80 Da [M-H-C_3_H_12_O_2_]^-^, 124 Da [M-H-C_7_H_8_O_2_]^-^, 152 Da [M-H-C_9_H_12_O_2_]^-^, 166 Da [M-H-C_9_H_10_O_3_]^-^and 194 Da [M-H-C_10_H_10_O_4_]^-^. The fragment ions were appeared at *m/z* 203, *m/z* 190, *m/z* 176, *m/z* 152 and *m/z* 134 upon significant loss of 208 Da [M-H-C_11_H_12_O_4_]^-^, 221 Da [M-H-C_12_H_13_O_4_]^-^, 235 Da [M-H-C_12_H_11_O_5_]^-^, 252 Da [M-H-C_13_H_7_O_6_]^-^and 277 Da [M-H-C_14_H_13_O_6_]^-^.The fragment ions *m/z* 203 and *m/z* 190 and *m/z* 134 were the characteristic of ferulic acid derivative [28]. The compound (22) was assigned as feruloyl methyl ethenyl caffeate.

For compound (23) deprotonated molecular ion peak appeared at *m/z* 413 with actual mass of 414 *a*.*m*.*u*. Fragment ions *m/z* 398, *m/z* 369, *m/z* 345, *m/z* 327, *m/z* 315 and *m/z* 303 were obtained with the loss of 15 Da [M-H-CH_3_^·^]^-^, 44 Da [M-H-CO_2_]^-^, 68 Da [M-H-C_4_H_4_O]^-^, 86 Da [M-H-C_4_H_6_O_2_]^-^, 98 Da [M-H-C_5_H_6_O_2_]^-^, and 110 Da [M-H-C_6_H_6_O_2_]^-^. The *m/z* 273, *m/z* 259, *m/z* 245, *m/z* 235, *m/z* 217 and *m/z* 203 were generated with the loss of 140 Da [M-H-C_8_H_8_O_2_+2H_2_]^-^, 154 Da [M-H-C_8_H_8_O_2_+H_2_O]^-^, 168 Da [M-H-C_9_H_10_O_2_+H_2_O]^-^, 178 Da[M-H-C_10_H_10_O_3_]^-^, 196 Da[M-H-C_10_H_12_O_4_]^-^, 210 Da[M-H-C_11_H_14_O_4_]^-^. The *m/z* 190, *m/z* 177, *m/z* 161, *m/z* 152 and *m/z* 135 were produced due to loss of 223 Da [M-H-C_12_H_15_O_4_]^-^, 236 Da [M-H-C_12_H_12_O_5_]^-^, 252 Da [M-H-C_12_H_12_O_6_]^-^, 261 Da [M-H-C_13_H_9_O_6_]^-^and 278 Da [M-H-C_14_H_14_O_6_]^-^. The *m/z* 398, *m/z* 369, *m/z* 235, *m/z* 217, *m/z* 177, *m/z* 161 and *m/z* 135 were the characteristic peaks of 1-*O*-feruloyl-3-*O*-*p*-coumaroyl glycerol [29]. The compound (23) was considered as 1-*O*-feruloyl-3-*O*-*p*-coumaroyl glycerol.

The deprotonated molecular ion peak displayed at *m/z* 431 for compound (24). The actual mass of the compound was 432 *a*.*m*.*u*. The fragment ions *m/z* 416, *m/z* 413, *m/z* 401, *m/z* 387, *m/z* 373, *m/z* 345, *m/z* 313 and *m/z* 305 were generated by the loss of 15 Da [M-H-CH_3_]^-^, 18 Da [M-H-H_2_O]^-^, 30 Da [M-H-CH_2_O]^-^, 44 Da [M-H-CO_2_]^-^, 58 Da [M-H-C_2_H_2_O_2_]^-^, 86 Da [3CO+H_2_]^-^, 118 Da [M-H-C_4_H_6_O_4_]^-^, and 126 Da [M-H-C_4_H_8_O_4_+3H_2_]^-^. The fragment ions *m/z* 277, *m/z* 261, *m/z* 241, *m/z* 218, and *m/z* 187 were resulted with the loss of 154 Da [M-H-C_5_H_14_O_5_]^-^, 170 Da [M-H-C_7_H_6_O_5_]^-^, 190 Da [M-H-C_6_H_6_O_7_]^-^, 218 Da [M-H-C_12_H_5_O_4_]^-^ and 244 Da[M-H-C_10_H_12_O_7_]^-^. The *m/z* 167 was obtained with the loss 220 Da [C_9_H_16_O_6_]^-^ from *m/z* 387. The *m/z* 149 was initiated upon the loss of 112 Da [C_5_H_4_O_3_]^-^from *m/z* 261. The last fragment ion *m/z* 125 the base peak appeared due to loss 306 Da [M-H-C_15_H_14_O_7_]^-^. From literature [34, 35] and fragmentation scheme the compound (24) was identified as Isovitexin.

The deprotonated molecular ion appeared at *m/z* 513. The actual mass of the compound (25) was 514 *a*.*m*.*u*. The *m/z* 495, *m/z* 477, *m/z* 469, *m/z* 455, *m/z* 433, *m/z* 395 and *m/z* 377 were resulted with the loss of 18 Da [M-H-H_2_O]^-^, 36 Da [M-H-2H_2_O]^-^, 44 Da [M-H-CO_2_]^-^, 58 Da [3H_2_O+2H_2_]^-^, 80 Da [M-H-C_5_H_4_O]^-^, 118 Da [M-H-C_8_H_6_O]^-^ and 136 Da [M-H-C_8_H_8_O_2_]^-^. The product ions *m/z* 349, *m/z* 329, *m/z* 293, *m/z* 283, *m/z* 255, *m/z* 233, *m/z* 191 and *m/z* 165 were produced due to loss of 164 Da [M-H-C_9_H_8_O_3_]^-^, 184 Da [M-H-C_9_H_12_O_4_]^-^, 220 Da [M-H-C_10_H_20_O_5_]^-^, 230 Da [M-H-C_10_H_14_O_6_]^-^, 258 Da [M-H-C_12_H_18_O_6_]^-^, 280 Da [M-H-C_13_H_12_O_7_]^-^, 322 Da [M-H-C_15_H_14_O_8_]^-^and 348 Da [M-H-C_17_H_16_O_8_]^-^.regarding literature [34, 35]. The compound (25) was considered as 6-*O*-Pentenoyl glucopyranosyl-6-*C*-apigenin,

The deprotonated molecular ion peak appeared at *m/z* 577. The actual mass of the compound (26) was 578 *a*.*m*.*u*. The fragment ions *m/z* 562, *m/z* 559, *m/z* 541, *m/z* 533, *m/z* 503, *m/z* 485 and *m/z* 471 were produced with the loss of 15 Da [M-H-CH_3_^·^], 18 Da [M-H-H_2_O]^-^, 36 Da [M-H-2H_2_O]^-^, 44 Da [M-H-CO_2_]^-^, 74 Da [M-H-C_3_H_6_O_2_]^-^, 92 Da [M-H-2CO+2H_2_O]^-^, 106 Da [C_6_H_2_O_2_]^-^. The fragment ions *m/z* 451, *m/z* 425, *m/z* 407, *m/z* 381, *m/z* 357, *m/z* 331, *m/z* 299, *m/z*, *m/z* 289, *m/z* 245, *m/z* 199 and *m/z* 175 were generated by the loss of 126 Da [M-H-C_6_H_6_O_3_]^-^, 152 Da [M-H-C_8_H_8_O_3_]^-^, 170 Da [M-H-C_8_H_10_O_4_]^-^, 196 Da [M-H-C_10_H_12_O_4_]^-^, 220 Da [M-H-C_11_H_8_O_5_]^-^, 246 Da[M-H-C_13_H_10_O_5_]^-^, 278 Da[M-H-C_14_H_14_O_6_]^-^, 288 Da[M-H-C_15_H_12_O_6_]^-^, 332 Da [M-H-C_15_H_12_O_6_+CO_2_]^-^, 378 Da [M-H-C_19_H_22_O_8_]^-^ and 402 Da [M-H-C_21_H_22_O_8_]^-^. By Comparing base peak and deprotonated molecular ion peak with [54, 55] literature and fragmentation scheme the compound (26) was considered as procyanidin B1.

### 3.5. DPPH assay

The radical scavenging activity of samples (TE(1), TE(2), TER/MeOH) were calculated by using formula AC-AS/AC×100 where AC = Absorbance of control, AS = Absorbance of sample.

**Fig 8:**
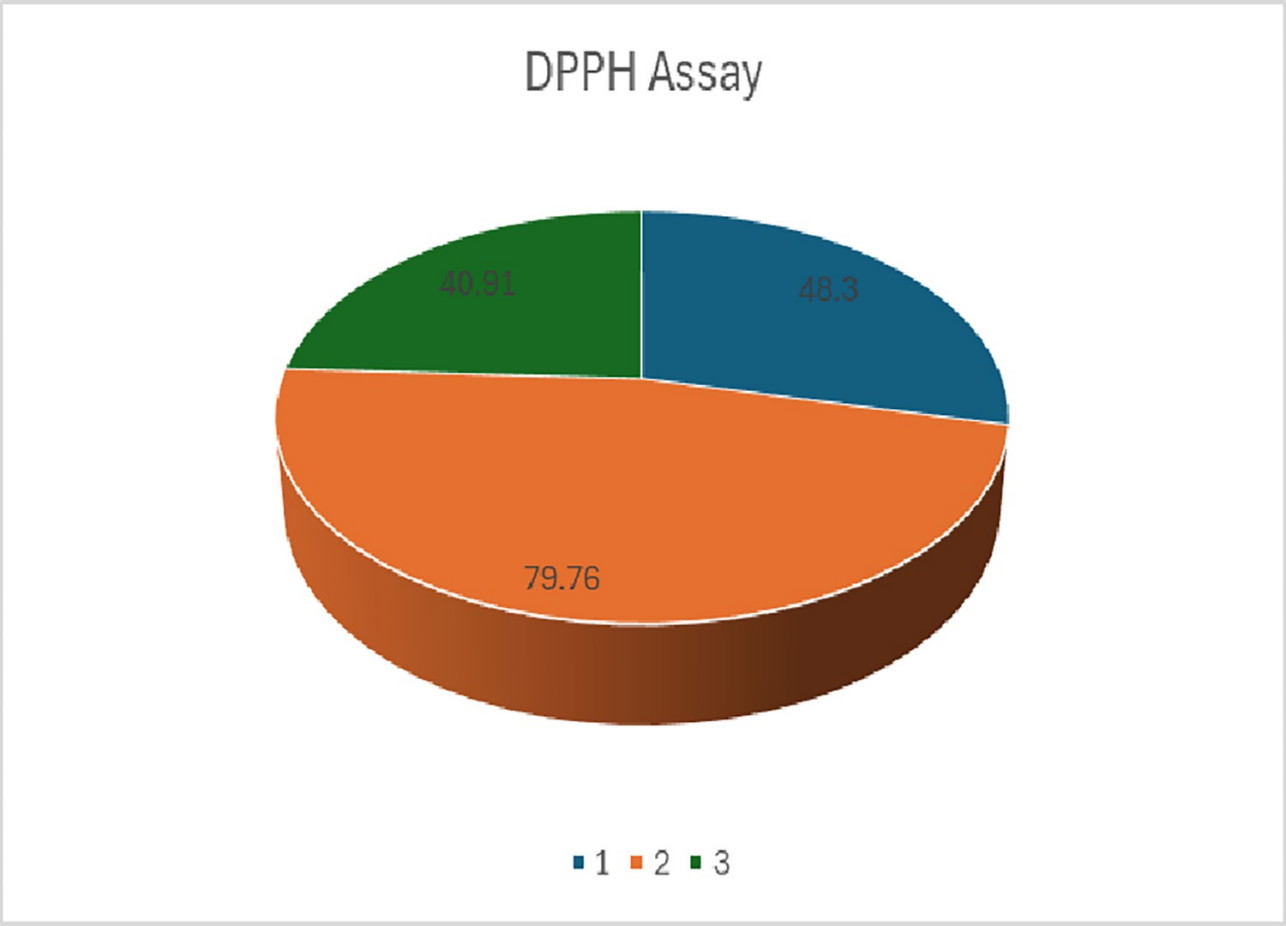
The pictorial representation of DPPH radical scavenging activity of TE(1), TE(2) and TER/MeOH by piechart. Fig 8 indicated the pie chart of DPPH for TE(1), TE(2) and TER/MeOH. Blue color indicated TE(1), The orange color represented TE(2) and green color showed the TER/MeOH.

The results of DPPH activity was mentioned in **Table 5.** The Pie chart in Fig 8. easily described the antioxidant activity of each extract. The TE(2) Showed higher antioxidant activity at 79.76% than TE(1). TER/showed less antioxidant at 40.91% activity than TE(1) and TE(2). From that it was concluded that the aerial parts of *Typha elephantina* showed more antioxidant activity than roots due to presence of more Significant comounds like ascorbic acid, Isovitexin, coumarin derivative, and 3 piperidine derivative due to which it showed highest antioxidant activity.

**Table 5:** The DPPH Radical Scavenging activity of TE(1), TE(2) and TER/MeOH.

S.No	Sample codes	Absorbance	Radical Scavenging Activity
1	TE(1)	0.35	48.3%
2	TE(2)	0.137	79.76%
3	TER/MeOH	0.4	40.91%

### 3.6. Conclusion

In this work, a report on the phenolic composition of aerial parts and roots of *Typha elephantina* is being explored for the first time using Tandem mass spectrometry (MS/MS). This qualitative analysis provided a valuable fingerprint of the main metabolites in *Typha elephantina*. The DPPH assay described their antioxidant activity. A total of 62 compounds were identified from MS/MS profile of aerial parts TE(1)/MeOH, TE(2)/MeOH and roots TER/MeOH in negative mode. It mainly constituted caffeic acid, quinic acid, galactonic acid, ascorbic acid, coumaric acid and ferulic acid derivatives, dihydrofurano coumarin, apigenin, diglycosides of quercetin and isorhamnetin, verbascoside and forsythoside A, alkaloids, flavanone, isoflavone, dihydroflavonol, catechin, Isovitexin and its derivative the potent components which are previously reported in other plants but not in *Typha elephantina*. The DPPH antioxidant assay results showed that aerial parts TE(1), TE(2) showed more antioxidant activity than roots TER/MeOH. This activity affirmed that *Typha elephantina* is a rich source of phenolic compounds. These compounds have significant bibliographic data regarding the application of these bioactive components for human health. The extended properties of this plant are endorsed by their usage as animal feed, human food, in the nutraceutical and pharmaceutical industries. Future prospects include isolation and determining the bioavailability of these compounds to highlight their medicinal and nutritional attributes. Cell-culture and *in vivo* studies shall be directed to assess their bioaccessibility for commercial purposes.

## Supporting information

S1 File TE(1) 18: *Typha elephantina* aerial parts brown color extract 18 compounds. (DOCX)

S2 File TE(2) 18: *Typha elephantina* aerial parts green color extract 18 compounds. (DOCX)

S3 File TER (26): *Typha elephantina* roots extract 26 compounds. (DOCX)

S1 Graphical abstract (PNG)
