# Supplementary material for: Unraveling the bioactive constituents of Typha elephantina: A comprehensive phytochemical analysis by tandem mass spectrometry
Source: PLoS One. 2024 Dec 5;19(12):e0311549. doi: 10.1371/journal.pone.0311549 (PMC11620470; doi:10.1371/journal.pone.0311549)
Supplement: S1 File — (DOCX) [file pone.0311549.s001.docx]

The compounds identified from Aerial parts TE(1)/MeOH

1. 3,4 Dihydroxy Benzoic Acid (Protocatechuic Acid)

The compound (1) deprotonated molecular ion peak [M-H]^-^ appeared at m/z 153 [M-H-15]^-^ was due to cleavage of CH_3_^·^ at m/z 138 the [M-H-32]- was due to loss of O_2_ at m/z 121 most abundant peak indicated the presence of benzoic acid the significant [M-H-CO_2_]^-^ 44 Da loss at m/z 109 clearly showed the presence of benzoic acid with two adjacent hydroxyl groups further m/z 96 and m/z 95 were obtained with [M-H-2CO+H]^-^, [M-H-C_2_O_2_H_2_]^-^ and the last product ion peak at m/z 93(Ali *et al*., 2021) of phenoxide ion with loss of 60Da [M-H-CO_2_+O^·^] tentatively confirmed that compound (1) was 3,4 dihydroxy benzoic acid.

Ali, A., Bashmil, Y. M., Cottrell, J. J., Suleria, H. A., & Dunshea, F. R. (2021). Lc-ms/ms-qtof screening and identification of phenolic compounds from australian grown herbs and their antioxidant potential. *Antioxidants*, *10*(11), 1770

1. Para Coumaric Acid

The molecular ion peak appeared at m/z 163 the loss of 15 Da [M-H-CH_3_^·^]^-^gave peak at m/z 148 the m/z 131 was due to the loss of two oxygen [M-H-O_2_]^-^ indicate the presence of two hydroxy groups or may be an acidic moeity the base peak at m/z 119 (Szewczyk *et al*., 2021), (Sinosaki, *et al*., 2020) with loss of CO_2_ expected the presence of coumaric acid the m/z 105 and m/z 95 appeared with significant lossess of 58Da and 68 Da [M-H-C_2_H_2_O_2_]^-^[M-H-C_3_O_2_]^-^ the whole analysis tentatively recognize that compound 2 was para coumaric acid.

1. Phenyl 2,2,2 -Trihydroxyacetate

The deprotonated precusor ion peak [M-H]^-^ appeared at m/z 183 the loss of 13Da [M-H-CH]^-^ gave peak at m/z 170 the m/z 164 was due to the loss of 18+1 Da [M-H-H_2_O-H]^-^ The compound 3 exhibited base peak at m/z 147 (Beelders *et al*., 2014) via the loss of 36Da [M-H-H_2_O] probably assumed the presence of phenyl acetic acid derivative. The loss of 44Da CO_2_ [M-H-CO_2_] from parent ion showed peak at m/z 139 the m/z 114 and 112 were assigned for the loss of 69Da and 71Da [M-H-3OH+H_2_O][M-H-C_3_O_2_H_3_] the whole entire fragmentation tentatively suggested that compound was Phenyl 2,2,2 -Trihydroxyacetate.

1. Acetylene Shikimate

The compound 4 deprotonated molecular ion peak [M-H]^-^ appeared at m/z 197 the loss of 13Da [M-H-CH]^-^ gave fragment ion at m/z 184 with [M-H-H_2_O] loss of 18Da the daughter ion appeared at m/z 179 the m/z 161 base peak (Ben Said *et al*., 2017) was produced via the loss of 36 Da [M-H-2H_2_O]^-^ showed that compond was supposed to be shikimic acid derivative the daughter ion peak at m/z 153 was generated with the loss of 44 Da [M-H-CO_2_]^-^ indicate the presence of acidic moiety the m/z 141, m/z 126, m/z 111, m/z 97 and m/z 75 appeared with significant lossess of 56Da, 71Da, 86Da, 100 Da and 122 Da [M-H-2CO]^-^,[M-H-C_3_H_3_O_2_],[M-H-C_3_H_2_O_3_],[M-H-C_5_H_6_O_2_] and [M-H-C_6_H_2_O_3_] The whole fragment ions and lossess tentatively identified the compound 4 was acetylene Shikimate

1. hydrate of Caffeic Acid


The compound 5 showed deprotonated molecular ion peak at m/z 215 with the loss of 18 Da [M-H-H_2_O]^-^ It gave daughter ion peak at m/z 197 the sequential loss of 36Da from precursor ion [M-H-2H_2_O]^-^ at m/z 179 base peak attributed the presence of caffeic acid (Sinosaki, *et al*., 2020)(Kang *et al*., 2016) the m/z 177 was produced by the loss of 2Da from base peak and the loss of 18Da H_2_O from base peak showed daughter ion peak at m/z 161 and loss of 36Da from base peak was responsible for m/z 143. The m/z 161 and m/z143 were charachteristic fragment ion peaks of caffeoyl moiety (Ben Said *et al*., 2017) the m/z 119 charachteristic peak of vinyl 4-hydroxy benzene was produced by the loss of 60 Da CO_2_+O indicated the presence of COOH and hydroxyl group the m/z 101,m/z 89 and 71 were generated due to the lossess of 78Da (CH_2_O_4_), 90Da (C_2_H_2_O_4_) and 108 Da (C_6_H_4_O_2_) from base peak from above arguments it was proclaimed that compond 5 was tentatively considered the derivatives of caffeic acid.

1. Hydrate of dihydro caffeic acid

The deprotonated ion peak or precursor ion peak [M-H]^-^ of the compound 6 was produced at m/z 217 showed product ion peak at m/z 199 with the loss of 18 Da[M-H-H_2_O]^-^ and m/z 181 was the characteristic peak of dihydrocaffeic acid appeared by the loss of 36 Da [M-H-2H_2_O]^-^ the loss of 2Da from m/z 181 gave base peak at m/z 179 indicated the presence of caffeic acid (Sinosaki, *et al*., 2020)(Kang *et al*., 2016) from them with the loss of 6Da (6H) the m/z 173 was initiated the loss of 18Da(H_2_O) and 36 Da (2H_2_O) from base peak generated m/z 161 and m/z 143 were charachteristic fragment ion peaks of caffeic acid (Ben Said *et al*., 2017) the m/z 119 charachteristic peak of vinyl 4-hydroxy benzene was produced by the loss of 60 Da CO_2_+O indicated the presence of acidic moeity with hydroxyl group the m/z 97 and 89 were assigned for the lossess of 82Da(C_4_H_2_O_2_) and 90Da (C_2_H_2_O_4_) the above discussion attributed the presence of caffeic acid which tentatively describe compound 6 as a dihydrated dihydro caffeic acid.

1. 4-Hydroxy-3 Methoxy-4(3,4,5-Trioxo-Tetrahydro Furan) Butanal

The compound 7 presented the deprotonated molecular ion peak [M-H]^-^ at m/z 229 the product ion peak appeared at m/z 215 via the loss of 14Da [M-H-CH_2_]^-^the loss of 18Da [M-H-H_2_O]^-^ was the cause to yield m/z 211 m/z 199 was produced due to removal of 30Da [M-H-CO+H_2_] from them the loss of 2Da provide peak at m/z 197 the fragment ion m/z 193 was initiated by the loss of 36 Da [M-H-2H_2_O] the loss of 38 Da [M-H-2H_2_O+H_2_] and 44 Da[M-H-CO_2_] gave peak at m/z 191 and 185 with the loss of 58 Da [M-H-C_3_H_6_O] the m/z 171 was obtained the m/z 191 and 211 were the characteristic peaks of dehydroascorbic acid (Cioffi *et al*., 2000) indicated the presence of dehydroascorbic acid the m/z 155 was produced due to the 74Da [M-H-C_3_H_6_O_2_]^-^m/z 151 was obtained by the loss of 40Da(C_3_H_4_) from m/z 191the m/z 140, m/z 130 and m/z 127 were produced by the loss of 89Da[M-H-C_4_H_9_O_2_]^-^, 99Da [M-H-C_3_O_4_]^-^ and 102Da[M-H-C_4_H_6_O_3_]^-^ from m/z 127 the loss of 16Da give rise m/z 111 from them the loss of 13Da(CH),14Da(CH_2_),16Da(O) and 18Da(H_2_O) generated m/z 98, m/z 97, m/z 95 and m/z 93 the last two daughter peaks at m/z 76 and m/z75 were assigned for the loss of 153Da[M-H-C_7_H_5_O_4_] and 154Da[M-H-C_6_H_2_O_5_] from the whole fragmentation pattern it was decided that compound 7 was probably the derivative of dehydroascorbic acid.

1. 1*-O*-Coumaroyl Glycerol

The deprotonated precursor ion appeared at m/z 237 the loss of 18Da [M-H-H_2_O]^-^ gave fragment ion peak at m/z 219 the loss two water molecule [M-H-2H_2_O]^-^ give rise m/z 201 further the m/z179 was generated due to removal of 58 Da [M-H-C_2_H_2_O_2_] the prominent peak m/z 163 appeared by the loss of 74Da [M-H-C_3_H_6_O_2_] indicated the loss of glycerol type moiety the m/z 156 and m/z 145 were produced with the elimination of 81Da [M-H-C_5_H_5_O] and 92 Da[M-H-C_3_H_8_O_3_] m/z 163 and m/z 145 were the charachteristic peaks of coumaric acid (Kang *et al.,* 2016) m/z 139 appeared by the removal of 98 Da [M-H-C_4_H_2_O_3_] the base peak m/z 119 was obtained by the loss of 118 Da [M-H-C_4_H_6_O_4_] indicated the presence of 4-hydroxy phenyl benzene the m/z 99, m/z 93, m/z 83 were produced by the loss of 138Da [M-H-C_4_H_10_O_5_]^-^,145Da[C_6_H_8_O_4_]^-^,154Da [M-H-C_7_H_6_O_4_]^-^ respectively the m/z 93 was the charahteristic peak of phenol from above arguments the compond 8 was tentatively identified as 1*-O*-Coumaroyl Glycerol (Kang *et al.,* 2016)

Kang, J., Price, W. E., Ashton, J., Tapsell, L. C., & Johnson, S. (2016). Identification and characterization of phenolic compounds in hydromethanolic extracts of sorghum wholegrains by LC-ESI-MSn. *Food chemistry*, *211*, 215-226

1. Glucaroyl 4-Hydroxy Benzoate

The deprotonated molecular ion peak [M-H]^-^ appeared at m/z 329 fragment ion peak m/z was produced by the loss of 18Da [M-H-H_2_O]^-^the m/z 293 was due to the elimination of 36 Da two water molecules [M-H-2H_2_O]^-^the loss of 54Da [M-H-3H_2_O] 3 water molecules gave peak at m/z 275 product ion m/z 271 intense peak was produced due to 58 Da with the loss of [M-H-COOH+CH]^-^the loss of 2Da from them induced m/z 269 the m/z 243 appeared via the loss of 86 Da [M-H-C_4_H_4_O+H_2_O] 100Da loss [M-H-C_4_H_4_O+O_2_] yield m/z 229 the removal of 18Da H_2_O from them gave peak at m/z 211 the base peak at m/z 209(Fernández *et al*., 2019) was due to the loss of 120 Da[M-H-C_7_H_4_O_2_] from deprotonated ion peak indicated the presence of glucaric acid The m/z 201 was obtained by the loss of 28 Da (CO) from m/z 229 the loss of 36 Da(2H_2_O) and 58Da(COOH+CH) from base peak provide m/z 173 and m/z 151 the daughter ion peak m/z 137 was generated by the loss of 192Da[M-H-C_6_H_8_O_7_]^-^ indicated the presence of benzoic acid (Ali *et al*., 2021) the removal of 14Da (CH_2_) from m/z 137 initiated m/z 123 the last fragment ion peak at m/z 93 was produced due to loss of 236Da [M-H-C_7_H_8_O_9_]^-^was the charachteristic peak of phenol the overall fragmentation outline reveal the presence of glucaric acid with benzoic acid the compound 9 was tentatively identified as Glucaroyl 4-Hydroxy Benzoate.

1. 6-*O*-Caffeoyl glucoside dihydrate

The deprotonated molecular ion [M-H]^-^ appeared at m/z 377 the intense fragment ion appeared at m/z 345 due to the loss of 32Da [M-H-O_2_]^-^ base peak m/z 341 was obtained by the loss of 36Da [M-H-2H_2_O]^-^which was charachteristic of caffeoyl hexoside from them loss of 28Da (CO) gave daughter ion peak at m/z 313 from him the elimination of 28Da (CO) bring about m/z 285 the loss of 96 Da from base peak (C_3_H_12_O_3_) provide peak at m/z 245 from deprotonated precursor ion peak the loss of 162 Da C_6_H_10_O_5_ indicated the loss of glucose yield m/z 215 which prompted the presence of glucose the removal of 18Da(H_2_O) from m/z 215 initiated m/z 197 the m/z 179 was produced from m/z 215 with the loss of 36Da(2H_2_O) was the characteristic of caffeoyl moiety the m/z161,m/z 143, m/z 119 were generated from m/z 179 via the loss of 18Da(H_2_O), 36Da(2H_2_O),60Da(CO_2_+O) the m/z 161, m/z 143 were the characteristic peaks of caffeic acid and m/z 119 was the characteristic of 4-hydroxy vinyl benzene the entire fragmentation scheme and lossess indicated the presence of caffeic acid with glucose so the compound 10 was tentatively considered as Dihydrated 6-*O* of caffeoyl glucoside.

1. 6-*O*- dihydroCaffeoyl glucoside dihydrate

The deprotonated molecular ion peak [M-H]^-^ appeared at m/z 379 produced daughter ion peak at m/z 347 by the loss of 32Da [M-H-O_2_]^-^the loss of 38Da [M-H-2H_2_O+H_2_]^-^ Provide base peak at m/z 341 which was the charachteristic of caffeoyl-*O*-hexoside but the loss of 38 indicated hydration of caffeoyl group the loss of 20 Da (H_2_O+H_2_)and 64Da (CH_4_O_3_) from m/z 341 gave product ion at m/z 321 and m/z 277 from them the loss of 14 Da(CH_2_) satisfied the m/z 263 from him the removal of 36Da(2H_2_O) Justified m/z 227 the loss of 162Da [M-H-C_6_H_10_O_5_]^-^ generated m/z 217 showed the presence of glucose the elimination of 18Da(H_2_O) from m/z 217 justified the m/z 199 the substraction of 38 Da(2H_2_O+H_2_) from m/z 217 provide m/z 179 which was the charachteristic of caffeoyl group but the loss of 38 Da showed the hydration of caffeic acid the m/z 161 and m/z 131 were obtained by the loss of 18Da(H_2_O) and 48Da(CO_2_+2H_2_) from m/z 179 the m/z 161 was the characteristic peak of caffeic acid from all over description it was assumed that compound 11 was probably the Dihydrate of 6-*O*-dihydro caffeoyl glucoside

1. 1. 3′[ 5-(1-Hydroxyethyl) Cyclohexa-2,4-Diene-1,2-Diol] Apigenin

2. 3′-*C*- 4-Propenyl Cyclo hexane 1,2 di ol Apigenin

The compound 12 exhibited deprotonated molecular ion peak[M-H]- appeared at m/z 423 m/z 405 was obtained by the loss of 18Da[M-H-H_2_O]^-^ the loss of 36Da [H_2_O+CH_4_+H_2_] yielding the base peak m/z 387 compared with literature indicated the presence of apigenin with presence of moiety at 3´position (Yao *et al*., 2017) the loss of 58Da[M-H-C_4_H_10_]^-^gave peak at m/z 365 from them the substraction of [M-H-H_2_O+H_2_]^-^and [M-H-C_3_H_2_O]^-^ provided m/z 345 and m/z 311 the [M-H-C_7_H_17_O]^-^ was the cause of m/z 306 m/z 299 appeared due to [M-H-C_8_H_12_O]^-^ with m/z 311 suffered the loss of 36Da(2H_2_O) yielding m/z 275 from m/z 387 base peak the loss of 138 Da(C_8_H_7_O+OH+H_2_) gave peak at m/z 249 the reduction of 198 Da [M-H-C_11_H_16_O_2_+H_2_O]^-^brought m/z 225 which was the charachteristic of apigenin among them the loss of 2Da provide m/z 223 the m/z 197 led to the [M-H-C_13_H_6_O_4_]^-^showed the presence of 4-Propenyl Cyclo hexane 1,2 di ol m/z161 and m/z 139 were obtained by the loss of 36Da (2H_2_O) and 58Da(C_4_H_10_) the whole fragmentation path way indicated the presence of apigenin with propenyl-cyclohexane diol moiety the compound 12 was tentatively considered as 3′-*C*- 4-Propenyl Cyclohexane 1,2 di ol Apigenin.

1. Isorhamnetin 3-*O*-Glucopyranoside

The deprotonated [M-H]^-^ ion for compound 13 appeared at m/z 477 suffered the loss of 35Da[M-H-H_2_O+OH]^-^yielding m/z 442 the loss of 57Da the m/z 420 appeared with 57 Da[M-H-CO_2_+CH]^-^the m/z 388 and m/z 358 were obtained with loss of 89 Da [M-H-C_3_H_5_O_3_]^-^and 119 Da [M-H-C_4_H_7_O_4_]^-^and m/z 330 is the cause of 147 Da[M-H-C_5_H_7_O_5_]^-^ indicated the loss of glucose moiety among them the substraction of 15Da(CH_3_) initiated the base peak at m/z 315 which showed the presence of isorhamnetin (Ding *et al*., 2008), (Du *et al*., 2017),(Ben Said *et al*., 2017) from base peak m/z 286, m/z 271, m/z 244, m/z 221, m/z 151 were obtained by loss of 29 Da(CHO), 44 Da(CO_2_), 71 Da (C_3_H_3_O_2_), 94 Da(C_5_H_2_O_2_), 164 Da(C_9_H_8_O_3_) m/z 151 and 271 were the charachteristic peak of flavonoids from m/z 221loss of 16 Da(O) radical gave peak at m/z 205 m/z among them the loss of 25 Da(C2H) radical brought m/z 180 the above discussion suggested the presence of isorhamnetin with glucose the compound 13 was tentatively identified as Isorhamnetin 3-*O*-Glucopyranoside.

14. 6-*O*-Pentenoyl glucopyranosyl-6-*C*-apigenin

1.


The compound 14 displayed deprotonated molecular ion peak [M-H]^-^at m/z 513 by the loss of 36 Da[M-H-2H_2_O]^-^ m/z 477 was produced the [M-H-CO_2_]^-^44Da gave m/z 469 the loss of 102Da [M-H-C_5_H_10_O_2_]^-^provided m/z 411 m/z 379 was the cause of 134 Da [M-H-C_8_H_6_O_2_]^-^m/z 327 was obtained by the reduction of 186Da[M-H-C_8_H_9_O_4_+OH]^-^among them the loss of 4Da (2H_2_) gave peak at m/z 323 m/z 298 was initiated by the removal of 216 Da [M-H-C_10_H_16_O_5_]^-^among them the loss of 4Da(2H_2_) gave peak at m/z 293 m/z 279,m/z 233, m/z 191 and m/z 172 were generated by the loss of 234 Da [M-H-C_10_H_18_O_6_]^-^,280 Da[M-H-C_13_H_12_O_7_]^-^,322 Da[M-H-C_15_H_14_O_8_]^-^and 341Da[M-H-C_16_H_21_O_8_]^-^ the base peak at m/z 477 matched with reported literature so it give information about the presence of isovitexin and the entire fragmentation path way prompted the presence of isovitexin derivative so the compound 14 was probably considered as

6-*O*-Pentenoyl glucopyranosyl-6-*C*-apigenin

1. Rutin

The deprotonated molecular ion [M-H]^-^ at m/z 609 was of compound 15 suffered the loss of [M-H-H_2_O]^-^ yielding m/z 591 m/z 535 was the cause of 74 Da [M-H-C_3_H_6_O_2_]^-^m/z 491 was obtained due to the loss of 118 Da [M-H-C_5_H_10_O_3_]^-^ the162 Da indicated the presence of sugar [M-H-C_6_H_10_O_5_]^-^generated m/z 447 m/z 385 and m/z 315 were obtained with the loss of 224 Da[M-H-C_8_H_16_O_7_]^-^and 294 Da[M-H-C_11_H_18_O_9_]^-^ the m/z 315 prompted the presence of flavonoid aglycone the m/z 301 base peak was produced by the loss of (162+146)Da [M-H-C_6_H_10_O_4_+C_6_H_10_O_5_]^-^the 162 loss indicated the presence of glucose and 146 loss indicated the presence of rhamnose and m/z 301 base peak showed the presence of Quercetin according to literature (Ding *et al*., 2008),

(Du *et al*., 2017), (Ben Said *et al*., 2017) from aglycone m/z 301 the loss of 30Da (CH_2_O), 57 Da(2CO+H^·^) and 108 Da(C_6_H_4_O_2_) generated m/z 271, m/z 244 and m/z 193 m/z 271 is charachteristic of flavonoid from above arguments and whole fragmentation pathway it was decided that the quercetin is present with glucose and rhamnose the compound 15 was tentatively identified as rutin.

1. Isorhamnetin-3-O-(1-2) Rhamnosyl Glucopyranoside.

1. Ding, S., Dudley, E., Plummer, S., Tang, J., Newton, R. P., & Brenton, A. G. (2008). Fingerprint profile of Ginkgo biloba nutritional supplements by LC/ESI-MS/MS. *Phytochemistry*, *69*(7), 1555-1564.
2. Du, L. Y., Zhao, M., Tao, J. H., Qian, D. W., Jiang, S., Shang, E. X., & Duan, J. A. (2017). The metabolic profiling of isorhamnetin-3-O-neohesperidoside produced by human intestinal flora employing UPLC-Q-TOF/MS. *Journal of chromatographic science*, *55*(3), 243-250.
3. Ben Said, R., Arafa I, H., Usam A, M., Abdullah Sulaiman, A. A., Kowalczyk, M., Moldoch, J., Oleszek, W., Stochmal, A. (2017). Tentative characterization of polyphenolic compounds in the male flowers of Phoenix dactylifera by liquid chromatography coupled with mass spectrometry and DFT. *International journal of molecular sciences*, *18*(3), 512.

The compound 16 deprotonated molecular ion[M-H]^-^appeared at m/z 623 the loss of 36 Da[M-H-2H_2_O]^-^yielding m/z 587 among them the reduction of 84 Da(C_4_H_4_O_2_) and 128 Da (C_6_H_8_O_3_) initiated m/z 503 and m/z 459 from him the removal of 103Da (C_4_O_3_H_7_) provide m/z 355 the loss of (146+162)Da [M-H-C_6_H_10_O_4_+C_6_H_10_O_5_]^-^Produced m/z 315 the 146 loss indicated the presence of rhamnose and 162 loss showed the presence of glucose and m/z 315 represented the aglycone moiety of flavonoid by matching with literature it showed the presence of isorhamnetin the m/z 300, m/z 271, m/z 244 and m/z 189 were generated from m/z 315 by the loss of 15Da(CH_3_), 44Da(CO_2_), 71Da(C_3_H_3_O_2_) and 126 Da(C_5_H_2_O_4_) the m/z 271 is the charachteristic peak of flavonoid from the fragmentation scheme it was suggested that isorhamnetin is present with two sugars glucose and rhamnose the compound 16 was tentatively identified as Isorhamnetin-3-*O*-(1-2) rhamnosyl glucopyranoside.

1. Verbascoside dihydrate

The compound 17 exhibited deprotonated moloecular ion[M-H]^-^ at m/z 659 produced m/z 623 with the loss of 36 Da [M-H-2H_2_O]^-^the m/z 599 and m/z 563 were resulted by the loss of 24 Da[M-H-C_2_]^-^and 60 Da[M-H-C_2_H_4_O_2_]^-^m/z 541 and m/z 474 were obtained by the removal of 82 Da[M-H-C_4_H_2_O_2_]^-^ and 147+2Da [M-H-C_6_H_11_O_4_+H_2_]^-^147 loss indicated the presence of deoxyhexose sugar the 161Da[M-H-C_6_H_9_O_5_] and 147+76 Da[M-H-C_6_H_11_O_4_+C_3_H_8_O_2_]^-^ generated m/z 462 and m/z 400 among them the loss of 3Da [H_2_-H^·^] radical gave peak at m/z 397 m/z 346, m/z 271 and m/z 249 were obtained by the loss of [M-H-C_12_H_16_O_6_+OH+2H_2_]^-^,[M-H-C_10_H_8_O_4_+C_6_H_8_O_5_]^-^and [M-H-C_17_H_20_O_9_+3H_2_] by analysing above fragmentation pattern the presence of rhamnose sugar the base peak m/z 623 indicated the presence of Verbascoside (Göğer *et al*., 2015), (Llorent *et al*., 2015) the compound 17 was tentatively identified as Verbascoside dihydrate.

1. dihydroForsythoside A dihydrate

The compond 18 displayed deprotonated molecular ion peak at m/z 661 suffered the loss of 32 Da(O_2_) yielding m/z 629 the loss of 36+2Da [2H_2_O+H_2_]^-^yielding m/z 623 the base peak among them m/z 613, m/z 562, m/z 493 and m/z 461 were resulted by the sequential loss of 10 Da(5H_2_),61Da(C_2_H_5_O_2_),130Da(C_5_H_6_O_4_) and 162Da(C_6_H_10_O_5_) subsequently the 162 loss indicated the presence of sugar from m/z 461 the loss of 26Da(C_2_H_2_) and 106Da(C_6_H_2_O_2_) justified m/z 435 and m/z 355 from m/z 435 the 61Da (C_2_H_5_O_2_) was the cause of m/z 374 the m/z 311 and m/z 253 were obtained by the loss of 242+72Da(C_11_H_14_O_6_+C_3_H_4_O_2_) and 370Da(C_17_H_22_O_9_) from m/z 623 by fragmentation path way, the base peak m/z 623(Göğer *et al*., 2015), (Llorent *et al*., 2015) and presence of sugar moiety the compound 18 was tentatively identified as dihydrated di hydro forsythoside A or dihydro forsythoside A hydrate
