# Supplementary material for: Unraveling the bioactive constituents of Typha elephantina: A comprehensive phytochemical analysis by tandem mass spectrometry
Source: PLoS One. 2024 Dec 5;19(12):e0311549. doi: 10.1371/journal.pone.0311549 (PMC11620470; doi:10.1371/journal.pone.0311549)
Supplement: S2 File — (DOCX) [file pone.0311549.s002.docx]

The compounds identified from TE(2)

NEGATIVE MODE FULL SCAN

**MS/MS**

1. **3-Amino-1-methyl piperidine**

The deprotonated molecular ion peak appeared at m/z 113 for compound (1) yielding m/z 99 with the loss of 14 Da[M-H-CH_2_]^-^. The m/z 95 was obtained by the loss of 18 Da[M-H-CH_4_+H_2_]^-^and m/z 88, m/z 85 and m/z 81 were generated with the loss of 25 Da[M-H-C_2_H^·^], 28 Da[M-H-C_2_H_4_]^-^and 32 Da[M-H-CH_2_NH_2_+H_2_]^-^m/z 70, m/z 69 and m/z 60 were resulted by the loss of 43 Da[M-H-C_2_H_5_N]^-^, 44 Da[M-H-C_3_H_8_]^-^and 53 Da[M-H-C_4_H_5_^·^]^-^ m/z 59 and m/z 58 were produced by the loss of 54 Da[M-H-C_4_H_6_]^-^and 55 Da[M-H-C_4_H_7_^·^]^-^ from above all fragmentation scheme it was decided that compound was identified as 3-Amino-1-methyl piperidine m/z 113 and m/z 70 (Hussein *et al*., 2016)(Wan et al., 2022) are the peaks of methyl piperidine.

Wan, J., Liao, Y., Liu, J., Du, W., Liu, C., Wei, Y., & Ouyang, Z. (2022). Screening, cloning and functional characterization of key methyltransferase genes involved in the methylation step of 1-deoxynojirimycin alkaloids biosynthesis in mulberry leaves. *Planta*, *255*(6), 121.

Hussein, H. M., Hameed, I. H., & Ubaid, J. M. (2016). Analysis of the secondary metabolite products of ammi majus and evaluation anti-insect activity. *international journal of pharmacognosy and phytochemical Research* *8*(8) 1403-1411.

1. **Piperidine-1-carboxylic acid**

The deprotonated molecular ion appeared at m/z 128 suffered the loss of 17 Da [M-H-OH^·^]^-^hydroxyl radical yielding m/z 111. m/z 110, m/z 85 and m/z 84 were generated with the loss of 18 Da[M-H-H_2_O]^-^, 43 Da [M-H-C_3_H_7_]^-^and 44 Da[M-H-CO_2_]^-^. m/z 83, m/z 57 and m/z 56 were resulted by the loss of 45 Da[M-H-COOH]^-^, 71 Da[M-H-C_2_HNO_2_]^-^and 72 Da[M-H-C_2_H_2_NO_2_]^-^The m/z 84 and m/z 56 regarding literature. (Hussein *et al*., 2016)(Wan et al., 2022) the compound was considered as Piperidine-1-carboxylic acid.

1. **3-Amino-5-hydroxy piperidine-1-methane-diol**

The deprotonated molecular ion peak appeared at m/z 162 with the loss of 15 Da[M-H-CH_3_^·^]^-^methyl radical m/z 146 was obtained. m/z 142, m/z 130 and m/z 128 were generated with the loss of 19 Da[M-H-H_2_O+H^·^]^-^, 31 Da[M-H-OCH_3_^·^]^-^ and 33 Da[M-H-NH_2_OH]^-^. m/z 116, m/z 113, m/z 100 and m/z 98 were generated with the loss of 45 Da[M-H-COOH]^-^, 48 Da[M-H-COOH+H_2_+H^·^]^-^, 61 Da[M-H-CH_3_NO_2_]^-^ and 63 Da[M-H-CH_3_NO_2_+H_2_]^-^. m/z 88, m/z 84, m/z 74, m/z 60 and m/z 59 were resulted by the loss of 73 Da[M-H-C_3_H_7_NO]^-^,77 Da[M-H-C_2_H_7_NO_2_]^-^, 87 Da[M-H-C_3_H_5_NO_2_]^-^, 101 Da [M-H-C_5_H_11_NO]^-^and 102 Da [M-H-C_3_H_6_N_2_O_2_]^-^ The whole fragmentation suggested that compound was considered as 3-Amino-5-hydroxy piperidine-1-methane-diol.

1. **Ascorbic acid**

The deprotonated ion appeared for at m/z 175 with the loss of 13 Da it gave m/z 164 further m/z 162 was obtained due to loss of 2 Da from them m/z 157, m/z 147 , m/z 139 and m/z 129 were resulted by the loss of18 Da [M-H-H_2_O]^-^, 28 Da[M-H-CO]^-^,36 Da[M-H-2H_2_O]^-^and 46 Da[M-H-CO_2_+H_2_]^-^from parent ion the loss of 58 Da[M-H-2CO+H_2_]^-^,60 Da [M-H-C_2_H_4_O_2_]^-^and 64 Da[M-H-2O_2_]^-^generated m/z 117, m/z 115 and m/z 111 from m/z 111 the loss of 40 Da and 15 Da gave m/z 71 and m/z 96 from them 1Da loss gave m/z 95 from deprotonated ion the loss of 90 Da{M-H-C_3_H_6_O_3_]^-^ and 101 Da [M-H-C_4_H_5_O_3_]^-^brought m/z 85 and m/z 74 with the loss of 1 Da gave m/z 73. The deorotonated ion and base peak was the charachteristic of ascorbic acid (Ben *et al*., 2017) so the compound was tentatively assigned as ascorbic acid.

1. **Dihydro caffeic acid**

Compound () exhibited deprotonated ion at m/z 181 yielding m/z 166 with loss of methyl radical [M-H-CH_3_^.^] the m/z 163 base peak appeared due to the loss of 18 Da water molecule from precursor ion [M-H-H_2_O]^-^m/z 161 was obtained by the loss of 20 Da [M-H-H_2_O+H_2_]^-^by the loss of oxygen m/z 149 resulted from parent ion [M-H-O_2_]^-^m/z 131 was produced upon the loss of 50 Da neutral carbon di oxide and 3 hydrogen molecule [M-H-CO_2_+3H_2_]^-^ from deprotonated ion the loss of 62 Da gave m/z 119 [M-H-CO_2_+H_2_O]^-^among them the loss of neutral 3 hydrogen molecule brought m/z 113. m/z101, m/z 97 and m/z 89 were obtained with the loss of 80 Da[M-H-CO_2_+2OH+H_2_]^-^,84 Da[M-H-C_4_H_4_O_2_]^-^and 92 Da [M-H-C_2_H_4_O_2_+O_2_]^-^ regarding literature the m/z 181 indicated the presence of dihydrocaffeic acid and m/z 163, m/z 161 were the charachteristic fragment ions of dihydrocaffeic acid so from whole fragmentation the compound() was considered as dihydrocaffeic acid.

1. Martínez-Huélamo, M., Tulipani, S., Jáuregui, O., Valderas-Martinez, P., Vallverdú-Queralt, A., Estruch, R., ... & Lamuela-Raventós, R. M. (2015). Sensitive and rapid UHPLC-MS/MS for the analysis of tomato phenolics in human biological samples. *Molecules*, *20*(11), 20409-20425.
2. Kang, J., Price, W. E., Ashton, J., Tapsell, L. C., & Johnson, S. (2016). Identification and characterization of phenolic compounds in hydromethanolic extracts of sorghum wholegrains by LC-ESI-MSn. *Food chemistry*, *211*, 215-226.

1. **Quinic Acid**

The compound() showed deprotonated molecular ion at m/z 191 suffered the loss of methyl radical yielding fragment ion m/z 176. The m/z 173 was obtained by the loss of water molecule[M-H-H_2_O] the loss of 2Da from them gave m/z 171 the loss of two water molecules from precursor ion [M-H-2H_2_O]^-^ gave m/z 155 the 42Da[M-H-C_2_H_2_O]^-^ loss resulted m/z 149 the neutral loss of 44 Da [M-H-CO_2_]^-^ showed product ion at m/z 147 an additional loss of two oxygen molecules 64 Da[M-H-2O_2_]^-^ generated m/z 127 regarding literatue was the charachteristic of quinic acid.(Llorent *et al*., 2015) from m/z 127 the loss of 2Da(H_2_) hydrogen molecule gave m/z 125 further from deprotonated ion the loss of two oxygen molecule and one oxygen radical 80 Da[M-H-2O_2_+O^.^]^-^resulted m/z 111 among them the loss of 10Da(5H_2_) gave m/z 101 from precursor ion the loss of 98Da [M-H-3H_2_O+CO_2_]^-^, 106Da[M-H-C_2_H_2_O_3_+O_2_]^-^, 120 Da[M-H-C_4_H_7_O_3_+OH]^-^ and 132Da[M-H-C_5_H_8_O_4_]^-^ resulted m/z 93(catechol), m/z 85(pentanol), m/z 71 and m/z 59 according to literature(Szewczyk et al. 2021) the base peak m/z 85 indicated the presence of quinnic acid from the entire fragmentation pattern and m/z 127, m/z 85 and m/z 111 were the characteristic of quinic acid the compound() was tentatively considered as quinic acid.

1. Llorent-Martínez, E. J., Gouveia, S., & Castilho, P. C. (2015). Analysis of phenolic compounds in leaves from endemic trees from Madeira Island. A contribution to the chemotaxonomy of Laurisilva forest species. *Industrial Crops and Products*, *64*, 135-151.
2. Szewczyk, K., Pietrzak, W., Klimek, K., Miazga-Karska, M., Firlej, A., Flisiński, M., & Grzywa-Celińska, A. (2021). Flavonoid and phenolic acids content and in vitro study of the potential anti-aging properties of Eutrema japonicum (Miq.) Koidz cultivated in Wasabi Farm Poland. *International Journal of Molecular Sciences*, *22*(12), 6219.
3. **Galactonic acid**

^^

The deprotonated precursor ion was obtained at m/z 213 for compound() suffered the loss of 18 Da [M-H-H_2_O]^-^ yielding m/z 195 among them the loss of 10 Da(5H_2_) gave m/z 185 from m/z 195 the loss of 18 Da(H2O), 26Da(C_2_H_2_), and 36 Da(2H_2_O) resulted m/z 177, m/z 169 and m/z 159 m/z 177 the most intense peak was the base peak further in fragmentation scheme the m/z 151, m/z 139 and m/z 129 were produced from m/z 195 by the loss of 44 Da (CO_2_), 56 Da(3H_2_O+H_2_) and 66 Da(COOH+OH+2H_2_). The m/z 129 upon the loss of 4 Da(2H_2_) gave m/z 125 and from m/z 195 the product ions m/z 107, m/z 99 and m/z 79 were generated by the loss of 88 Da(C_3_H_4_O_3_), 96 Da(C_2_H_8_O_4_) and 116 Da(CH_8_O_6_). The base peak m/z 177, and m/z 195, m/z 159 and m/z 129 were the charachteristic of galactonic acid(Göğer *et al*., 2015) so from whole fragmentation pattern and literature information the compound () was presumptively identified as mono hydrate of galactonic acid.

Göğer, F., Köse, Y. B., Göğer, G., & Demirci, F. (2015). Phytochemical characterization of phenolics by LC-MS/MS and biological evaluation of Ajuga orientalis from Turkey.Bangladesh J Pharmacol 10, 639-644.

1. CAFFEIC ACID dihydrate

The quasi-molecular ion was displayed at m/z 215 the loss of 18Da[M-H-H_2_O]^-^,32 Da[M-H-O_2_]^-^and 36 Da[M-H-2H_2_O]^-^resulted m/z 197, m/z 183 and base peak m/z 179 further lossess were carried out from base peak m/z 179. Among them the loss of 2 Da (H_2_), 18 Da(H_2_O), 26 Da(C_2_H_2_) and 36 Da (2H_2_O) brought m/z 177, m/z 161, m/z 153 and m/z 143 further the m/z 129, m/z 119 ,m/z 89 and m/z 79 were obtained from m/z 179 with the loss of 50 Da(H_2_O+CH_2_O+H_2_),a 6oDa(CO3) ,90 Da(C2H2O2+O2) and 100 Da(C4H4O3) regarding literature(Ben *et al*., 2017) m/z 179 was the charachteristic of caffeic acid and m/z 161 and m/z 143 (Sinosaki et al., 2020)(kang et al 2016) were the charchteristic fragment ions of caffeic acid so from above entire discussion it was suggested that compound was identified as dihydrate of caffeic ACID.

1. Sinosaki, N., Tonin, A. P., Ribeiro, M. A., Poliseli, C. B., Roberto, S. B., Silveira, R. D., ... & Meurer, E. C. (2020). Structural study of phenolic acids by triple quadrupole mass spectrometry with electrospray ionization in negative mode and H/D isotopic exchange. *Journal of the Brazilian Chemical Society*, *31(*2) 402-408.
2. Kang, J., Price, W. E., Ashton, J., Tapsell, L. C., & Johnson, S. (2016). Identification and characterization of phenolic compounds in hydromethanolic extracts of sorghum wholegrains by LC-ESI-MSn. *Food chemistry*, *211*, 215-226.
3. Dihydro caffeic acid

The compound exhibited deorotonated molecular ion at m/z 217 suffered the loss of 18 Da[M-H-H2O]- and 36 Da [M-H-2H_2_O]^-^ yielding m/z 199 and base peak m/z 181 further lossess were done from base peak m/z 181. Among the the loss of 2Da(H2), 8Da(4H_2_), 33 Da and 45 Da (COOH) brought m/z 179, m/z 173, m/z 148 and m/z 136 further from m/z 181 m/z 119, m/z 97, m/z 89 and m/z 75 were generated with the loss of 62 Da(CO2+H2O), 85 Da(C4H5O2), 92 Da(COOH+CH2O+OH) and 106 Da(C3H6O4) the m/z 181 base peak was the charachteristic of dihydrocaffeic acid according to literature (Martínez et al., 2015)(kang etal., 2016) so from above fragmentation pattern and literature the compound was probably considered as dihydrate of dihydrocaffeic acid.

1. Martínez-Huélamo, M., Tulipani, S., Jáuregui, O., Valderas-Martinez, P., Vallverdú-Queralt, A., Estruch, R., ... & Lamuela-Raventós, R. M. (2015). Sensitive and rapid UHPLC-MS/MS for the analysis of tomato phenolics in human biological samples. *Molecules*, *20*(11), 20409-20425.
2. Kang, J., Price, W. E., Ashton, J., Tapsell, L. C., & Johnson, S. (2016). Identification and characterization of phenolic compounds in hydromethanolic extracts of sorghum wholegrains by LC-ESI-MSn. *Food chemistry*, *211*, 215-226
3. Dihydro caffeic acid

The deprotonated ion for compound () was obtained at m/z 219 yielding m/z 201, base peak m/z 181 by the loss of 18 Da[M-H-H_2_O]^-^and 38 Da[2H2O+H2] further lossess were carried out from base peak m/z 181 among them m/z 175, m/z 157, m/z 143 and m/z 129 were generated by the loss of 6Da(3H2), 24 Da(C2), 38 Da(2H2O+H2) and 52 Da(CO2+4H2) and m/z 119, m/z 88 and m/z 69 were resulted with the loss of 62 Da(COOH+OH), 93 Da(C_2_O_4_H_5_) and 112 Da(C5H4O3) the m/z 181 was the charachteristic of dihydrocaffeic acid according to literature(Martínez et al., 2015)(kang etal., 2016) so the compound was tentatively identified as dihydrate of dihydrocaffeic acid.

1. Martínez-Huélamo, M., Tulipani, S., Jáuregui, O., Valderas-Martinez, P., Vallverdú-Queralt, A., Estruch, R., ... & Lamuela-Raventós, R. M. (2015). Sensitive and rapid UHPLC-MS/MS for the analysis of tomato phenolics in human biological samples. *Molecules*, *20*(11), 20409-20425.

Kang, J., Price, W. E., Ashton, J., Tapsell, L. C., & Johnson, S. (2016). Identification and characterization of phenolic compounds in hydromethanolic extracts of sorghum wholegrains by LC-ESI-MSn. *Food chemistry*, *211*, 215-226

1. **PINOCEMBRIN**

The deprotonated molecular ion appeared at m/z 255 with the loss of 15 Da[M-H-CH_3_]^-^ gave fragment ion at m/z 240 was a Base peak the parent ion generated m/z 237, m/z 227, m/z 211 and m/z 183 by the loss of 18 Da [M-H-H_2_O]^-^,28 Da[M-H-CO]^-^, 44 Da[M-H-CO_2_]^-^and 72 Da[M-H-CO_2_+CO]^-^the m/z 151, m/z 137, m/z 123, m/z 95 and m/z85 were resulted from m/z 255 with loss of 104 Da[M-H-C_8_H_8_]^-^,118 Da[M-H-C_8_H_6_O]^-^, 132 Da [M-H-C_9_H_8_O]^-^,160 Da[M-H-C_10_H_8_O_2_]^-^and170 Da[M-H-C_11_H_6_O_2_]^-^regarding literature (Bertra ms *et al*., *2013)(* Zhoa *et al.,* 2020*)* m/z 255, m/z 183 and m/z 151 were the characteristics of pinocembrin so from fragmentation and literature the compound () was plausibly identified as pinocembrin.

Bertrams, J., Kunz, N., Müller, M., Kammerer, D., & Stintzing, F. C. (2013). Phenolic compounds as marker compounds for botanical origin determination of German propolis samples based on TLC and TLC-MS. *Journal of Applied Botany and Food Quality*, *86*(1),143-153.

Zhao, X., Zhang, S., Liu, D., Yang, M., & Wei, J. (2020). Analysis of flavonoids in dalbergia odorifera by ultra-performance liquid chromatography with tandem mass spectrometry. *Molecules*, *25*(2), 389.

1. **APIGENIN**

The deprotonated molecular ion appeared at m/z 269 for compound () with the loss of 15 Da methyl radical [M-H-CH_3_]^-^ gave m/z 254 the loss of water molecule from parent ion[M-H-H_2_O]^-^ resulted m/z 251 and m/z 241 Was obtained by the loss of 28 Da[M-H-CO]^-^m/z 225 was produced by the loss of 44Da [M-H-CO_2_]^-^carbon dioxide m/z 223, m/z 213, m/z 197 were generated by the loss of 46 Da[M-H-CO_2_+H_2_]^-^_,_ 56 Da loss [M-H-C_3_H_4_O]^-^and 72 Da[M-H-C_4_H_8_O]^-^from m/z 197 the loss of 2 Da gave m/z 195 m/z 183, m/z 159, m/z 131, m/z 113 and m/z 97 were resulted upon the loss of 86 Da[M-H-C_4_H_6_O_2_]^-^, 110 Da [M-H-C_6_H_6_O_2_]^-^, 138 Da[M-H-C_7_H_6_O_3_]^-^, 156 Da[M-H-C_7_H_8_O_4_]^-^and 172 Da[M-H-C_10_H_4_O_3_]^-^from m/z 113 the loss of 24 Da gave m/z 89. Regarding literature the molecularion, base peak m/z 225 and fragment ions m/z 241 and m/z 197 were the charactristics of Apigenin. So from entire fragmentation pattern and literature information the compound() was tentatively assigned as Apigenin.

Kang, J., Price, W. E., Ashton, J., Tapsell, L. C., & Johnson, S. (2016). Identification and characterization of phenolic compounds in hydromethanolic extracts of sorghum wholegrains by LC-ESI-MSn. *Food chemistry*, *211*, 215-226.

1. **PRUNETIN**

For compound() deprotonated ion appeared at m/z 283 with the loss of 15 Da methyl radical it gave peak at m/z 268 the m/z 255, m/z 240 and m/z 224 were resulted by the loss of 28 Da [M-H-CO]^-^,43Da [M-H-CO+CH_3_]^-^, 59 Da [M-H-CO_2_+CH_3_]^-^from deprotonated ion m/z 207 and m/z 183 were obtained by the loss of 76 Da[M-H-C_3_H_8_O_2_]^-^and 100 Da[M-H-C_5_H_8_O_2_]^-^m/z 183 upon the loss of 2Da hydrogen molecule brought m/z 181 m/z 155, m/z 138, m/z 102 and m/z 93 were generated from parent ion by the loss of 128 Da[M-H-C9H4O]^-^,146 Da[M-H-C_9_H_6_O_2_]^-^, 181 Da[M-H-C_9_H_9_O_4_]^-^and 190 Da [M-H-C_10_H_6_O_4_]^-^. Regarding literature the m/z 283 and m/z 268 were the characteristic of prunetin. So from fragmentation pattern and literature evidence the compound was plausibly identified as prunetin.

Zhoa, X., Zhang, S., Liu, D., Yang, M., & Wei, J. (2020). Analysis of flavonoids in *dalbergia odorifera* by ultra-performance liquid chromatography with tandem mass spectrometry. *Molecules*, *25*(2), 389.

1. 1-*O*-Feruloyl-3-*O*-*p*-Coumaroyl Glycerol

The deprotonated ion appeared at *m/z* 413 the actual weight would be 414 the fragment ions *m/z* 398, *m/z* 369, m/z 354 and *m/z* 313 were generated from deprotonated ion with the loss of 15 Da[M-H-CH_3_]^-^, 44 Da[M-H-CO_2_]^-^,59 Da [M-H-C_2_H_3_O_2_]^-^ and 100 Da [M-H-C_5_H_8_O_2_]^-^*m/z* 293, m/z 267 and m/z 249 were obtained by the loss of 120 Da[M-H-C_2_H_4_O_2_]^-^,146 Da[M-H-C_9_H_6_O_2_]^-^and 165 Da [M-H-_9_H_9_O_3_]^-^ base peak m/z 235 apperared from deprotonated ion with the loss of [M-H-C_10_H_10_O_3_]^-^ from base peak m/z 219, m/z 205 and m/z 193 were obtained by the loss of 16 Da [M-H-O^.^]^-^ oxygen radical, 30 Da[M-H-CH_2_O]^-^and 42 Da[M-H-C_2_H_2_O]^-^from m/z 193 the loss of 16 Da oxygen radical gave m/z 177 from them the loss of 16 Da gave m/z 161 and from base peak m/z 235 the loss of 100 Da (C_4_H_4_O_3_) brought m/z 135. Regarding literature (kang *et al*., 2016)the m/z 413 and base peak 235 was the characteristic of 1-*O*-Feruloyl-3-*O*-*p*-Coumaroyl Glycerol from fragmentation scheme and significant lossess the compound was tentatively considered as of 1-*O*-Feruloyl-3-*O*-*p*-Coumaroyl Glycerol.

Kang, J., Price, W. E., Ashton, J., Tapsell, L. C., & Johnson, S. (2016). Identification and characterization of phenolic compounds in hydromethanolic extracts of sorghum wholegrains by LC-ESI-MSn. *Food chemistry*, *211*, 215-226

-

1. 1,3-*O*-Diferuloyl Glycerol

The molecular ion peak appeared at m/z 443.30 it means the actual mass of the compound assumed 444a.m.u by applying rule of 13 the base formula obtained C_34_H_36_ was not matched with any organic compound so the formula was modified by comparing molecular ion peak and base peak with literature the C_23_H_24_O_9_ was modified formula. The peak at m/z 428 was obtained by the loss of 15Da CH_3_ from m/z 443 the loss 44Da CO_2_ from 443 gives peak at m/z 399 the m/z 369 obtained by the loss of 30 Da CH_2_O from m/z 399 the loss of 16 Da O from m/z 369 gave peak at m/z 353 from m/z 353 the loss of 46Da CH_2_O_2_ gave peak at m/z 307 the peak m/z 293 appeared by the loss of 150 Da C_9_H_10_O_2_ from molecular ion peak m/z 443 the m/z 267 was obtained by the loss of 176Da C_10_H_8_O_3_ from m/z 443 the m/z 267 with the loss of 18Da H_2_O gave peak at m/z 249 the loss of 208Da C_11_H_12_O_4_ from m/z 443 gave base peak at m/z 235 the loss of 18Da H_2_O from m/z 235 gave peak m/z 217 and m/z 207 appeared by the loss of 28Da CO from base peak at m/z 235 the m/z 443 with the loss of 250Da C_13_H_14_O_5_ gave peak at m/z 193 the loss of 268 Da C_13_H_16_O_6_ from m/z 443 gave peak at m/z 175 the m/z 161 appeared by the loss of 282Da C_14_H_18_O_6_ from m/z 443 the m/z 443 with the loss of 294 Da C_14_H_14_O_7_ gave peak at m/z 149 the loss of 15 Da CH_3_ from m/z 149 gave peak at m/z 134. The all over tentative fragmentation suggested that the compound was 1,3-*O-*diferuloylglycerol (kang *et al*., 2016)

Kang, J., Price, W. E., Ashton, J., Tapsell, L. C., & Johnson, S. (2016). Identification and characterization of phenolic compounds in hydromethanolic extracts of sorghum wholegrains by LC-ESI-MSn. *Food chemistry*, *211*, 215-226

1. **3-Dihydro feruloyl-1-dihydrocaffeoyl propane-1,2-diol**
2. **3(dihydro feruloyl)1,2-dihydroxy propyl dihydrocaffeic acid**

The compound() deprotonated molecular ion appeared at m/z 449 yielding m/z 417 with the loss of 32 Da [M-H-O_2_]^-^ from m/z 449 the loss of 36 Da[M-H-H_2_O]^-^ gave m/z 413. m/z 406, m/z 387, m/z 345 and m/z 295 were generated by the loss of 43Da[M-H-CO+CH_3_]^-^,62 Da[M-H-C_2_H_2_O_2_+2H_2_]^-^, 104 Da[M-H-C_5_H_6_O_2_+3H_2_]^-^, and 154 Da[M-H-C_9_H_14_O_2_]^-^m/z 281, m/z 255, m/z 235 and m/z 203 were obtained with the loss of 168 Da[M-H-C_9_H_12_O_3_]^-^, 194 Da[M-H-C_10_H_10_O_4_]^-^, 214 Da[M-H-C_10_H_14_O_5_]^-^and 246 Da [M-H-C_11_H_18_O_6_]^-^the m/z 181, m/z 153 and m/z 137 were produced by the loss of 268 Da[M-H-C_13_H_16_O_6_]^-^,296 Da[M-H-C_14_H_16_O_7_]^-^and 312 Da [M-H-C_14_H_16_O_8_]^-^ m/z 181 and m/z 137 indicated the presence of dihydrocaffeic acid and m/z 235 showed the presence of feruilic acid with any other acidic moiety and m/z 413 indicated the presence of ferulic acic with dihdrocaffeic acid by binding with each other with propyl-1,2 di-ol chain.

Kang, J., Price, W. E., Ashton, J., Tapsell, L. C., & Johnson, S. (2016). Identification and characterization of phenolic compounds in hydromethanolic extracts of sorghum wholegrains by LC-ESI-MSn. *Food chemistry*, *211*, 215-226

17. 3-[(3-ethynyl)caffeoyl-2-oxo-propyl] Ferulic acid

The deprotonated molecular ion for compound() was displayed at m/z 451 the loss of 17 Da [M-H-OH]^-^hydroxyl radical yielding m/z 434. m/z 415, m/z 413, m/z 407 and m/z 391 were generated by the loss of 36 Da[M-H-2H_2_O]^-^, 38 Da[M-H-2H_2_O+H_2_]^-^, 44 Da[M-H-CO_2_]^-^and 60 Da[M-H-C_2_H_4_O_2_]^-^. m/z 365, m/z 339, m/z 320 and m/z 305 were obtained with the loss of 86 Da[M-H-C_4_H_6_O_2_]^-^, 112 Da[M-H-C_6_H_8_O_2_]^-^, 132 Da[M-H-C_8_H_4_O_2_]^-^ and 146 Da[M-H-C_9_H_6_O_2_]^-^. m/z 283, m/z 255, m/z 225, m/z 200, m/z 173 and m/z 153 were resulted due to the loss of 168 Da[M-H-C_9_H_10_O_2_+H_2_O]^-^,196 Da[M-H-C_10_H_12_O_4_]^-^, 226 Da[M-H-C_11_H_14_O_5_]^-^, 251 Da[M-H-C_13_H_15_O_5_]^-^, 278 Da[M-H-C_14_H_14_O_6_]^-^, and 298 Da[M-H-C_14_H_18_O_7_]^-^. ON MS3 DATA IT GIVE PEAKS OF M/Z 134, M/Z 193 WHICH SHOWED THE PRESENCE OF FERULOYL GROUP the base peak m/z 413 indicated the presence of feruloyl group with some acidic moiety or other functional groups(kang etal 2016) SO FRPOM ABOVE FRAGMENTATION THE COMPOUND WAS IDENTIFIED AS

Kang, J., Price, W. E., Ashton, J., Tapsell, L. C., & Johnson, S. (2016). Identification and characterization of phenolic compounds in hydromethanolic extracts of sorghum wholegrains by LC-ESI-MSn. *Food chemistry*, *211*, 215-226

1. 3-[1,3-dihydroxy -3-(4-hydroxy-3-methoxy phenyl) propoxy]-2,3-dihydroxy propyl Ferulic acid.

The deprotonated molecular ion appeared at m/z 479 suffered the loss of 18 Da[M-H-H_2_O]^-^, 36 Da[M-H-2H_2_O]^-^ and 44 Da [M-H-CO_2_]^-^ Yielding m/z 461, m/z 443 and m/z 435. m/z 405, m/z 369, m/z 343, m/z 311 and m/z 281 were obtained from precursor ion by the loss of 74 Da[M-H-C_3_H_6_O_2_]^-^, 110 Da[M-H-C_6_H_6_O_2_]^-^, 136 Da[M-H-C_8_H_8_O_2_]^-^, 168 Da[M-H-C_9_H_12_O_3_]^-^ and 198 Da[M-H-C_10_H_14_O_4_]^-^. m/z 255, m/z 218, m/z 201, m/z 171 and m/z 149 were generated by the loss of 224 Da[M-H-C_10_H_8_O_6_]^-^, 261 Da[M-H-C_11_H_17_O_7_]^-^, 278 Da [M-H-C_12_H_22_O_7_]^-^, 308 Da[M-H-C_13_H_24_O_8_]^-^ and 330[M-H-C_14_H_18_O_9_]^-^m/z 443 regarding literature showed the presence of diferuloyl moiety with glycerol(kang et al., 2016) and molecular ion peak m/z 479 indicated the presence of derivative of diferuloyl glycerol.

Kang, J., Price, W. E., Ashton, J., Tapsell, L. C., & Johnson, S. (2016). Identification and characterization of phenolic compounds in hydromethanolic extracts of sorghum wholegrains by LC-ESI-MSn. *Food chemistry*, *211*, 215-226
