# Supplementary material for: Unraveling the bioactive constituents of Typha elephantina: A comprehensive phytochemical analysis by tandem mass spectrometry
Source: PLoS One. 2024 Dec 5;19(12):e0311549. doi: 10.1371/journal.pone.0311549 (PMC11620470; doi:10.1371/journal.pone.0311549)
Supplement: S3 File — (DOCX) [file pone.0311549.s003.docx]

**The compounds identified from *Typha elephantina* roots by Tandem mass spectrometry**

1. **2-Hydroxy Benzoic acid**

The quasi molecular ion peak [M-H]^-^ appeared at m/z 137 with the loss of 15Da [M-H-CH_3_radical]^-^ The m/z 122 obtained the m/z 109 and m/z 94 were produced due to the loss of 28Da [M-H-CO]^-^and 43Da [M-H-C_2_OH_3_]^-^The m/z 93(phenoxide ion) with the removal of [M-H-CO_2_]^-^indicated the presence of benzoic acid (Ali *et al*., 2021). The m/z 81, m/z 75 and m/z 65 were generated by the loss of 56 Da[M-H-2CO]^-^, 62 Da[M-H-COOH+OH]^-^and [M-H-C_2_O_3_]^-^ m/z 65 was the charachteristic of benzene group regarding reported literature and at m/z 75 the loss of COOH+OH showed the presence of benzoic acid with hydroxy group as a functional group the whole conversation and fragmentation suggested that compound (1) was tentatively identified as 2-hydroxy benzoic acid.

1. **4-Hydroxy Acetophenone (benzoic acid derivative)**

The deprotonated molecular ion peak for compound (2) appeared at m/z 151 with loss of 15Da[M-H-CH_3_]^-^m/z 136 initiated by homolytic cleavage m/z 133 was the cause of 18 Da [M-H-H_2_O]^-^among them the removal of 2Da provided m/z 131 the loss of 44Da[M-H-CO_2_]^-^ generated m/z 107 base peak m/z 93 was obtained by the sudsequent loss of 58Da [M-H-CO_2_+CH_2_^-^]^-^indicated the presence of benzoic acid derivative (Ali *et al*., 2021) among them the loss of 4Da produced m/z 89 m/z 79, m/z 71 and m/z 59 were generated by the loss 72Da[C_3_H_4_O_2_]^-^,80Da[M-H-C_5_H_4_O]^-^and 92Da[M-H-C_6_H_4_O]^-the^ fragmentation pathway prompted that compound (2) was tentatively considered as 4-Hydroxy Acetophenone (benzoic acid derivative).

1. **Protocatechuic acid**

Compound (3) displayed deprotonated molecular ion at m/z 153 the fragment ion at m/z 138 was produced by the removal of 15 Da [M-H-CH_3_]^-^and the 30Da [M-H-CO+H_2_]^-^corresponding to m/z 123 the base peak m/z 109(catechol) was assigned for 44Da [M-H-CO_2_]^-^showed the presence of benzoic acid moiety with two adjacent hydroxyl group the m/z 95 and m/z 93 were suffered the loss of 58Da[M-H-C_2_H_2_O_2_]^-^and 60Da[M-H-CO_2_+Oradical]^-^the daughter ion peaks m/z 83 and 69 were generated by removal of 70Da[M-H-C_3_H_2_O_2_]^-^and 84 Da[M-H-C_4_H_4_O_2_]^-^the base peak was matched with literature so according to reported literature the presence of protocatechuic acid was expected (kang *et al*., 2016) the fragmentation pattern proclaimed that compound (3) was tentatively assigned as protocatechuic acid.

1. ***p*-Coumaric Acid**

The deprotonated molecular ion [M-H]^-^ for compound (4) was produced at m/z 163 suffered the loss of 15Da [M-H-CH_3_]^-^ resulted m/z 148 the MS_2_ fragment ion m/z 135 was obtained with loss of 28Da[M-H-CO]^-^ base peak m/z 119 (4-hydroxy vinyl benzene) was generated via the removal of [M-H-CO_2_]^-^indicated the presence of coumaric acid (Sinosaki *et al*., 2020) fragment ions m/z 105, m/z 93 and m/z 83 were produced by the removal of 58Da[M-H-C_2_H_2_O_2_]^-^,70Da[M-H-C_3_H_2_O_2_]^-^and 80Da[M-H-C5H4O]^-^ m/z 93 was the charachteristic of phenoxide ion the m/z 75 and m/z 59 corresponding to 88Da[M-H-C_3_H_3_O_2_+OH] and 104Da[M-H-C_7_H_4_O]^-^ m/z 75 was the charahteristic of benzene according to base peak corresponding to reported literature(Sinosaki *et al*., 2020) (El-sayed *et al*., 2021) and fragmentation path way the compond(4) was plausibly described as *p*-coumaric acid.

1. **Mono hydrate of Gallic acid**

Compound (5) exhibited deprotonated precursor ion[M-H]^-^ at m/z 187 fragment ion m/z 172 was due to 15Da[M-H-CH_3_]^-^m/z 169 was responsible for 18Da[M-H-H_2_O]^-^m/z 159, m/z 143, m/z 141 were obtained with the loss of 28Da [M-H-CO]^-^,44Da[M-H-CO_2_]^-^and 46Da [M-H-CO_2_+H_2_]^-^ the loss of CO_2_ showed the presence acidic residue the base peak m/z 125 (Sinosaki *et al*., 2020) was resulted due to 62Da[M-H-COOH+OH]^-^indicated the presence of gallic acid among base peak m/z 123 and m/z 83 were obtained by the loss of 2Da(H_2_) and 42Da(C_2_H_2_O) m/z 107, m/z 97, m/z 73 and m/z 57 were generated with the loss of 80Da[M-H-CH_4_O_4_]^-^,90Da[M-H-C_3_H_6_O_3_]^-^,114Da[M-H-C_4_H_2_O_4_]^-^and 130 Da[M-H-C_5_H_6_O_4_]^-^the base peak m/z 125 according to published data(Dos *et al*., 2018)(Singh *et al*., 2016) showed the presence of gallic acid the loss of COOH+OH prompted the gallic acid in derivatized form so from whole entire fragmentation scheme or base peak evidence from literature the compound(5) was probably assigned as monohydrate of gallic acid.

m/z 187 monohydrate of gallic acid

1. Dihydrofurano coumarin

The Pseudo deprotonated molecular ion was appeared at *m/z* 202 the actual mass of the compound (6) was m/z 203 the m/z 188 and m/z 187 was obtained with the loss of 14 Da(CH_2_) and 15 Da(CH_3_^·^) The further fragmentation was proceed from m/z because the compound was considered adduct of dihydrocoumarin with the help of literature the other fragment ions m/z 174, m/z 166, m/z 158, m/z 146, and m/z 143 and were generated due to loss of 13 Da(CH), 21 Da (H_2_O+H_2_+H^·^), 29 Da(CHO), 41 Da (C_2_HO), 44 Da (CO_2_) and m/z 140 was obtained due to loss of 3Da(H_2_+H^·^) from m/z 143 the product ions m/z 126, m/z 116, m/z 115, m/z 94, m/z 88 and m/z 71 were resulted with the loss of 61 Da(C_2_H_5_O_2_), 71 Da(C_3_H_3_O_2_), 72 Da(C_3_H_4_O_2_), 93 Da(C_5_HO_2_), 99 Da(C_4_H_3_O_3_), 116 Da(C_8_H_4_O) from literature m/z 187, m/z 174, m/z 146 were the characteristic of dihydrofurano coumarin so the compound (6) was considered methylated adduct of dihydrofurano coumarin.

Tine, Y., Renucci, F., Costa, J., Wélé, A., & Paolini, J. (2017). A method for LC-MS/MS profiling of coumarins in Zanthoxylum zanthoxyloides (Lam.) B. Zepernich and Timler extracts and essential oils. *Molecules*, *22*(1), 174.

Adimule, V. M., Nandi, S. S., Kerur, S. S., Khadapure, S. A., & Chinnam, S. (2022). Recent advances in the one-pot synthesis of coumarin derivatives from different starting materials using nanoparticles: a review. *Topics in Catalysis*, 1-31.

1. **Methyl ferulate**

The deprotonated molecular ion appeared at m/z 207 the actual mass of the compound (7) was 208 *a.m.u.* the fragment ions m/z 205, m/z 192, m/z 189, m/z 179, m/z 177, m/z 163 and m/z 161 were generated with loss of 2 Da [M-H-H_2_]^-^, 15 Da[M-H-CH_3_]^-^, 18 Da [M-H-H_2_O]^-^, 28 Da [M-H-CO]^-^, 30 Da [M-H-CH_2_O]^-^, 44 Da [M-H-CO_2_]^-^, and 46 Da[M-H-C_2_H_6_O]^-^and other fragment ions m/z 145, m/z 135, m/z 122, m/z 119, m/z 109, m/z 93, m/z 85 and m/z 71 were appeared due to loss of 62 Da [M-H-C_2_H_6_O_2_]^-^, 72 Da [M-H-C_3_H_4_O_2_]^-^, 85 Da [M-H-C_4_H_5_O_2_]^-^, 88 Da [M-H-C_3_H_4_O_3_]^-^, 98 Da [M-H-C_5_H_6_O_2_]^-^, 114 Da [M-H-C_5_H_6_O_3_]^-^, 122 Da[M-H-C_7_H_6_O_2_]^-^, and 136 Da[M-H-C_8_H_8_O_2_]^-^ from all over fragmentation pattern the compound (7) was considered as derivative of ferulic acid.

1. **Dihydrate of Caffeic Acid**

The deprotonated pseudomolecular ion at m/z 215 crresponding to compound (8) yielding m/z 213, m/z 197 and m/z 187 with the loss of 2 Da [M-H-H_2_]^-^,18 Da [M-H-H_2_O]^-^ and 28Da[M-H-CO]^-^The base peak m/z 179 was the cause of 36Da[M-H-2H_2_O]^-^(Sinosaki *et al*., 2020) showed presence of caffeic acid among them m/z 161, m/z 153 and m/z 143 were generated with the loss of 18Da(H2O), 26Da(2CH) and 36Da(2H_2_O)the m/z 161 and m/z 143 were the charachteristic fragment ion peaks of caffeic acid (Kang *et al*., 2016) m/z 131 and m/z 119 were obtained by the loss of 48Da(CO_2_+2H_2_) and 60Da(CO+Oradical) from m/z 119 the loss of 18Da(H_2_O) and 30Da(CH_2_O) resulted m/z 101 and m/z 89 from base peak m/z 179 the removal of 108 Da(C_6_H_4_ O_2_) gave m/z 71 from all above justification and fragmentation pattern the compound (8) was supposedly assigned as Dihydrate of caffeic acid.

1. **Dihydrate of dihydrocaffeic acid**

The deprotonated adduct ion for compound (9) was displayed at m/z 217 suffered the loss of 2Da[M-H-H_2_]^-^_,_15Da[M-H-CH_3_]^-^,18 Da [M-H-H_2_O]^-^, and 36Da[M-H-2H_2_O]^--^yielding m/z 215, m/z 202,m/z 199, m/z and m/z 181 showed the presence of dihydrocaffeic acid among them the loss of 2Da initiated base peak m/z 179 (Sinosaki *et al*., 2020) indicated the presence of caffeic acid among them the m/z 161, m/z 143, m/z 131, m/z 119 and m/z 71 were generated by the subsequent lossess of 18Da(H_2_O), 36Da(2H_2_O), 48Da(CO_2_+2H_2_), 60Da(CO_2_+Oradical) and 108Da(C_6_H_4_O_2_) m/z 161and m/z 143 were the characteristic daughter ion peaks of caffeic acid m/z 119 represented the 4-hydroxy vinyl benzene from precursor ion the loss of 44Da[M-H-CO_2_]^-^gave m/z 173 and from them removal of 2Da provided m/z 171 from m/z 119 the loss of 18Da(H_2_O) resulted m/z 101 suffered the loss of 12Da yielding m/z 89 and from them the loss of 24Da gave m/z 65 was also the charachteristic of benzene the by observing fragmentation procedure it was assumed that caffeic acid is present in derivatized form so the compound (9) was probably considered as dihydrate of caffeic acid.

1. **4-Hydroxy-3 Methoxy-4(3,4,5-Trioxo-Tetrahydro Furan) Butanal**

Compound (10) showed deprotonated molecular ion at m/z 229 with the loss of 16Da(O) gave m/z 213 the loss of 18Da[M-H-H_2_O]^-^initiated m/z 211. m/z 201,m/z 193, m/z 185 and m/z 167 were generated with the loss of 28Da[M-H-CO]^-^,36Da[M-H-2H_2_O]^-^,44Da[M-H-CO_2_]^-^and 62Da[M-H-CO_2_+H_2_O]^-^ fragment ions m/z 147, m/z 144, m/z 119 and m/z 109 were obtained by the loss of 82Da[M-H-C_4_H_2_O_2_]^-^,86Da[M-H-C_4_H_6_O_2_]^-^,110Da[M-H-CH_2_O_6_]^-^and120Da[M-H-C_2_O_6_]^-^from m/z 109 the loss of 14Da produced m/z 95 from them the loss of 12Da and 24Da bring out m/z 83 and m/z 71 from whole fragmentation method and base peak regarding literature (Cioffi et al.,2000) it was suggested that compound (10) was plausibly identified as derivative of dehydroascorbic acid.

1. Cioffi, N., Losito, I., Terzano, R., & Zambonin, C. G. (2000). An electrospray ionization ion trap mass spectrometric (ESI-MS-MSn) study of dehydroascorbic acid hydrolysis at neutral pH. *Analyst*, *125*(12), 2244-2248.
2. **1*-O*-Coumaroyl Glycerol**

The compound (11) exhibited quasi molecular ion peak at m/z 237 in MS_3_ yielding m/z 223, m/z 205, m/z 194 and m/z 177 with the loss of 15Da[M-H-CH_3_]^-^,32Da[M-H-O_2_]^-^,44Da[M-H-CO_2_]^-^and m/z 60Da[M-H-C_2_H_4_O_2_]^-^m/z 163 was the cause of 74Da[M-H-C_3_H_6_O_2_]^-^was the characteristic of coumaric acid among them the loss of 2Da produced m/z 161 from it the loss of 12Da gave m/z 149 from precursor ion the loss of 118Da[C_4_H_6_O_4_]^-^yielding m/z 119 the base peak was the characteristic of 4-hydroxy vinyl benzene from them the loss of 2Da bring out m/z 117 the m/z 97 and m/z 81 were resulted by the loss of 140Da[M-H-C_8_H_12_O_2_]^-^and 156Da[M-H-C_7_H_8_O_4_]^-^ according to above discussion from observation of fragmentation method it was proclaimed that compound(11) was tentatively considered as 1*-O*-Coumaroyl Glycerol

1. **Daidzen (7,4′ di Hydroxy Iso Flavone)**

Compound (12) showed dprotonated ion at m/z 253 suffered the loss of 2Da[M-H-H_2_]^-^, 18Da[M-H-H_2_O]^-^,28Da[M-H-CO]^-^and 44Da[M-H-CO_2_]^-^yielding m/z 251, m/z 235, m/z 225 and m/z 209 from m/z 225 base peak the loss of 28Da(CO) gave m/z 197 from m/z 209 the loss of 28Da(CO) yielded m/z 181 among them the removal of 14Da brought m/z167 from precursor ion the loss of 96Da[M-H-C_6_H_8_O]^-^yielded m/z 158 from m/z 167 the removal of 14Da gave m/z 153 from parent ion m/z 135, m/z 123, m/z 97, m/z 85 and m/z 75 were generated by the loss of 118Da[M-H-C_8_H_6_O]^-^, 130Da[M-H-C_8_H_2_O_2_]^-^, 156Da[M-H-C_7_H_8_O_4_]^-^, 168Da[M-H-C_10_O_3_]^-^ and 178Da[M-H-C_9_H_6_O_4_]^-^ the base peak m/z 225 was matched with literature and m/z 209, m/z 197, m/z 181 and m/z 135 were the charachteristic of diadzen (kang *et al*., 2007) from whole fragmentation pattern and base peak it was decided to supposedly assigned compound(12) as diadzen.

Kang, J., Hick, L. A., & Price, W. E. (2007). A fragmentation study of isoflavones in negative electrospray ionization by MSn ion trap mass spectrometry and triple quadrupole mass spectrometry. Rapid Communications in Mass Spectrometry: An International Journal Devoted to the Rapid Dissemination of Up-to-the-Minute Research in Mass Spectrometry, 21(6), 857-868.

1. **Pinocembrin**

The deprotonated molecular ion appeared at m/z 255 the actual mass of the compound (13) was 256 *a.m.u.* The fragment ions m/z 253, m/z 240, m/z 223, m/z 211, m/z 195, m/z 181 and m/z 175, m/z 167, and m/z 151 were generated due to loss of 2 Da [M-H-H_2_]^-^, 15 Da [M-H-CH_3_^·^], 32 Da [M-H-O_2_]^-^, 44 Da[M-H-CO_2_]^-^, 60 Da[M-H-CO2+O^·^]^-^, 74 Da[M-H-C_6_H_2_]^-^, 80 Da[M-H-C_4_O_2_]^-^, 88 Da[M-H-C_7_H_4_]^-^, and 104 Da[M-H-C_8_H_8_]^-^m/z 127, m/z 109, m/z 93 and m/z 85 were obtained with the loss of 128 Da[M-H-C_9_H_4_O]^-^, 146 Da[M-H-C_9_H_6_O_2_]^-^, 162 Da[M-H-C_9_H_6_O_3_]^-^and 170 Da[M-H-C_11_H_6_O_2_]^-^from whole fragmentation pattern and with the help of literature the compound (13) was considered as pinocembrin.

Bertrams, J., Kunz, N., Müller, M., Kammerer, D., & Stintzing, F. C. (2013). Phenolic compounds as marker compounds for botanical origin determination of German propolis samples based on TLC and TLC-MS. *Journal of Applied Botany and Food Quality*, *86*(1),143-153.

Zhao, X., Zhang, S., Liu, D., Yang, M., & Wei, J. (2020). Analysis of flavonoids in dalbergia odorifera by ultra-performance liquid chromatography with tandem mass spectrometry. *Molecules*, *25*(2), 38

1. **Genistein (5,7,4′ tri Hydroxy Isoflavone)**

Compound (14) displayed deprotonated molecular ion at m/z 269 the actual mass of the compound was 270 *a.m.u.* [M-H]^-^generated *m/z* 267, *m/z* 254, *m/z* 241 and *m/z* 225 with the loss of 2 Da [M-H-H_2_]^-^, 15 Da [M-H-CH_3_^·^]^-^, 28 Da [M-H-CO]^-^ and 44 Da [M-H-CO_2_]^-^. The loss of 14 Da (CH_2_) , 28 Da (CO) from *m/z* 225 gave *m/z* 211 and *m/z* 197. The loss of 2 Da (H_2_) from *m/z* 197 brought *m/z* 195. *m/z* 173, *m/z* 159 and m/z 153 were resulted from precursor ion due to loss of 96 Da [M-H-C_6_H_8_O]^-^, 110 Da [M-H-C_6_H_6_O_2_]^-^ and 116 Da [M-H-2O_2_+C_4_H_4_]^-^ . m/z 151, m/z 131, m/z 121, m/z 97 and m/z 93 were generated by the loss of 118 Da [M-H-C_8_H_6_O]^-^, 138 Da [M-H-C_7_H_6_O_3_]^-^,148 Da[M-H-C_9_H_8_O_2_]^-^, 172 Da [M-H-C_10_H_4_O_3_]^-^ and [M-H-C_9_H_4_O_4_]^-^.so according to literature the *m/z* 269, *m/z* 241, *m/z* 225, *m/z* 197 and *m/z* 151 were the characteristic of genistein (Zhao *et al*., 2018) and fragmentation pattern the compound (14) was considered as Genistein.

Zhao, W., Shang, Z., Li, Q., Huang, M., He, W., Wang, Z., & Zhang, J. (2018). Rapid screening and identification of daidzein metabolites in rats based on UHPLC-LTQ-orbitrap mass spectrometry coupled with data-mining technologies. Molecules, 23(1), 151.

1. **1-*O*-coumaroyl glycerol derivative (1-*O*-coumaroyl 2,3 dihydroxy butanoic acid)**

The deprotonated molecular ion appeared at m/z 281 the actual mass of compound (15) was 282 *a.m.u.* The fragment ions m/z 266, m/z 263, m/z 249, m/z 237 and m/z 233 were generated due to the loss of 15 Da[M-H-CH_3_^·^]^-^, 18 Da[M-H-H_2_O]^-^, 32 Da[M-H-O_2_]^-^, 44 Da[M-H-CO_2_]^-^ and 48 Da[M-H-CO_2_+2H_2_]^-^The other fragment ions m/z 219, m/z 207, m/z 193, m/z 182 and m/z 163 were obtained with the loss of 62 Da[M-H-COOH+OH^·^]^-^, 74 Da[M-H-C_2_H_2_O_3_]^-^, 88 Da[M-H-COOH+CO_2_]^-^ 99 Da [M-H-C_2_H_11_O_4_]^-^ and 118 Da [M-H-C_4_H_6_O_4_]^-^ and fragment ions m/z 145, m/z 123, m/z 117, m/z 97 and m/z 88 were resulted by the loss of 136 Da[M-H-C_4_H_8_O_5_]^-^,158 Da[M-H-C_5_H_2_O_6_]^-^, 164 Da [M-H-C_5_H_8_O_6_]^-^, 184 Da[M-H-C_7_H_4_O_6_]^-^ and 194 Da [M-H-C_9_H_10_O_2_+CO_2_]^-^the base peak appeared at m/z 237 by matching with literature kang et al so it was considered as derivative of 1-*O*-coumaroyl glycerol derivative.

1. **Catechin**

The [M-H]^-^ was appeared at m/z 289 the actual mass of the compound (16) was 290 searched in literature the molecular ion peak and base peak matched with Catechin (Kang *et al*., 2016) the m/z 271 was obtained by the loss of 18Da H_2_O one water molecule and the the loss of 30 Da CH_2_O gave peak at m/z 259 the m/z 247 was justified by the loss of 42 Da C_2_H_2_O the m/z 245 was obtained by the loss of 44 Da C_2_H_4_O the m/z 231 was justified by the loss of 14 Da CH_2_ and the loss of 36 Da 2H_2_O two water molecule from m/z 245 gave peak at m/z 209 the loss of 84 Da C_4_H_4_O_2_ from m/z 289 gave peak at m/z 205 the loss of 86 Da C_4_H_6_O_2_ gave peak at m/z 203 the m/z 187 was obtained by the loss of 102 Da C_4_H_6_O_3_ the m/z 179 was justified by the loss of 110 Da C_6_H_6_O_2_ and m/z 165 was obtained by the loss of 124 Da C_7_H_8_O_2_ the peak m/z 137 was justified by the loss of 152 Da C_8_H_8_O_3_ from molecular ion peak 289. The m/z 125 was obtained by the loss of 164 Da C_9_H_8_O_3_ the m/z 109 was appeared due to the loss of 180 Da C_9_H_8_O_4_ the m/z 97 was obtained by the loss of 193 Da C_10_H_9_O_4_ the m/z 83 was justified by the loss of 14 Da CH_2_ from m/z 97 The compound was tentatively identified as Catechin.

**Reference**

Kang, J., Price, W. E., Ashton, J., Tapsell, L. C., & Johnson, S. (2016). Identification and characterization of phenolic compounds in hydromethanolic extracts of sorghum wholegrains by LC-ESI-MSn. *Food chemistry*, *211*, 215-226.

1. **DihydroQuercetin (3,5,7, 3′,4′ Pentahydroxy Flavanonol) (Taxifolin Isomer)**

Ye M, Yang wz, Liu KD, Qiao X, Li BJ, Cheng J and Zhao YY. Characterization of flavonoids in Millettia nitida var. hirsutissima by HPLC/DAD/ESI-MSn. J of Pharm Ana. 2012;2(1):35-42.

The deprotonated molecular ion peak for compound (17) appeared at m/z 303 the actual mass of the compound was 304 *a.m.u.* *m/z* 287, *m/z* 285, *m/z* 267, *m/z* 257 and *m/z* 241 with the loss of 16 Da [M-H-O^·^]^-^,18 Da[M-H-H_2_O]^-^,36 Da [M-H-2H_2_O]^-^, 46 Da[M-H-CO_2_+H_2_]^-^, 62 Da [M-H-C_2_H_2_O_2_+2H_2_] *m/z* 241 with loss of 16 Da(O^·^) and 24 Da (2C) brought *m/z* 225 and *m/z* 217. m/z 217 with the loss of 4 Da (2H_2_) gave *m/z* 213. *m/z* 199, *m/z* 185, *m/z* 175 and *m/z* 157 were resulted by the loss of 104 Da [M-H-C_4_H_8_O_3_]^-^, 118 Da [M-H-C_7_H_2_O_2_]^-^, 128 Da [M-H-C_6_H_8_O_3_]^-^and146 Da [M-H-3O_2_+C_4_H_2_]^-^, m/z 157 with loss of 16 Da (O^·^) gave m/z 141. m/z 141 brought m/z 113 with the loss of 28 Da (C_2_H_4_). m/z 113 gave m/z 111 with the loss of 2 Da (H_2_). According to literature (Ye et al., 2012) m/z 303, m/z 241, m/z 285, m/z 217, m/z 213 and m/z 175 were the characteristic of dihydro quercetin so the compound (17) was assigned as dihydroquercetin.

1. **Ampelopsin , Dihydromyricetin (3,5,7, 3′,4′,5′ Hexa Hydroxy Flavanonol)**

The deprotonated molecular ion appeared at m/z 319 the actual mass of the compound was 320 a.m.u. m/z 304, m/z 301, m/z 287, m/z 275, m/z 257, m/z 239, m/z 224 and m/z 197 resulted by the loss of 15 Da [M-H-CH_3_]^-^, 18 Da [M-H-H_2_O]^-^, 32 Da [M-H-O_2_]^-^, 44 Da [M-H-CO_2_]^-^, 62 Da [M-H-CO+O_2_+H_2_]^-^, 80 Da [M-H-2O2+O^·^]^-^, 95 Da [M-H-C_5_H_3_O_2_]^-^ and 122 Da [M-H-C_6_H_2_O_3_]^-^. m/z 239 gave m/z 177 with the loss of 62 Da(CO+O_2_+H_2_) Precursor ion with the loss of 154 Da[M-H-C7H6O4]- gave m/z 165. m/z 275 by the loss of 130 Da (C_6_H_10_O_3_) brought m/z 145. The m/z 125 was obtained with the loss of 194 Da[M-H-C9H6O5]- from deprotonated ion and m/z 125 brought m/z 107 and m/z 97 with the loss of 18 Da(H2O) and 28 Da(CO) according to literature m/z 319, m/z 301, m/z 257 and m/z 125 were the characteristic of ampelopsin so the compound (18) was assigned as ampelopsin.

1. **6-*O*-Caffeoyl glucoside dihydrate**

The deprotonated ion appeared at m/z 377 the actual mass of the compound was 378 *a.m.u.* m/z 359, m/z 345, m/z 341 and m/z 333 with the loss of 18 Da [M-H-H_2_O]^-^, 32 Da[M-H-O2]-, 36 Da[M-H-2H2O]-and 44 Da[M-H-CO2]-. m/z 341 brought m/z 315, m/z 297 and m/z 279 by the loss of 26 Da [C_2_H_2_], 44 Da [CO_2_] and 62 Da [C2H5O2]-. m/z 377 gave m/z 245 with the loss of 132 Da [M-H-C5H8O4]-. m/z 245 brought m/z 221 by the loss of 24 Da [2C]. m/z 377 with the loss of 162 Da[M-H-C6H10O5] gave m/z 215. m/z 215 with the loss of 18 Da [H2O] produced m/z 197 m/z 377 with the loss of 162 Da+36 Da [M-H-C6H10O5+2H2O]- m/z 179 from m/z 179 the loss of 18 Da [H2O] and 42 Da[C2H2O] m/z 161 and m/z 137. m/z 137 gave m/z 113 with the loss of 26 Da[C2H2] according to literature the kang et al., 2016 the m/z 341 was the charachteristic of caffeoyl glycoside and m/z 179, m/z 161 were the characteristic of caffeic acid so the compound was considered as 6-*O*-caffeoyl glucoside dihydrate.

1. **Feruloyl methyl caffeic acid**

The deprotonated peak of compound appeared at m/z 385 with the loss of 18 Da[M-H-H_2_O]^-^the product ion m/z 367 was produced the m/z 349, m/z 341, m/z 325, m/z 305 and m/z 303 were generated with significant lossess of 36 Da [M-H-2H_2_O]^-^, 44 Da [M-H-CO_2_]^-^, 60 Da [M-H-COOH+CH_3_^·^]^-^, 80 Da[M-H-COOH+OH^·^+H_2_O]^-^, and 82 Da[M-H-C_4_H_2_O_2_]^-^and m/z 293, m/z 269, m/z 267 ,m/z 259 m/z 231 and m/z 217 were obtained upon the following lossess 92 Da [M-H-OCH_3_^·^+OH^·^+H_2_O]^-^, 116 Da [M-H-C_5_H_4_O_2_+H_2_O+H_2_]^-^, 118 Da [M-H-C_5_H_6_O_2_+H_2_O+H_2_]^-^, 126 Da [M-H-C_7_H_10_O_2_]^-^, 154 Da [M-H-C_8_H_10_O_3_]^-^ and 168 Da[M-H-C_9_H_12_O_3_]^-^the other product ion or fragment ions m/z 203, m/z 190, m/z 179, m/z 163, m/z 145, m/z 139 and m/z 117 were generated with the loss of 182 Da[M-H-C_10_H_14_O_3_]^-^, 195 Da[M-H-C_10_H_11_O_4_]^-^, 206 Da[M-H-C_11_H_10_O_4_]^-^, 222 Da [M-H-C_11_H_10_O_5_]^-^, 240 Da [M-H-C_11_H_11_O_5_+OH^·^]^-^, 246 Da [M-H-C_13_H_10_O_5_]^-^and 268 Da [M-H-C_14_H_4_O_6_]^-^ m/z 203, m/z 190, m/z 163 and m/z 145 were the characteristic peaks of ferulic acid. From deprotonated molecular ion peak and whole fragment ion peaks indicated the presence of diferulic acid so from above discussion the compound was identified as 1,3-*O*-diferulate.

1. **Feruloyl methyl dihydrocaffeic acid**

The deprotonated molecular ion peak appeared at m/z 387 the 1^st^ fragment ion peak obtained with the loss of 18 Da[M-H-H_2_O]^-^ at m/z 369. m/z 343, m/z 317, m/z 305, m/z 269, m/z 235, and m/z 203 were produced due to the loss of 44 Da[M-H-CO_2_]^-^, 70 Da[M-H-C_3_H_2_O_2_]^-^, 82 Da[C_4_H_2_O_2_]^-^,118 Da[M-H-C_5_H_6_O_2_+H_2_O+H_2_]^-^, 152 Da[M-H-C_8_H_8_O_3_]^-^ and 184 Da[M-H-C_9_H_12_O_4_]^-^ and other fragment ions m/z 190,m/z 179, m/z 163, m/z 145, m/z 132 and m/z 119 were generated upon the significant lossess of 197 Da[M-H-C_10_H_13_O_4_]^-^, 208 Da[M-H-C_11_H_12_O_4_]^-^, 224 Da[M-H-C_11_H_12_O_5_]^-^, 242 Da[M-H-C_11_H_14_O_6_]^-^, 255 Da[M-H-C_12_H_15_O_6_] and 268 Da[M-H-C_11_H_11_O_5_+COOH]^-^ m/z 203, m/z 190, m/z 179, m/z 163, and m/z 145 were the charachteristic of feruloyl group and m/z 387 deprotonated molecular ion peak indicated the presence of dihydroferuloyl so the compound was tentatively considered as **Feruloyl methyl dihydrocaffeic acid**

.

1. **Feruloyl methyl ethenyl caffeoate**

The deprotonated molecular ion of compound was displayed at m/z 411 the actual mass of compound was 412 *a.m.u* the m/z 393, m/z 367, m/z 349, m/z 331, m/z 287, m/z 259,m/z 245 and m/z 217 were initiated by the loss of 18 Da[M-H-H_2_O]^-^, 44 Da [M-H-CO_2_]^-^, 62 Da[M-H-C_2_H_4_O+H_2_O]^-^, 80 Da [M-H-C_3_H_12_O_2_]^-^124 Da[M-H-C_7_H_8_O_2_]^-^ 152 Da [M-H-C_9_H_12_O_2_]^-^, 166 Da[M-H-C_9_H_10_O_3_]^-^and 194 Da[M-H-C_10_H_10_O_4_]^-^And other fragment ions were appeared at m/z 203, m/z 190, m/z 176, m/z 152 and m/z 134 upon significant loss of 208 Da[M-H-C_11_H_12_O_4_]^-^, 221 Da[M-H-C_12_H_13_O_4_]^-^, 235 Da [M-H-C_12_H_11_O_5_]^-^, 252 Da[M-H-C_13_H_7_O_6_]^-^and 277 Da[M-H-C_14_H_13_O_6_]^-^ the fragment ions m/z 203 and m/z 190 and m/z 134 were the charachteristic of ferulic group the compound was considered as (1-*O*-ethene)1,3-*O*-diferulate.

1. 1-*O*-Feruloyl-3-*O*-*p*-Coumaroyl Glycerol

The deprotonated molecular ion peak appeared at m/z 413 the actual mass of the compound was 414 *a.m.u*. fragment ions m/z 398, m/z 369, m/z 345, m/z 327, m/z 315 and m/z 303 were obtained with the loss of 15 Da[M-H-CH_3_^·^]^-^, 44 Da[M-H-CO_2_]^-^, 68 Da[M-H-C_4_H_4_O]^-^, 86 Da[M-H-C_4_H_6_O_2_]^-^, 98 Da[M-H-C_5_H_6_O_2_]^-^, and 110 Da[M-H-C_6_H_6_O_2_]^-^ m/z 273, m/z 259, m/z 245, m/z 235, m/z 217 and m/z 203 were generated with the loss of 140 Da [M-H-C_8_H_8_O_2_+2H_2_]^-^, 154 Da [M-H-C_8_H_8_O_2_+H_2_O]^-^, 168 Da [M-H-C_9_H_10_O_2_+H_2_O]^-^, 178 Da[M-H-C_10_H_10_O_3_]^-^, 196 Da[M-H-C_10_H_12_O_4_]^-^, 210 Da[M-H-C_11_H_14_O_4_]^-^ m/z 190, m/z 177, m/z 161, m/z 152 and m/z 135 were produced due to loss of 223 Da[M-H-C_12_H_15_O_4_]^-^, 236 Da[M-H-C_12_H_12_O_5_]^-^, 252 Da[M-H-C_12_H_12_O_6_]^-^, 261 Da [M-H-C_13_H_9_O_6_]^-^and 278 Da[M-H-C_14_H_14_O_6_]^-^. The m/z 398, m/z 369, m/z 235, m/z 217, m/z 177, m/z 161 and m/z 135 were the characteristic peaks of 1-*O*-Feruloyl-3-*O*-*p*-Coumaroyl Glycerol.

1. **ISO Vitexin**

The deprotonated molecular ion peak displayed at m/z 431 the actual mass of the compound was 432 *a.m.u.* the fragment ions m/z 416, m/z 413, m/z 401, m/z 387, m/z 373, m/z 345, m/z 313 and m/z 305 were generated by the loss of 15 Da[M-H-CH_3_]^-^, 18 Da[M-H-H_2_O]^-^, 30 Da[M-H-CH_2_O]^-^, 44 Da[M-H-CO_2_]^-^,58 Da [M-H-C_2_H_2_O_2_]^-^ , 86 Da [3CO+H_2_]^-^, 118 Da [M-H-C_4_H_6_O_4_]^-^, and 126 Da[M-H-C_4_H_8_O_4_+3H_2_]^-^. The other fragment ions m/z 277, m/z 261, m/z 241, m/z 218, and m/z 187 were resulted with the loss of 154 Da [M-H-C_5_H_14_O_5_]^-^, 170 Da [M-H-C_7_H_6_O_5_]^-^, 190 Da [M-H-C_6_H_6_O_7_]^-^, 218 Da [M-H-C_12_H_5_O_4_]^-^ and 244 Da[M-H-C_10_H_12_O_7_]^-^ m/z 167 was obtained with the loss 220 Da [C_9_H_16_O_6_]^-^ from m/z 387 m/z 149 was initiated upon the loss of 112 Da [C_5_H_4_O_3_]^-^from m/z 261 the last fragment ion m/z 125 the base peak appeared due to loss 306 Da[M-H-C_15_H_14_O_7_]^-^. From literature and fragmentation scheme the compound was identified as Isovitexin.

1. **6-*O*-Pentenoyl glucopyranosyl-6-*C*-apigenin,**

The deprotonated molecular ion appeared at m/z 513 the actual mass of the compound was 514 *a.m.u*. the m/z 495, m/z 477, m/z 469, m/z 455, m/z 433, m/z 395 and m/z 377 were resulted with the loss of 18 Da [M-H-H_2_O]^-^, 36 Da [M-H-2H_2_O]^-^, 44 Da[M-H-CO_2_]^-^, 58 Da[3H_2_O+2H_2_]^-^, 80 Da[M-H-C_5_H_4_O]^-^, 118 Da[M-H-C_8_H_6_O]^-^ and 136 Da[M-H-C_8_H_8_O_2_]^-^ the other product ions m/z 349, m/z 329, m/z 293, m/z 283, m/z 255, m/z 233, m/z 191 and m/z 165 were produced due to loss of 164 Da[M-H-C_9_H_8_O_3_]^-^, 184 Da[M-H-C_9_H_12_O_4_]^-^, 220 Da [M-H-C_10_H_20_O_5_]^-^, 230 Da[M-H-C_10_H_14_O_6_]^-^, 258 Da[M-H-C_12_H_18_O_6_]^-^, 280 Da [M-H-C_13_H_12_O_7_]^-^, 322 Da [M-H-C_15_H_14_O_8_]^-^and 348 Da [M-H-C_17_H_16_O_8_]^-^. The compond was considered as 6-*O*-Pentenoyl glucopyranosyl-6-*C*-apigenin,

26. Procyanidin B1

The deprotonated molecular ion peak was appeared at m/z 577 the actual mass of the compound was 578 *a.m.u.* the fragment ions m/z 562, m/z 559, m/z 541, m/z 533, m/z 503, m/z 485 and m/z 471 were produced with the loss of 15 Da[M-H-CH_3_^·^], 18 Da [M-H-H_2_O]^-^, 36 Da[M-H-2H_2_O]^-^, 44 Da[M-H-CO_2_]^-^, 74 Da [M-H-C_3_H_6_O_2_]^-^, 92 Da [M-H-2CO+2H_2_O]^-^, 106 Da[C_6_H_2_O_2_]^-^and other fragment ions m/z 451, m/z 425, m/z 407, m/z 381, m/z 357, m/z 331, m/z 299, m/z, m/z 289, m/z 245, m/z 199 and m/z 175 were generated by the loss of 126 Da[M-H-C_6_H_6_O_3_]^-^, 152 Da [M-H-C_8_H_8_O_3_]^-^, 170 Da[M-H-C_8_H_10_O_4_]^-^, 196 Da[M-H-C_10_H_12_O_4_]^-^, 220 Da[M-H-C_11_H_8_O_5_]^-^, 246 Da[M-H-C_13_H_10_O_5_]^-^,278 Da[M-H-C_14_H_14_O_6_]^-^, 288 Da[M-H-C_15_H_12_O_6_]^-^, 332 Da [M-H-C_15_H_12_O_6_+CO_2_]^-^, 378 Da [M-H-C_19_H_22_O_8_]^-^and 402 Da [M-H-C_21_H_22_O_8_]^-^by matching base peak and deprotonated molecular ion peak and fragmentation pattern the compound was considered as procyanidin dimer B.
