# Supplementary figures and images for: Unraveling the bioactive constituents of Typha elephantina: A comprehensive phytochemical analysis by tandem mass spectrometry
Source: PLoS One. 2024 Dec 5;19(12):e0311549. doi: 10.1371/journal.pone.0311549 (PMC11620470; doi:10.1371/journal.pone.0311549)

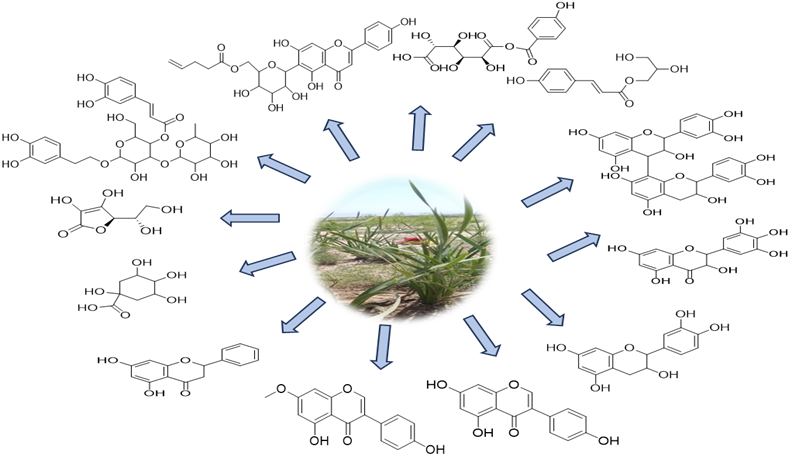

Supplement: S1 Graphical abstract — (PNG) [file pone.0311549.s004.png]
